# Medicinal Perspective of 2,4‐Thiazolidinediones Derivatives: An Insight into Recent Advancements

**DOI:** 10.1002/open.202400147

**Published:** 2024-09-09

**Authors:** Sneha Gupta, Sumeet Jha, Supriya Rani, Pinky Arora, Shubham Kumar

**Affiliations:** ^1^ School of Pharmaceutical Sciences Lovely Professional University Jalandhar-Delhi G.T. Road Phagwara Punjab 144411 India; ^2^ School of bioengineering and biosciences Lovely Professional University Jalandhar-Delhi G.T. Road Phagwara Punjab 144411 India

**Keywords:** 2,4-Thiazolidinedione, Review, Mechanism of Action, Recent Advances, Anticancer, Antimicrobial

## Abstract

2,4‐Thiazolidinedione derivatives represent nitrogen‐containing heterocyclic compounds utilized in type 2 diabetes mellitus management. Recent advances in medicinal chemistry have unveiled diverse therapeutic potentials and structural modifications of these derivatives. This review delves into novel TZD derivatives, encompassing their synthesis, structure‐activity relationships, and pharmacokinetic profiles. Various therapeutic potentials of TZDs are explored, including anticancer, antimicrobial, anti‐inflammatory, antioxidant, anticonvulsant, antihyperlipidemic, anticorrosive, and antitubercular activities. Additionally, it addresses mitigating side effects associated with marketed TZD derivatives such as weight gain, oedema, fractures, and congestive heart failure in type 2 diabetes mellitus management. The review elaborates on *in vivo, in vitro*, and *ex vivo* studies supporting different biological activities, alongside predicting ADME and drug‐likeness properties of TZDs. Computational studies are also integrated to elucidate binding modes and affinities of novel TZD derivatives. Furthermore, a plethora of novel TZD derivatives with varied and enhanced therapeutic potentials are presented, warranting further evaluation of their biological activities.

## Graphical abstract

This article reviews recent advancements in the medicinal chemistry of 2,4‐thiazolidinedione derivatives, focusing on their synthesis, characterization, and therapeutic potential. Emphasis is placed on their roles in treating various diseases, highlighting their significance in medicinal chemistry and future perspectives.

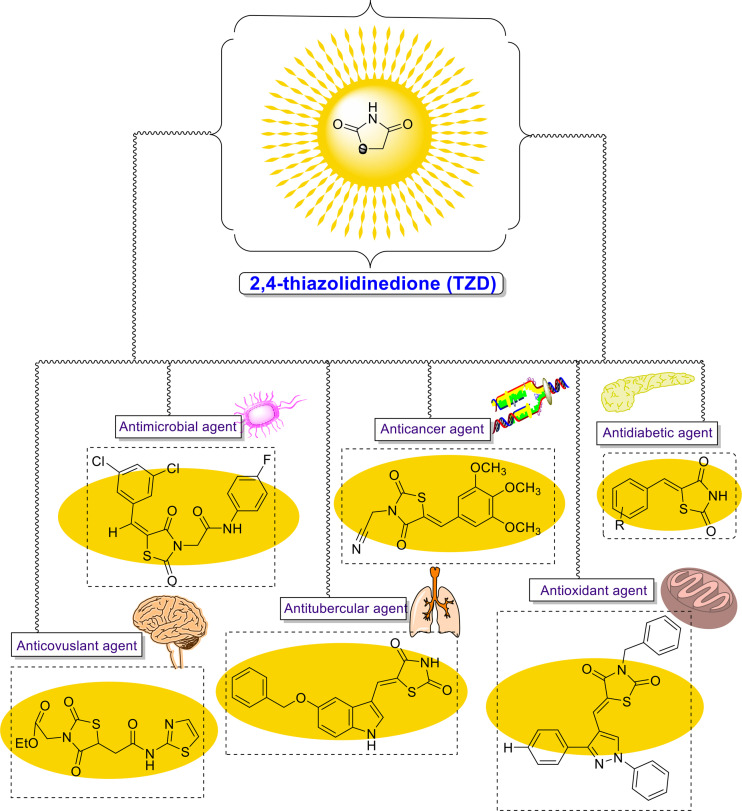

## 1 Introduction

A heterocyclic moiety is a component of a molecule that contains a ring structure with carbon atoms and at least one heteroatom, which refers to an atom other than carbon.[^1^, ^2^] The most common heteroatoms found in heterocyclic compounds are oxygen, nitrogen, sulfur, and phosphorus. Heterocyclic compounds vary in size and complexity and are widely present in nature.[^3^, ^4^, ^5^, ^6^] These compounds play a significant role in the field of organic chemistry.[^7^, ^8^] Heterocyclic structures are found in various natural and synthetic compounds.[^9^, ^10^, ^11^] It has been estimated that more than 85 % of all chemical entities that exhibit biological activity contain at least one heterocycle.[^12^, ^13^, ^14^] The introduction of heterocycles into drug molecules allows for the modification of pharmacokinetic and pharmacodynamic properties by altering parameters such as polarity, lipophilicity, hydrogen bonding ability, and toxicological profiles.[^15^, ^16^, ^17^] 2,4‐Thiazolidinedione is a crucial heterocyclic compound in the design and development of new medicinal agents.[^18^, ^19^] It is a five‐membered structure with the formula C_3_H_3_NO_2_S containing carbonyl groups at positions 2 and 4, which act as electron acceptors, along with NH and S serving as electron donors, and it potently interacts with important biological species like DNA, receptors, and enzymes.[^20^, ^21^, ^22^] In thiazolidinediones, a large number of substitutions are possible at positions 2, 4, and 5, enhancing the pharmaceutical importance of the compounds.^[23]^ TZD exists in the form of a white crystalline solid with a melting point of 123–125 °C and remains stable when kept below 30 °C.^[24]^ TZD is sparingly soluble in common organic solvents such as water, methanol, and ethanol.^[25]^ TZD can exist as a series of tautomer's due to the presence of two carbonyl groups and one α‐hydrogen. It is a highly utilized scaffold for the design and development of pharmaceutically active compounds.^[26]^ TZD has been shown to exhibit biological activity towards a wide variety of targets and is present in numerous biological compounds, including antimicrobial, antimalarial, anti‐inflammatory, antidiabetic, anticancer, antioxidant, and antitubercular agents.[^27^, ^28^] Researchers focus on this moiety as it is associated with the control of various physiological activities. Type 2 diabetes mellitus is the major form of diabetes resulting from defects in insulin secretion with a major contribution from insulin resistance.[^29^, ^30^] TZDs increase tissue sensitivity towards insulin and enhance glucose uptake by activating PPAR‐γ, which regulates gene transcription.^[31]^ Investigators are also exploring other targets such as PTP‐1B and ALR2 for thiazolidinediones.^[32]^ PTP‐1B (Protein Tyrosine Phosphatase) plays a significant role in diseases like diabetes and obesity.^[33]^ It inhibits leptin and insulin binding, thereby reducing fatty acid oxidation and glucose uptake and metabolism, which can lead to diabetes.^[34]^ Inhibiting PTP‐1B may increase glucose uptake and metabolism, potentially reducing the risk of diabetes and obesity.^[35]^ ALR2 (Aldose Reductase) is also crucial in diabetes management. It converts glucose to sorbitol, which is further metabolized to fructose, generating Reactive Oxygen Species (ROS) that cause cellular damage and diabetic complications.^[36]^ ALR2 inhibitors can manage diabetes by blocking the initial step in the sorbitol (polyol) pathway.^[37]^ TZDs also have certain long‐term adverse effects that depend on the dose and duration of use, limiting their application.^[38]^ They cause weight gain by increasing body fat as part of their mechanism of action.^[39]^ A significant drawback of TZDs, particularly derivatives like rosiglitazone and pioglitazone, is the increased risk of congestive heart failure due to elevated triglyceride levels.^[40]^ Additionally, TZDs contribute to loss of bone density, leading to osteoporosis and fractures.^[41]^ Other limitations associated with TZDs include hepatotoxicity, oedema, fluid retention, and the potential for bladder cancer.^[42]^ Despite side effects associated with TZDs, they can be discovered as antidiabetics, including alpha‐amylase inhibitors, and may reduce post‐meal glucose levels by slowing down the conversion of starch to monosaccharides.[^43^, ^44^] TZDs interfere with the expression of a wide range of proteins with pro‐inflammatory properties, such as cyclooxygenase‐2 (COX‐2), inducible nitric oxide synthase (iNOS), and various cytokines, but the molecular mechanisms responsible for these activities have not been clarified yet.^[45]^ Most TZDs exhibit good bactericidal activity against a variety of gram‐positive and gram‐negative bacteria and have also shown significant antifungal activity.^[46]^ TZDs have been reported as an effective scaffold playing a vital role in cancer therapy, displaying anticancer activity in a wide range of experimental cancer models by influencing the cell cycle, inducing cell differentiation, and inhibiting tumor angiogenesis.[^47^, ^48^]

## 2 Marketed Compounds

**Figure 1:**
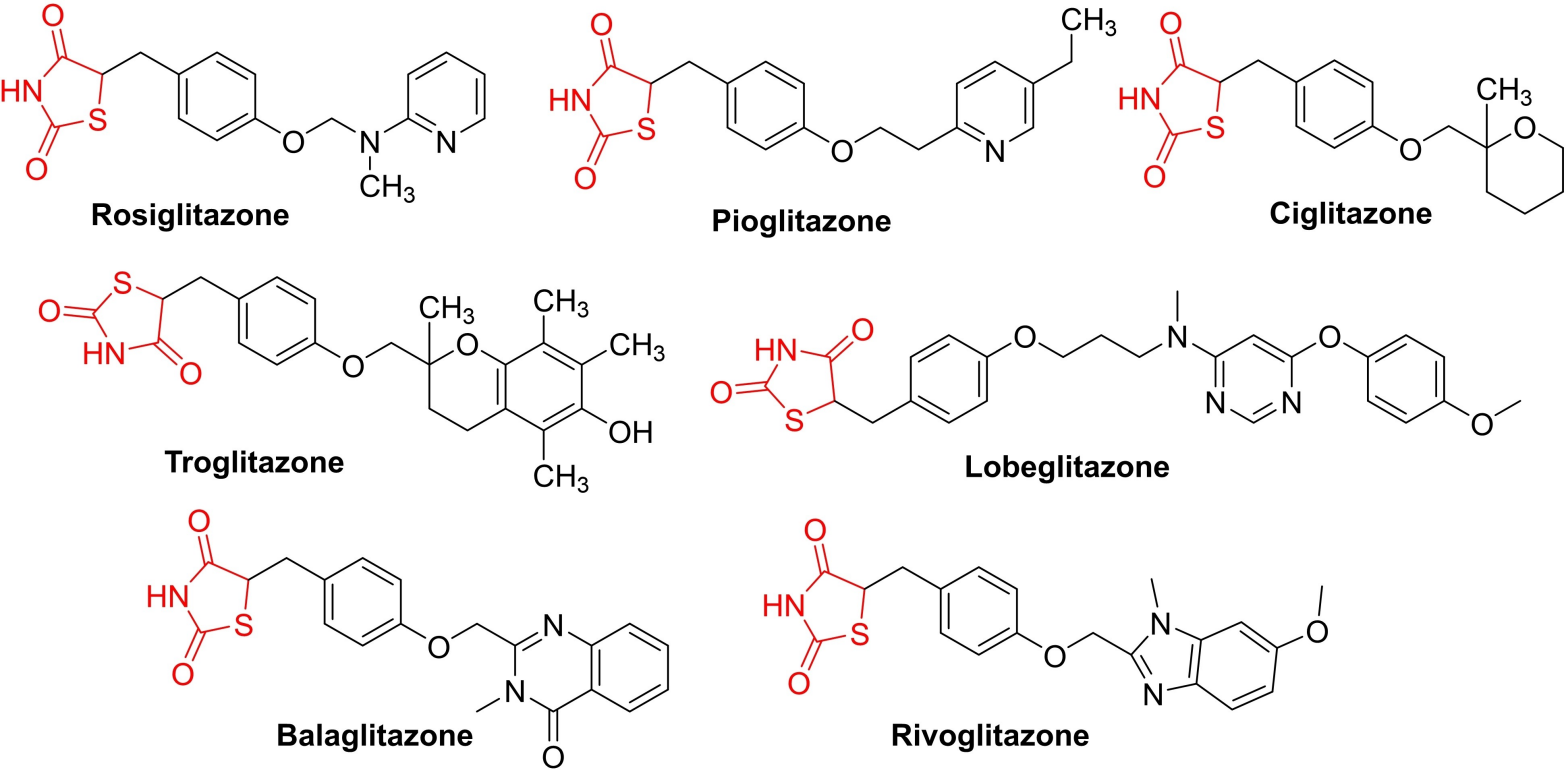
Clinical and investigational compounds currently available.

Rosiglitazone, a derivative of 2,4‐thiazolidinedione, is an FDA‐approved drug for treating type 2 diabetes mellitus. However, its use is linked to risks of congestive heart failure and myocardial infarction.[^49^, ^50^] Pioglitazone, another member of this class, effectively lowers elevated blood glucose levels by reducing insulin resistance and is commonly prescribed alone or in combination with other drugs like metformin.^[51]^ Ciglitazone, developed by Takeda Pharmaceutical in the 1980s, is a thiazolidinedione derivative that never saw clinical use.^[52]^ Troglitazone, the first marketed 2,4‐thiazolidinedione antidiabetic agent introduced in 1997, was swiftly withdrawn due to fatal idiosyncratic hepatotoxicity.^[53]^ Lobeglitazone, also belonging to the TZD class, is utilized for managing type 2 diabetes mellitus but lacks approval from the USA FDA, Health Canada, and the European Medicine Agency (Figure 1).^[54]^

Balaglitazone, a novel partial agonist of PPAR‐γ, developed by Dr. Reddy's Laboratories as an antihyperglycemic agent, is currently in Phase III clinical trials in the United States and Europe.^[55]^ Rivoglitazone, another thiazolidinedione derivative by Daiichi Sankyo, is under research for potential use in treating type 2 diabetes mellitus. These drugs hold promise for further evolution and evaluation of their therapeutic potential.^[56]^

## 3 Mechanism of Action

**Figure 2:**
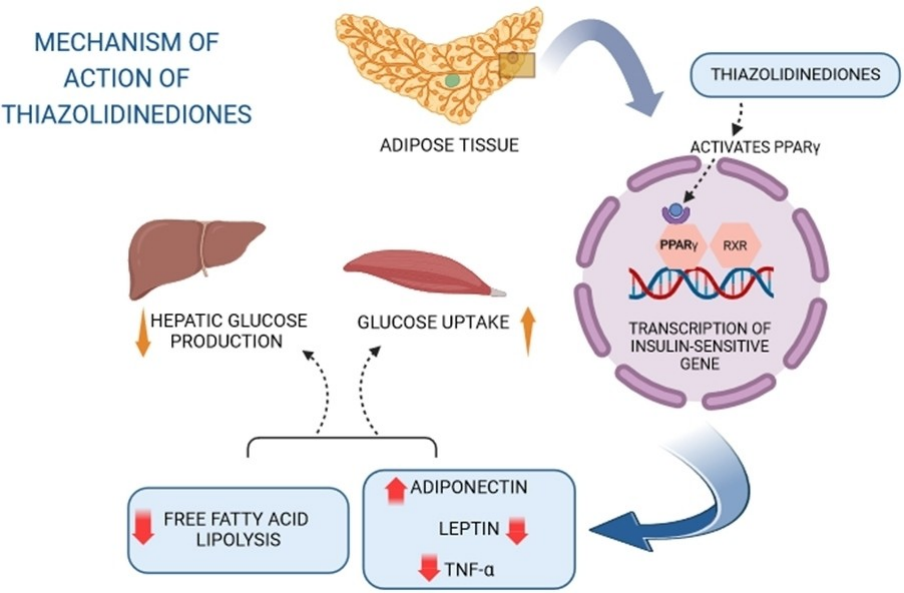
Mechanism of action of 2,4‐thiazolidinediones.

Thiazolidinediones primarily function as insulin sensitizers, operating within cellular metabolic pathways to enhance insulin secretion and decrease insulin resistance.^[57]^ They achieve this by activating the nuclear receptor PPAR‐γ (Peroxisome Proliferator‐Activated Receptor Gamma), pivotal in glucose and lipid metabolism. By binding to PPAR‐γ, TZDs initiate gene transcription of insulin‐sensitive genes, leading to three main responses that contribute to their antidiabetic effects (Figure 2).[^58^, ^59^] They reduce insulin resistance, consequently elevating insulin levels and lowering blood glucose.[^60^, ^61^] Additionally, TZDs increase the expression of GLUT‐1 and GLUT‐2 receptors, facilitating glucose uptake into cells and further reducing blood glucose levels.^[62]^ Furthermore, they mitigate hepatic gluconeogenesis, curtailing glucose production. Moreover, TZDs exhibit anti‐inflammatory properties by suppressing the production of pro‐inflammatory cytokines.^[63]^

**Figure 3:**
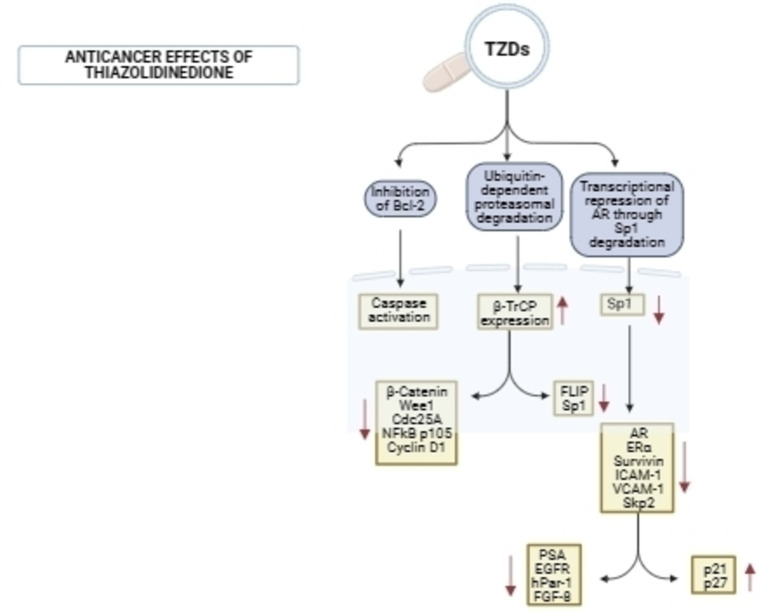
Anticancer mechanism of action of 2,4‐thiazolidinediones.

Thiazolidinediones exhibit anticancer effects by disrupting the cell cycle, cell proliferation, cell differentiation, and apoptosis in cancer cells.^[64]^ They are implicated in inhibiting the function of Bcl‐2/Bcl‐xL, which activates caspases, ultimately inducing apoptosis.^[65]^ Additionally, TZDs contribute to the proteasomal degradation of specific proteins and transcriptional repression of AR through Sp1 degradation, thereby inhibiting gene expression and impeding cell growth, which prevents cancer progression (Figure 3).^[66]^

TZD derivatives exhibit antimicrobial activity against Gram‐positive and Gram‐negative bacteria by inhibiting key bacterial enzymes like DNA gyrase and topoisomerase IV, and interfering with cell wall synthesis. Electronegative groups on the TZD scaffold enhance antibacterial potency. They also demonstrate antifungal properties by disrupting fungal cell membrane integrity and inhibiting ergosterol synthesis through binding to cytochrome P450 enzymes. Additionally, some TZD derivatives show antiviral activity against RNA viruses by inhibiting RNA‐dependent RNA polymerase and interfering with viral entry and fusion, thereby blocking viral replication.[^65^, ^66^]

## 4 Recent Advances

### 4.1 Antidiabetic Activity

**Figure 4:**
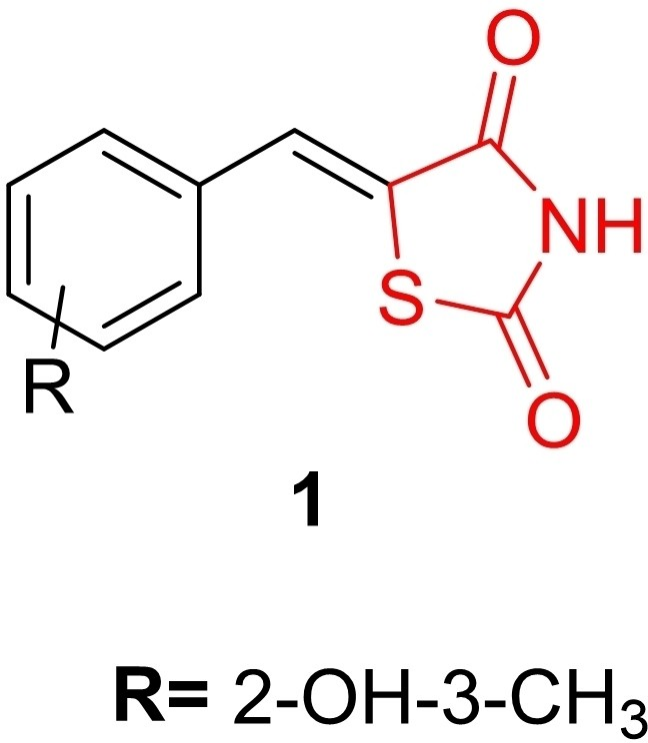
5‐(arylidene)‐appended 2,4‐thiazolidinedione based potent antidiabetic compounds.

Sever *et al*., reported on the design and synthesis of 5‐(arylidene) thiazolidine‐2,4‐diones through a solvent‐free reaction involving 2,4‐thiazolidinedione with aromatic aldehydes in the presence of urea. The synthesized compounds underwent investigation for potent aldose reductase inhibitory activity and cytotoxicity. Notably, Compound (**1**, Figure 4) emerged as the most potent aldose reductase inhibitor among all the synthesized derivatives, displaying a ki value of 0.445 μM and an IC_50_ value of 0.382 μM. In addition, researchers conducted the MTT assay to determine cytotoxic activity in L929 mouse fibroblast cells, revealing that Compound **1** exhibited low cytotoxicity with an IC_50_ value of 8.9 μM, indicating its safety. Molecular docking studies confirmed the strong binding affinity of Compound **1** to the aldose reductase binding site. Further in silico analysis suggested that Compound **1** possesses favorable pharmacokinetic properties. In conclusion, the researchers highlighted that, considering both in silico and *in vitro* data, Compound **1** emerges as a potential orally bioavailable aldose reductase inhibitor suitable for managing diabetic complications and other diseases.^[67]^

**Figure 5:**
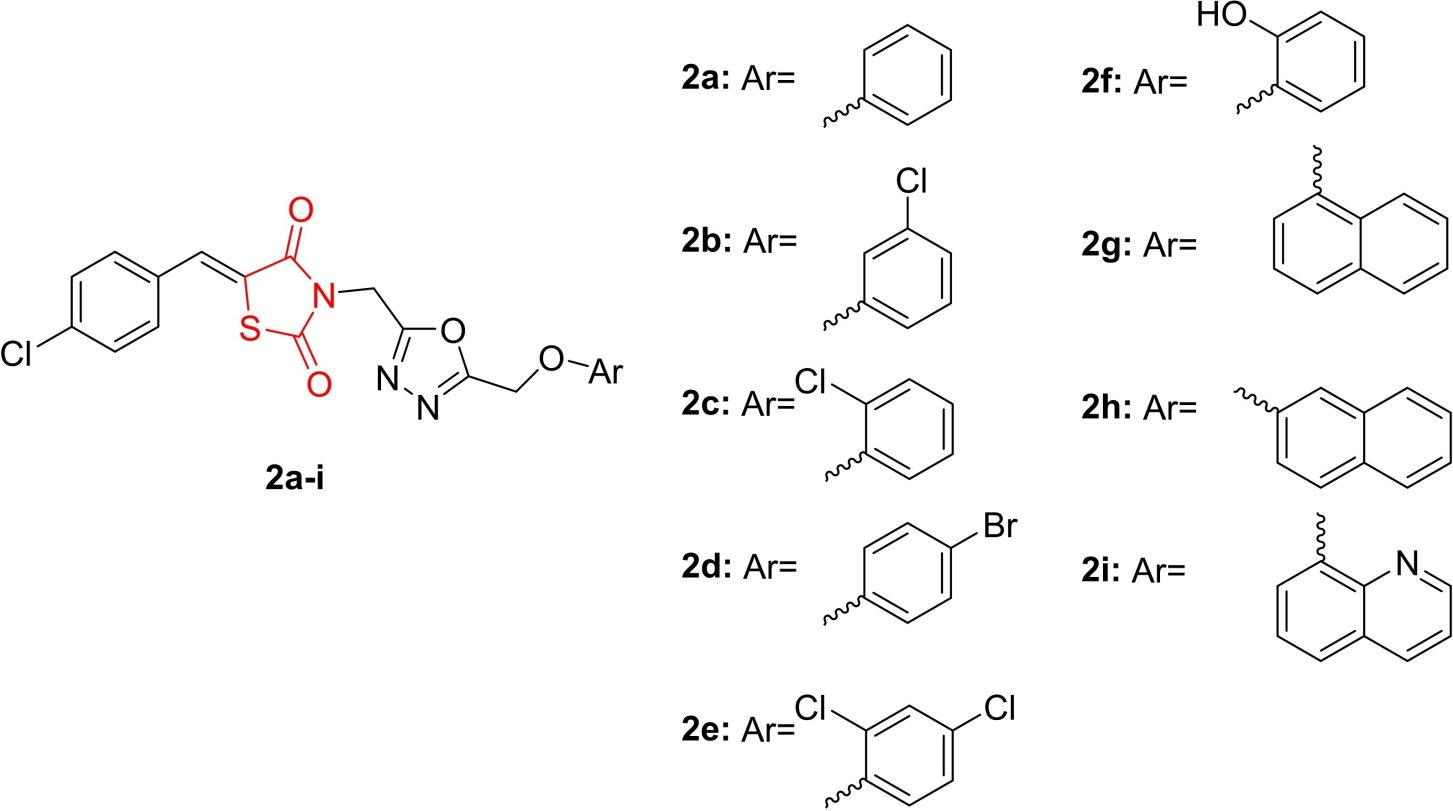
2,4‐thiazolidinedione hybrids as potent antidiabetic agents.

Nazreen *et al*., reported the design and synthesis of new thiazolidine‐2,4‐dione hybrids (**2 a**–**2 i**, Figure 5). The synthesized derivatives underwent investigation for their potential as peroxisome proliferator‐activated receptor (PPAR)‐γ agonists and thymidylate synthase inhibitory activity. The pharmacokinetic properties of all newly synthesized derivatives were analyzed, confirming adherence to Lipinski's and Veber's rules. Among the compounds, **2 g** and **2 h** demonstrated notable potency, with PPAR‐γ trans‐activation values of 73.4 % and 78.9 %, respectively. These compounds also increased PPAR‐γ gene expression by 2.4 and 2.2‐fold, respectively.

In cytotoxicity studies, compounds **2 h**, **2 g**, and **2 d** exhibited promising results, with IC_50_ values ranging from 1.4–4.5 μM against MCF‐7 cells and from 1.8‐8.4 mM against HCT‐116 cells. Analysis of thymidylate synthase inhibitory activity revealed that compounds **2 g** and **2 h** inhibited thymidylate synthase with IC_50_ values of 5.1 and 3.2 μM, respectively, confirming their mode of action as thymidylate synthase inhibitors. Molecular docking studies further supported these findings, indicating that compounds **2 g** and **2 h** exhibited the highest binding affinity (−7.3 and −7.2 kcal/mol). In conclusion, researchers suggested that compounds **2 g** and **2 h** hold promise for future investigations as PPAR‐γ agonists and thymidylate synthase inhibitors.^[68]^

**Figure 6:**
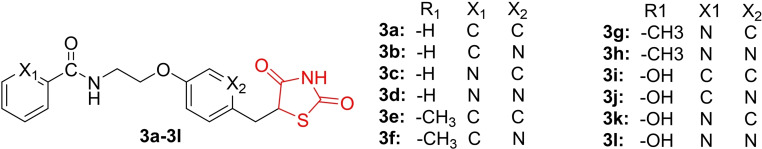
Rosiglitazone based potent antidiabetic compounds.

Huiying *et al*., reported the design and synthesis of a new class of 2,4‐thiazolidinedione compounds using rosiglitazone as the lead compound and applying the bioisostere principle (**3 a**–**3 l**, Figure 6). The synthesized derivatives underwent investigation for their insulin‐enhancing or PPAR‐γ (peroxisome proliferator‐activated receptor‐γ) activation activity. Through structure‐activity relationship (SAR) studies, researchers identified that compounds **3 a**, **3 e**, **3 f**, **3 g**, and **3 i** exhibited strong PPAR‐γ activation properties. *In vitro* tests confirmed that compound **3 e** was the most potent among all, with an EC_50_ value of 0.03 μmol/L compared to rosiglitazone (EC_50=_0.08 μmol/L). Subsequent *in vivo* tests, including glucose tolerance test, insulin tolerance test, cell survival experiments, and acute toxicity assessments, were conducted on the potent compounds (**3 a**, **3 e**, **3 f**, **3 g**, and **3 i**). The results indicated a significant reduction in blood sugar levels and enhanced exogenous insulin hypoglycemic effects, using rosiglitazone as a positive reference and DMSO as a blank control. Cytotoxicity and acute toxicity tests demonstrated low toxicity for these compounds, suggesting their suitability for oral administration. In conclusion, researchers highlighted compound **3 e** as a potential candidate for further investigation as a PPAR‐γ activator or insulin enhancer.^[69]^

**Figure 7:**
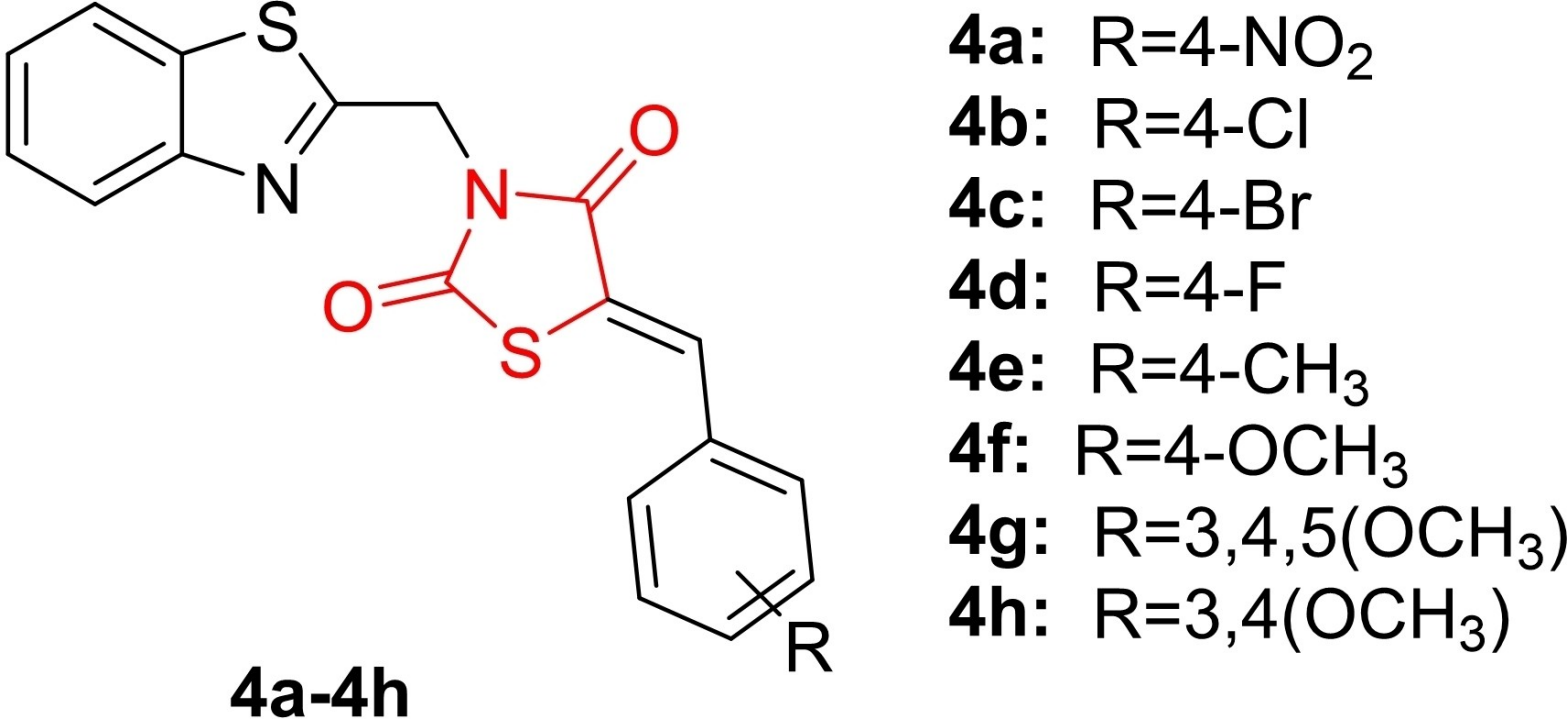
Benzothiazole and nitrophenacyl clubbed thiazolidine‐2,4‐dione based antidiabetic compounds.

Hamdi *et al*., reported the design and synthesis of two series of new thiazolidine‐2,4‐dione hybrids (**4 a**–**4 h**, Figure 7) by combining benzothiazole heterocycle and nitrophenacyl structures. The synthesized derivatives underwent investigation for their aldose reductase (AR) inhibitory and antihyperglycemic activity. *In vitro* tests revealed that compound **4 b** exhibited the highest potency in inhibiting AR in a non‐competitive manner, with an IC_50_ value of 0.16 μM compared to epalrestat (IC_50_=0.10).

*In vivo* experiments on mice with streptozotocin‐induced hyperglycemia demonstrated significant reductions in blood glucose levels with a 50 μg/kg dose of **4 b**, and noticeable antihyperglycemic effects were observed with a 5 μg/kg dose. In silico calculations indicated favorable drug‐like properties and pharmacokinetics for these compounds as non‐competitive AR inhibitors. Molecular docking studies confirmed the binding of hybrid **4 b** to the AR active site. In conclusion, the researchers highlighted that this hybrid, **4 b**, showed promise for further development into more effective and selective AR inhibitors for managing diabetic complications.^[70]^

**Figure 8:**
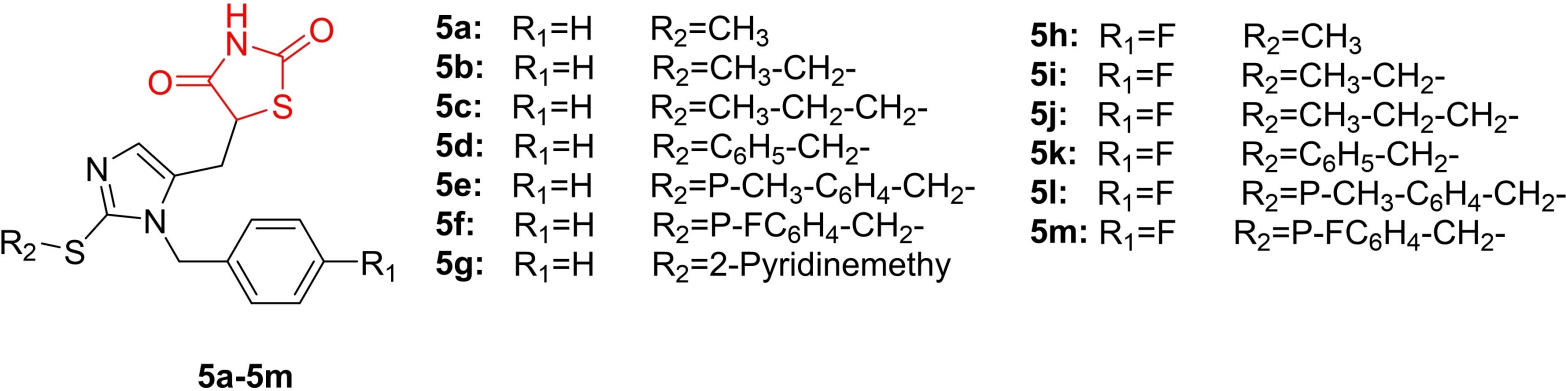
Imidazolyl‐methyl‐1 based 2,4‐thiazolidinediones as antidiabetic agents.

Shakour *et al*., reported the design and synthesis of a series of imidazolyl‐methyl‐1‐2,4‐thiazolidinediones (**5 a**–**5 m**, Figure 8) and investigated their anti‐diabetic activity. The synthesized derivatives exhibited non‐toxicity to normal NIH/3T3 cells, with viability above 82 %. In an *in vivo* animal study, compounds **5 e** and **5 b** (11×10^−6^ mol/kg) demonstrated more effective reduction of blood glucose compared to pioglitazone at the same dose. To assess toxicity, researchers identified compounds **5 g** and **5 e** as having the lowest toxicity (less than 0.3) and no cardiotoxicity. Compound **5 e**, chosen as the preferred candidate, exhibited no liver and pancreas toxicity, along with an oral bioavailability comparable to the standard drug pioglitazone. Glucose consumption assay results indicated that compound **5 e** significantly lowered glucose levels (p<0.001) in HepG2 cells exposed to 11 mM of glucose at concentrations ranging from 1.25 to 10 mm of compound **5 e**. In the PPAR‐γ gene expression study, compounds **5 e** and pioglitazone reduced PPAR‐γ gene expression, with **5 e** showing a decrease of 0.09‐fold and pioglitazone decreasing it by 0.41‐fold relative to the control group. In conclusion, researchers suggested that compound **5 e** holds promise as a candidate for further investigation as an anti‐diabetic agent.^[71]^

**Figure 9:**
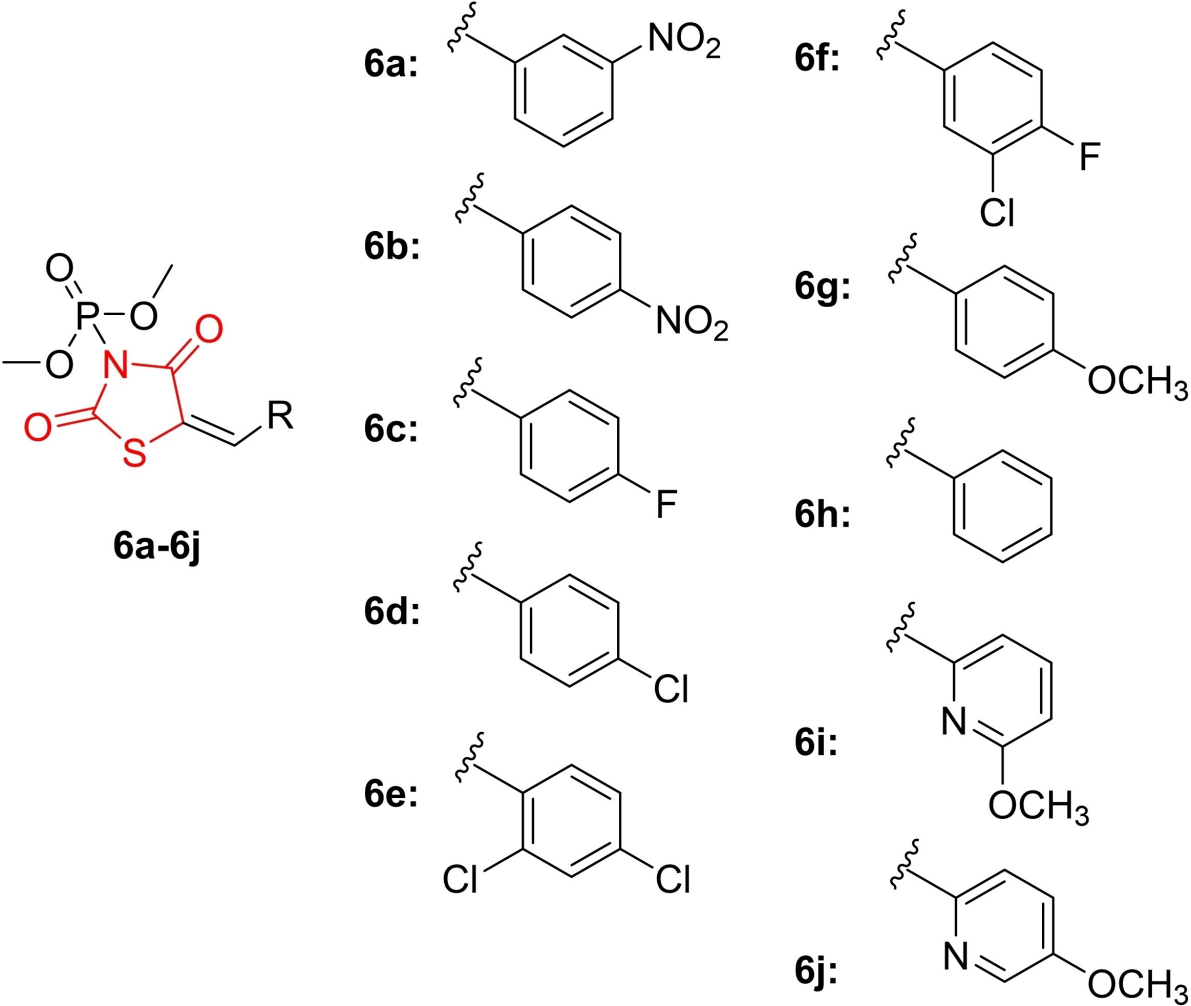
Phosphate‐appended 2,4‐thiazolidinediones as antidiabetic agents.

Sujatha *et al*., reported the design and synthesis of thiazolidinedione derivatives containing a phosphate moiety (**6 a**–**6 j**, Figure 9). The structures of all synthesized compounds were confirmed using NMR spectroscopy, IR spectroscopy, mass spectrometry, and elemental analysis for C, H, N. The anti‐diabetic activity of the synthesized compounds was investigated. Molecular docking studies revealed that compounds **6 a**, **6 f**, **6 e**, and **6 j** were particularly potent, exhibiting superior binding energies (−7.8, −7.6, −7.5, and −7.6 kcal/mol) with the target gene PPAR‐γ compared to the reference drug rosiglitazone (−7.4 kcal/mol).

*In vitro* anti‐diabetic activity was assessed through alpha‐amylase inhibition assays, where most derivatives, especially **6 a**, **6 f**, **6 e**, and **6 j**, demonstrated significant alpha‐amylase inhibition with percent inhibition values of 43.9, 41.4, 40.9, and 44.3 %, respectively, at a concentration of 50 mg/ml, compared to the standard drug acarbose (47.8 %). In conclusion, researchers suggested that compounds **6 a**, **6 e**, **6 f**, and **6 j** hold promise as potential candidates for future investigation as anti‐diabetic agents.^[72]^

**Figure 10:**
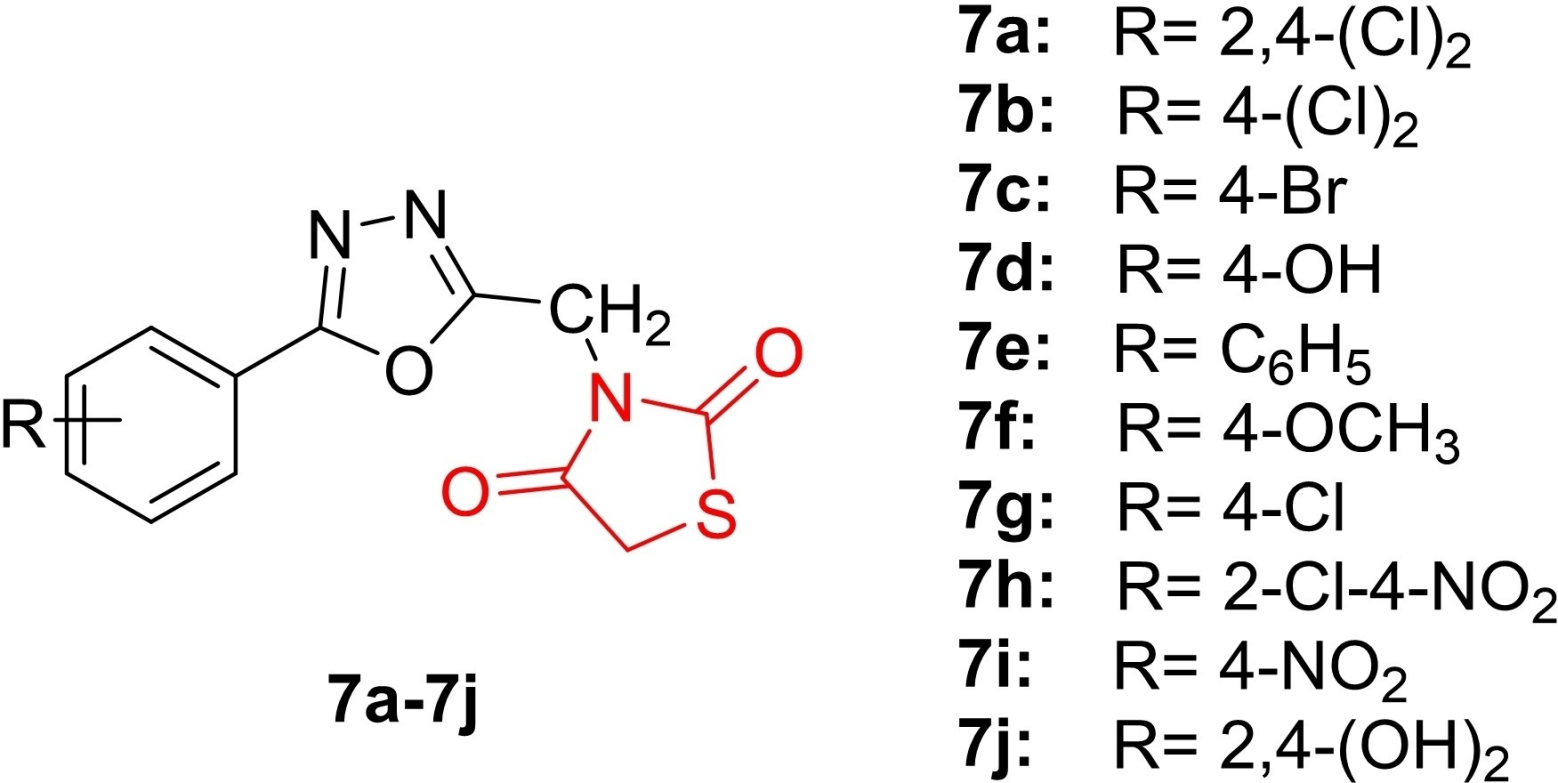
Oxadiazol clubbed thiazolidine‐2,5‐dione based antidiabetic compounds.

Srinivasa *et al*., reported the design and synthesis of a series of novel 3‐((5‐phenyl‐1,3,4‐oxadiazol‐2‐yl)methyl)thiazolidine‐2,5‐dione derivatives (**7 a**–**7 j**, Figure 10) and characterized their chemical structure using FTIR, H‐NMR, C‐NMR, and MS techniques. The synthesized derivatives were investigated for their anti‐diabetic activity. In molecular docking studies, researchers revealed that compounds **7 a** and **7 j** exhibited the highest binding energies (−6.217 and −6.56 kcal/mol) at the active site of α‐amylase and α‐glucosidase. In the *in vitro* anti‐diabetic assay, compounds **7 a** and **7 j** emerged as the most potent inhibitors, with IC_50_ values of 18.61 and 18.42 mM against α‐amylase and 17.58–17.21 mM against α‐glucosidase, surpassing acarbose (IC50=24.35 and 23.73 mM). The *in vivo* analysis, conducted using the Drosophila melanogaster fly model, demonstrated that compounds **7 a** and **7 j** exhibited potent antidiabetic activity compared to acarbose. Additionally, these compounds displayed favourable pharmacokinetic and drug‐likeness properties. In conclusion, researchers suggested that compounds **7 a** and **7 j** warrant further evaluation for their anti‐diabetic potential.^[73]^

**Figure 11:**
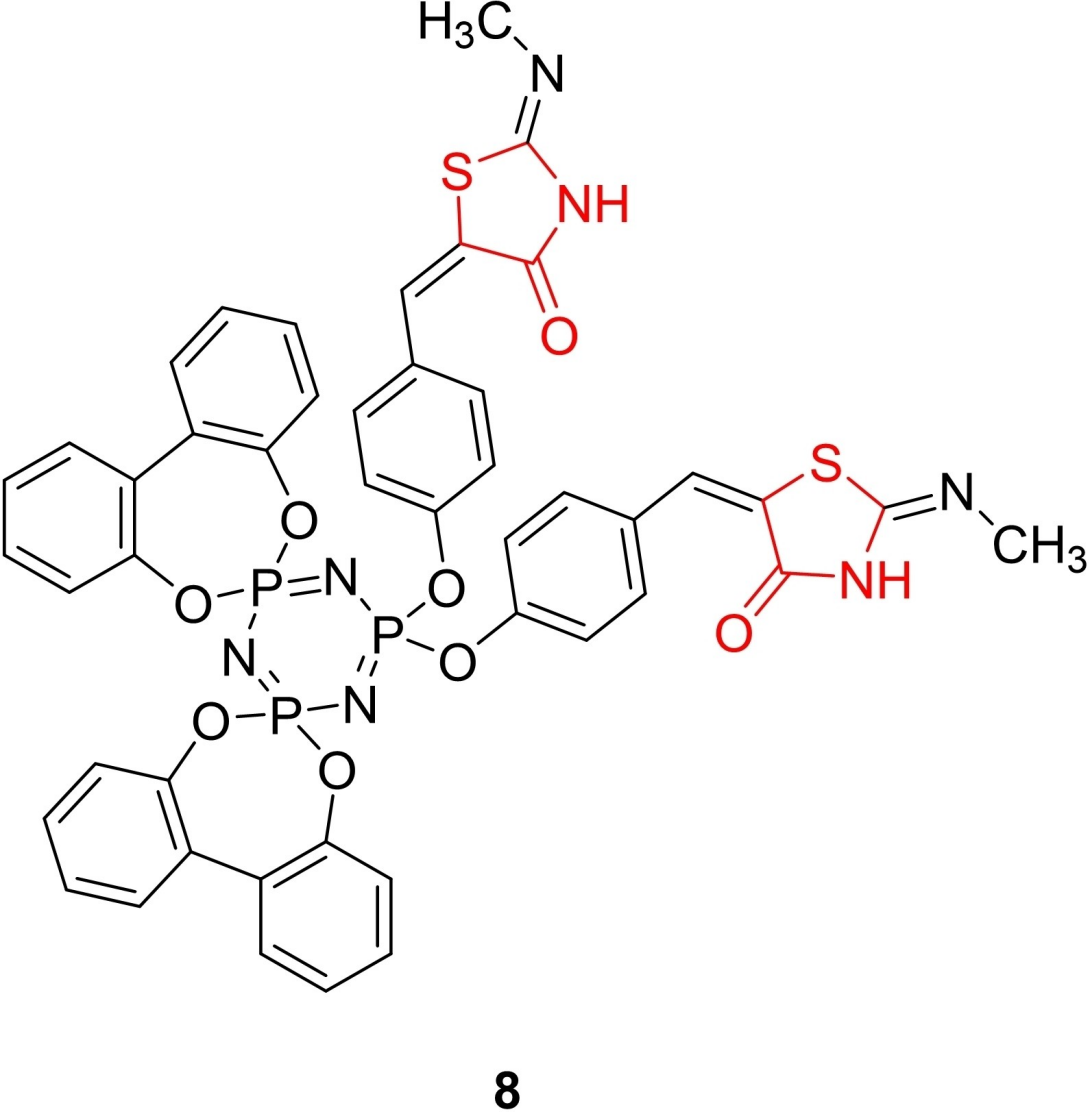
Phosphazene containing thiazolidinedione derivative as antidiabetic agents.

Neyadi *et al*., reported the design and synthesis of a novel phosphazene derivative containing a thiazolidinedione group (**8**, Figure 11). This derivative was synthesized through the reaction of 2,2‐bis(4‐formalphenoxy)‐4,4,6,6‐bis[spiro(2’,2”‐dioxy‐1’,1”‐biphenyl)yl]cyclotriphosphazene with N‐methyl thiourea and monochloroacetic acid using microwave irradiation. The chemical structure of the synthesized derivative was characterized using FT‐IR, H‐NMR, C‐NMR, P‐NMR, and elemental analysis.

The researchers explored the antidiabetic activity of the derivative as a peroxisome proliferator‐activated receptor‐gamma (PPAR‐γ) agonist. Insulin secretion assays from the βTC6 cell line indicated that compound **8** did not exhibit any significant effect on insulin secretion in the presence or absence of glucose when compared to the reference drug pioglitazone. However, in the *in vitro* glucose uptake study, compound **8** demonstrated potent activity by significantly enhancing glucose uptake (P<0.05) compared to pioglitazone. In conclusion, the researchers suggested that compound **8** holds promise for further modifications and evaluations to explore its potential as an insulin sensitizer for antidiabetic applications.^[74]^

**Figure 12:**
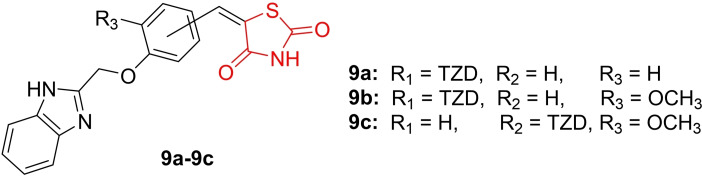
Benzimidazole containing thiazolidinedione derivative as antidiabetic agents.

Hernandez *et al*., conducted a study on the design and synthesis of benzimidazole‐thiazolidinedione hybrids (**9 a**–**9 c**, Figure 12), characterizing their chemical structure through spectroscopic analyses such as H‐NMR and C‐NMR, as well as spectrometric methods. The synthesized derivatives were explored for their antihyperglycemic activity. *In vitro* treatments on adipocytes were performed to evaluate mRNA expression by qPCR. Results indicated that compounds **9 b** and **9 c** significantly increased the mRNA expression of key diabetes‐related proteins, PPAR‐γ (approximately 2.7‐ to 3.2‐fold), and GLUT‐4 (3.5‐fold), in comparison to pioglitazone. To further assess their efficacy, *in vivo* oral glucose tolerance tests (OGTT) were conducted, revealing that compounds **9 a**, **9 b**, and **9 c** exhibited antihyperglycemic effects, suggesting an insulin sensitization mechanism induced by PPAR agonism. Molecular docking studies were also carried out, demonstrating that compounds **9 a**, **9 b**, and **9 c** exhibited excellent binding affinity towards the active binding site of PPAR‐γ, with binding energies of −7.98, −8.17, and −8.96 kcal/mol, respectively. In conclusion, the researchers proposed that compounds **9 a**–**9 c** warrant further evaluation for their potential antihyperglycemic effects.^[75]^

**Figure 13:**
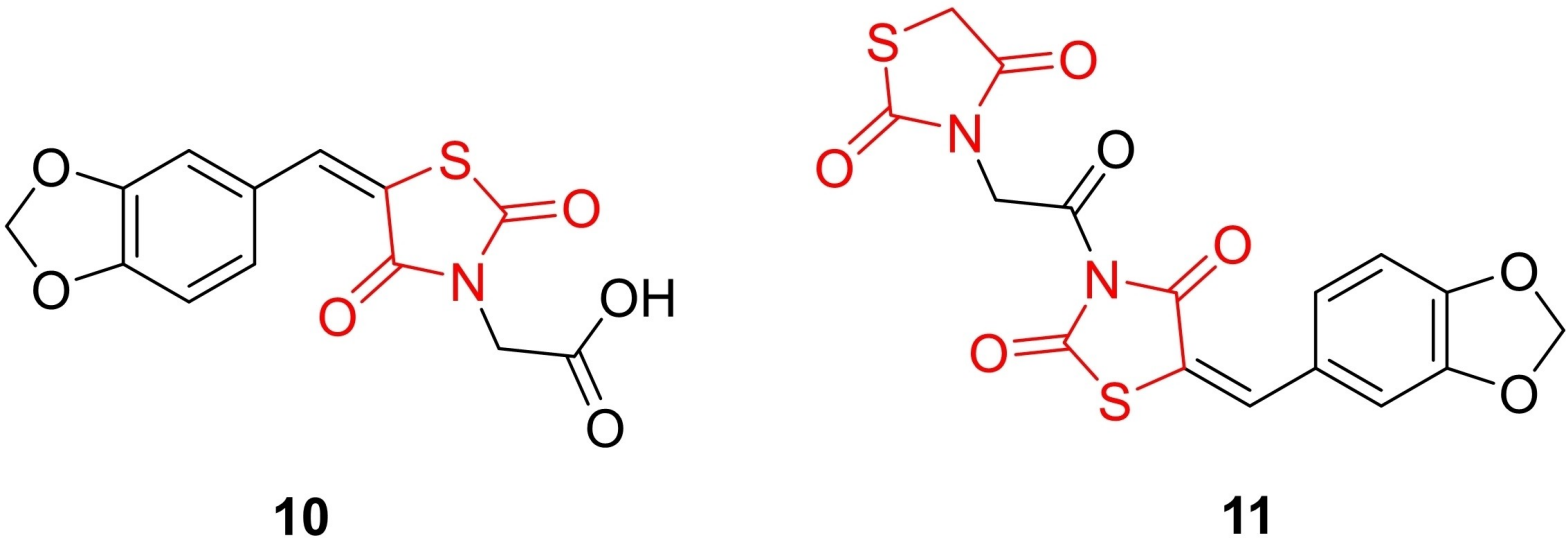
Potent thiazolidinedione derivatives as antihyperglycemic agents.

Sameeh *et al*., conducted a study on the design and synthesis of potent thiazolidinedione derivatives (**10** and **11**, Figure 13), characterizing them through spectral data analysis. The synthesized derivatives were evaluated for their antihyperglycemic activity. The researchers employed Density Functional Theory (DFT) to discuss frontier molecular orbitals (FMOs), chemical reactivity, and molecular electrostatic potential (MEP) of the compounds, aiming to elucidate the interaction between the derivatives and biological receptors. *In vitro* α‐amylase inhibition assays were performed, revealing that compound **10** exhibited significant inhibitory activity with an IC_50_ value of 10.26 mg/ml, compared to acarbose (IC_50_=24.1 mg/ml). The researchers also assessed the *in vitro* antioxidant activity, finding that compound **10** displayed the most potency with an IC_50_ value of 10.78 mg/ml, relative to ascorbic acid. In an *in vivo* setting using an alloxan‐induced diabetic rat model, researchers examined the antidiabetic and anti‐hyperlipidemic activity. Compounds **10** and **11** showed the most potent antihyperglycemic effects, reducing blood glucose levels by 69.55 % and 66.95 %, respectively. All compounds maintained normal values for tested biochemical parameters (CH, LDL, and HDL), indicating potential anti‐hyperlipidemic effects. Molecular docking studies demonstrated that compounds **10** and **11** exhibited good binding affinity towards PPAR‐γ and α‐amylase.

The researchers highlighted that these potent derivatives demonstrated favorable oral bioavailability, drug‐likeness, and pharmacokinetic properties without inducing carcinogenic effects. In conclusion, compounds **10** and **11** were suggested for further evaluation due to their promising antidiabetic potential.^[76]^

**Figure 14:**
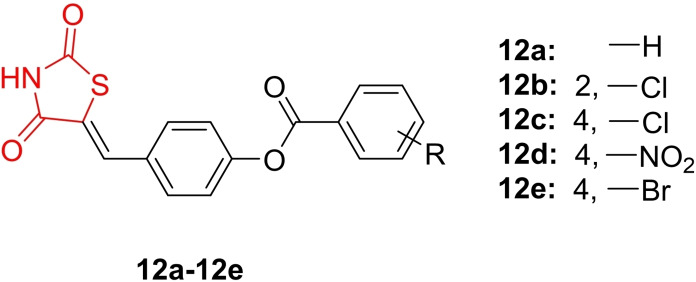
Conformationally restricted thiazolidine‐2,4‐dione as antihyperglycemic agents.

Chhajed *et al*., reported the de novo design and synthesis of conformationally restricted thiazolidine‐2,4‐dione derivatives (**12 a**–**12 e**, Figure 14), elucidating their chemical structure through FT‐IR, H‐NMR, and mass analysis. The synthesized derivatives were explored for their anti‐diabetic activity as PPAR‐γ (peroxisome proliferator‐activated receptor gamma) agonists. To assess toxicity, the researchers conducted tests against 3T3‐L1 cell lines. Compounds **12 a**, **12 b**, **12 c**, and **12 d** displayed IC_50_ values of 150.2, 100.7, 275.6, and 242.52 μM, respectively, in comparison to pioglitazone (IC_50_=148.3 μM). In the glucose uptake assay, **12 d** demonstrated the highest potency, with cells absorbing 2.6 mmol/L of glucose, slightly surpassing pioglitazone (2.5 mmol/L). Molecular docking studies were performed, revealing that **12 d** exhibited excellent binding affinity (−8.2 kcal/mol) toward the active site of PPAR‐γ. Additionally, it displayed hydrogen bond interactions with amino acids Glu259, Ile281, Gly284, Cys285, and Leu340, along with pi‐interactions with Glu259 and Ile341. In conclusion, the researchers suggested that compound **12 d** warrants further evaluation for its anti‐diabetic potential as a PPAR‐γ agonist.^[77]^

**Figure 15:**
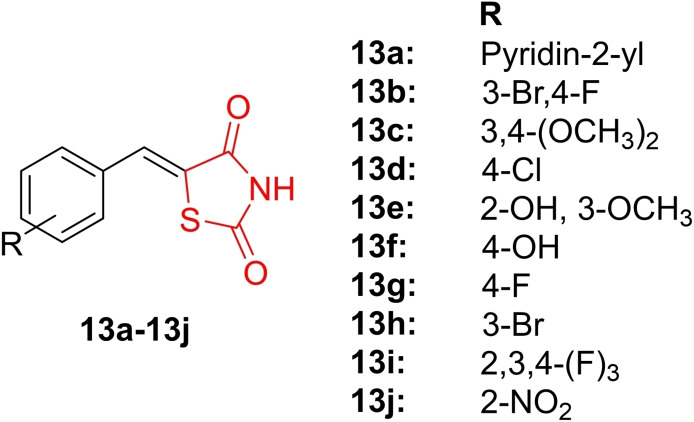
C‐5 substituted thiazolidine‐2,4‐dione based antihyperglycemic compounds.

Pardeshi *et al*., reported the design and synthesis of C‐5 substituted thiazolidine‐2,4‐dione analogs (**13 a**–**13 j**, Figure 15), confirming their chemical structure through IR, H‐NMR, and Mass spectroscopy. The synthesized derivatives underwent evaluation for their antidiabetic activity. Conducting an acute oral toxicity test on the derivatives, researchers observed no toxic effects even at a dosage of 2000 mg/kg. The antidiabetic activity of compounds **13 a**, **13 b**, and **13 i** was determined on streptozotocin‐induced diabetic Swiss albino mice. These derivatives effectively reduced blood sugar levels to 133, 124, and 125 mg/dl after 8 days of treatment, in comparison to pioglitazone (127 mg/dl). Molecular docking studies revealed that compounds **13 a**, **13 b**, and **13 i** exhibited excellent binding to the active site of PPAR‐γ, with binding energies of −7.2, −6.8, and −6.4 kcal/mol, respectively. These values were comparable to rosiglitazone (−7.5 kcal/mol) and pioglitazone (−7.6 kcal/mol). Additionally, the derivatives displayed amino acid interactions similar to rosiglitazone. In conclusion, researchers suggested that compounds **13 a**, **13 b**, and **13 i** warrant further evaluation for their antidiabetic potential as PPAR‐γ agonists.^[78]^

**Figure 16:**
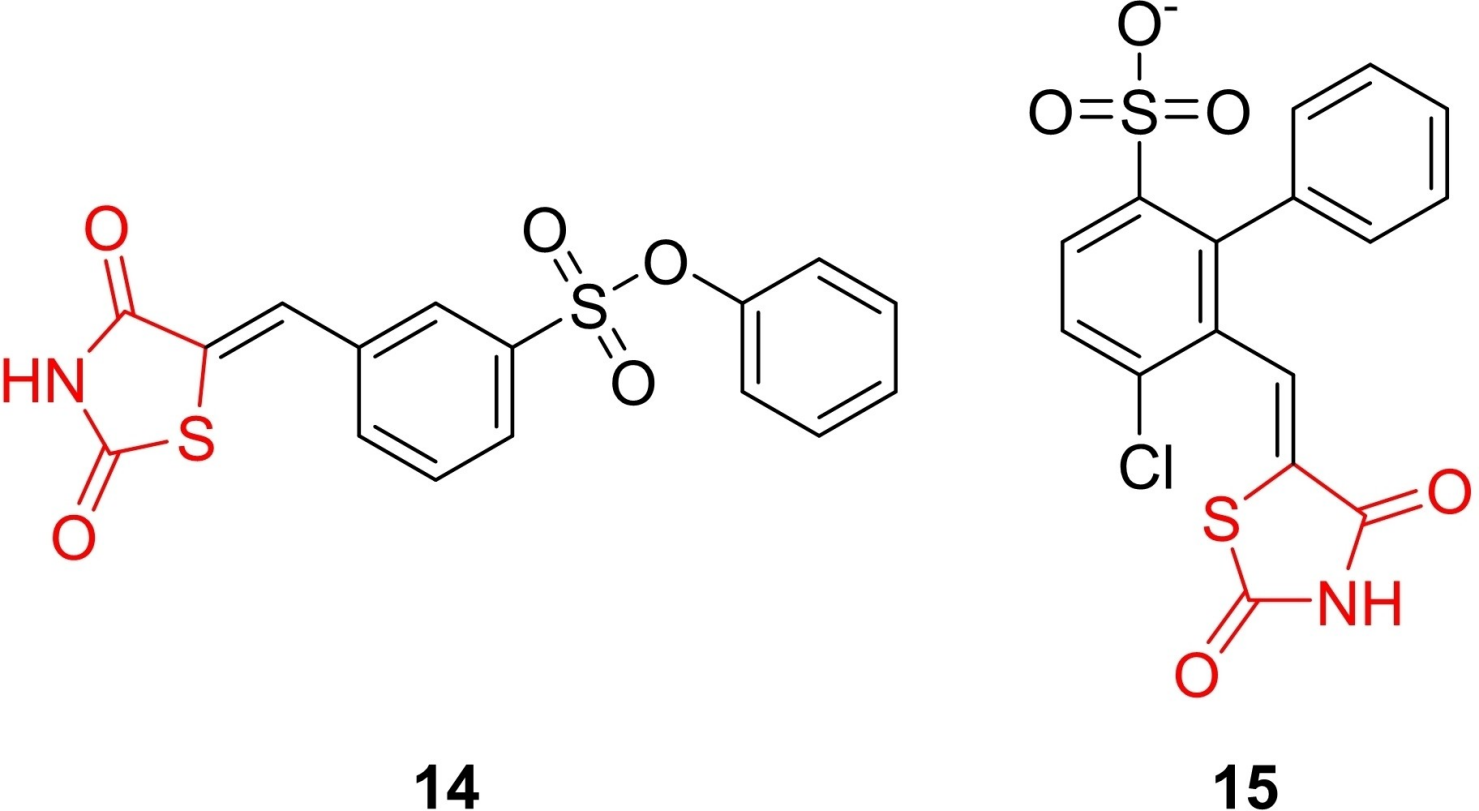
5‐arylidene‐thiazolidine‐2,4‐dione based oral‐hypoglycaemic agents.

Mehta *et al*., reported the design and synthesis of aryl sulfonate ester‐conjugated 5‐arylidene‐thiazolidine‐2,4‐dione derivatives (**14** and **15**, Figure 16), with the chemical structures characterized using H‐NMR, C‐NMR, and MS spectroscopic techniques. The study aimed to explore the anti‐hyperglycemic activity of the synthesized derivatives. The evaluation of anti‐hyperglycemic activity involved alloxan‐induced diabetic albino mice. Compound **14** and Compound **15** demonstrated potent activity, showing reductions of 39.19 % and 36.55 %, respectively, compared to pioglitazone (37.47 %) after 7 days of treatment. Molecular docking studies for Compound **14** and Compound **15** revealed their excellent binding affinity and pose towards the active sites of PTP1B (protein‐tyrosine phosphatase 1B), AR (aldose reductase), and PPAR‐γ (peroxisome proliferator‐activated receptor‐gamma). Additionally, the researchers predicted the ADME profile of the synthesized derivatives. Compound **14** and Compound **15** exhibited suitable pharmacokinetic and drug‐likeness properties, and these derivatives demonstrated good oral bioavailability. In conclusion, the researchers suggested that Compound **14** and Compound **15** hold promise for further evaluation of their anti‐hyperglycemic potential.^[79]^

**Figure 17:**
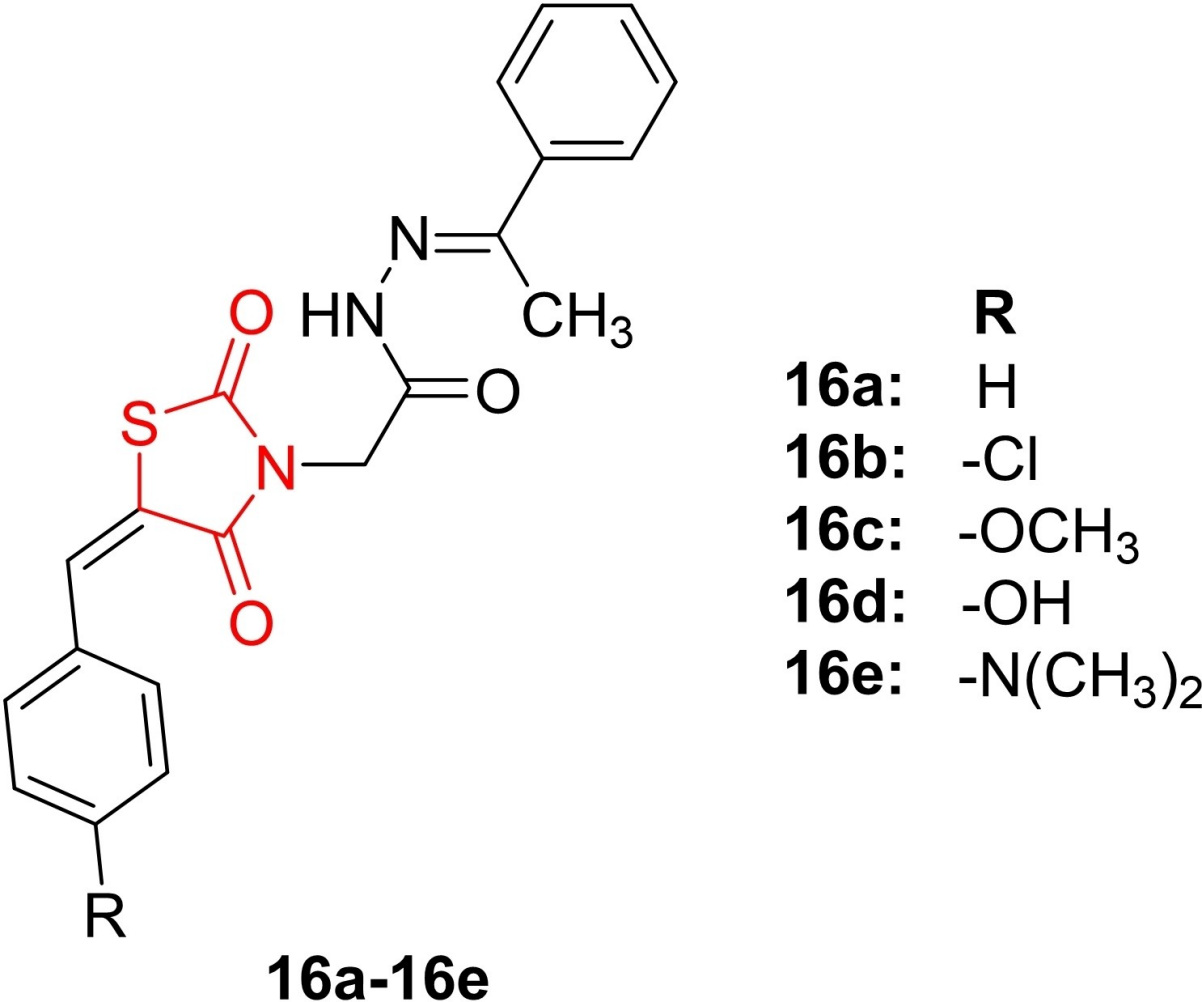
Benzylidene‐2,4‐thiazolidinedione based antidiabetic compounds.

Najmi *et al*., reported the design and synthesis of new benzylidene‐2,4‐thiazolidinedione derivatives (**16 a**–**16 e**, Figure 17), exploring their potential antidiabetic activity as partial PPAR‐γ agonists. The study included an acute oral toxicity test conducted on male Swiss albino mice, revealing that the synthesized derivatives showed no signs of toxicity after 14 days of administration. This indicated that the median lethal dose (LD_50_) of these derivatives exceeded 500 mg/kg. Additionally, an oral glucose tolerance test on normal mice demonstrated that compound **16 e** exhibited the most significant decrease (59.8 %) in blood glucose level at 100 mg/kg after 120 minutes, surpassing the effect of rosiglitazone (41.5 %). *In vivo* antidiabetic activity was assessed using an STZ‐induced diabetic model, revealing that compounds **16 d** and **16 e** were the most potent, resulting in a % decrease in FBG (fasting blood glucose level) of 59.3 % and 55.7 %, respectively, compared to rosiglitazone (% decrease=51.5 %). Importantly, the synthesized derivatives did not induce considerable weight gain in the treated animals.

Molecular docking studies showed that compounds **16 c**, **16 d**, and **16 e** exhibited good binding affinity towards the active site of PPAR‐γ, with binding energies of −10.1, −10.0, and −8.3 kcal/mol, respectively. These derivatives interacted with the receptor via hydrophobic interactions. In conclusion, Najmi et al. suggested that compounds **16 d** and **16 e** warrant further evaluation for their antidiabetic potential as partial PPAR‐γ agonists.^[80]^

**Figure 18:**
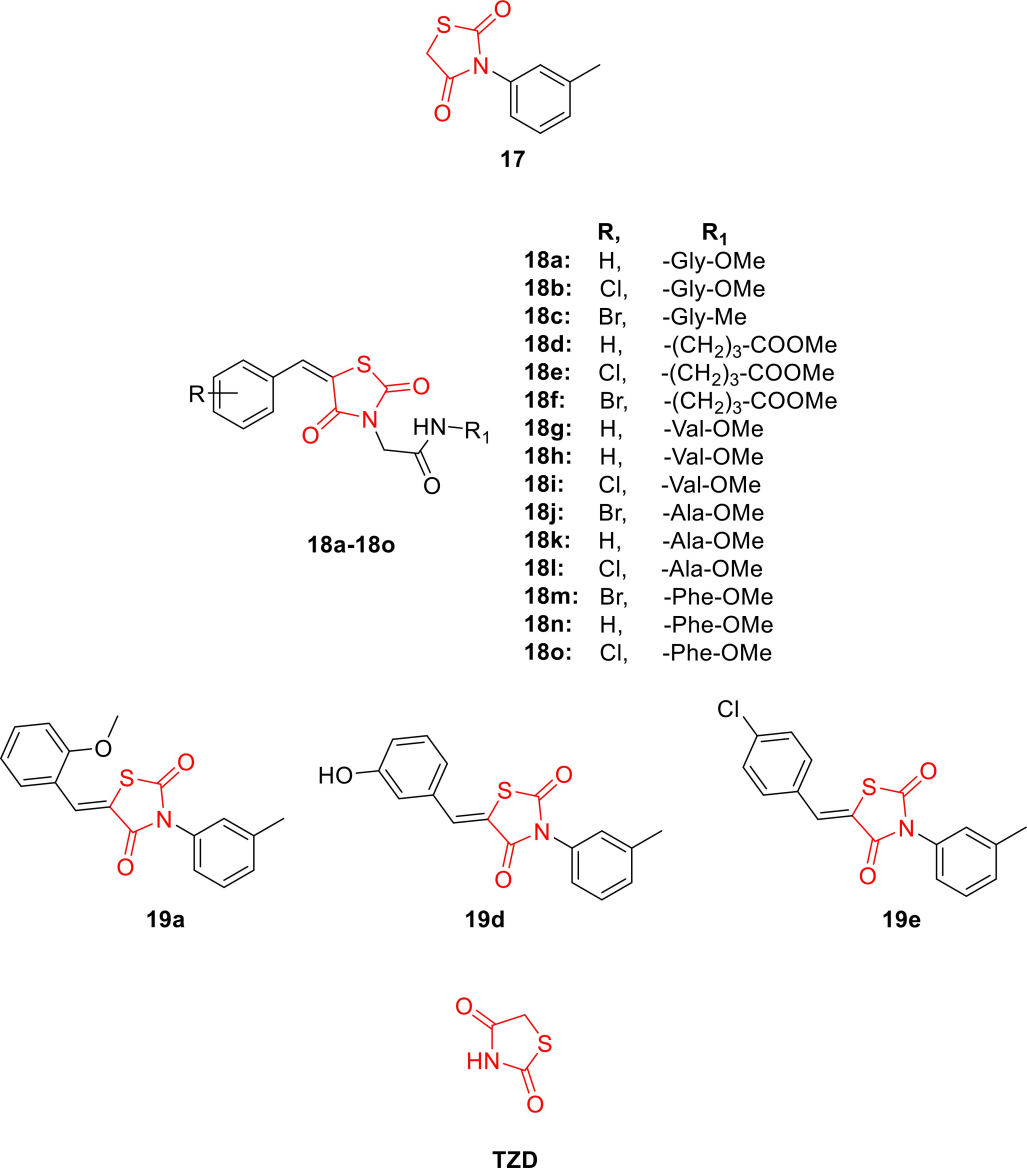
5‐arylidene‐3‐m‐tolyl thiazolidine‐2,4‐dione based antihyperglycemic agents.

Jahan *et al*., reported the design and synthesis of substituted 5‐arylidene‐3‐m‐tolyl thiazolidine‐2,4‐dione derivatives (**19 a**–**19 e**, Figure 18) in a two‐step process employing morpholine as a catalyst. In the initial step, phenyl thiourea (1) was treated with chloroacetic acid (2) in the presence of hydrochloric acid (3) to yield 3‐(m‐tolyl) thiazolidine‐2,4‐dione (4). Subsequently, compound **17** underwent a Knoevenagel condensation reaction with various aromatic aldehydes (**18 a**–**18 e**) in the presence of morpholine to produce compounds **19 a**–**19 e**. The chemical structure of the synthesized derivatives was characterized using IR, H‐NMR, and C‐NMR spectroscopy. The synthesized derivatives were subjected to investigation for their anti‐diabetic activity. Molecular docking studies revealed that compounds **19 a**, **19 d**, and **19 e** exhibited excellent binding affinity towards the active site of PPAR‐γ (peroxisome proliferator‐activated receptor‐gamma) with binding energies of −8.2, −8.5, and −8.9 kcal/mol, respectively, in comparison to epalrestat (−7.9 kcal/mol). In conclusion, the researchers suggested that compounds **19 a**, **19 d**, and **19 e** warrant further investigation for their anti‐diabetic potential and other potential biological activities.^[81]^

### 4.2 Anticancer Activity

**Figure 19:**
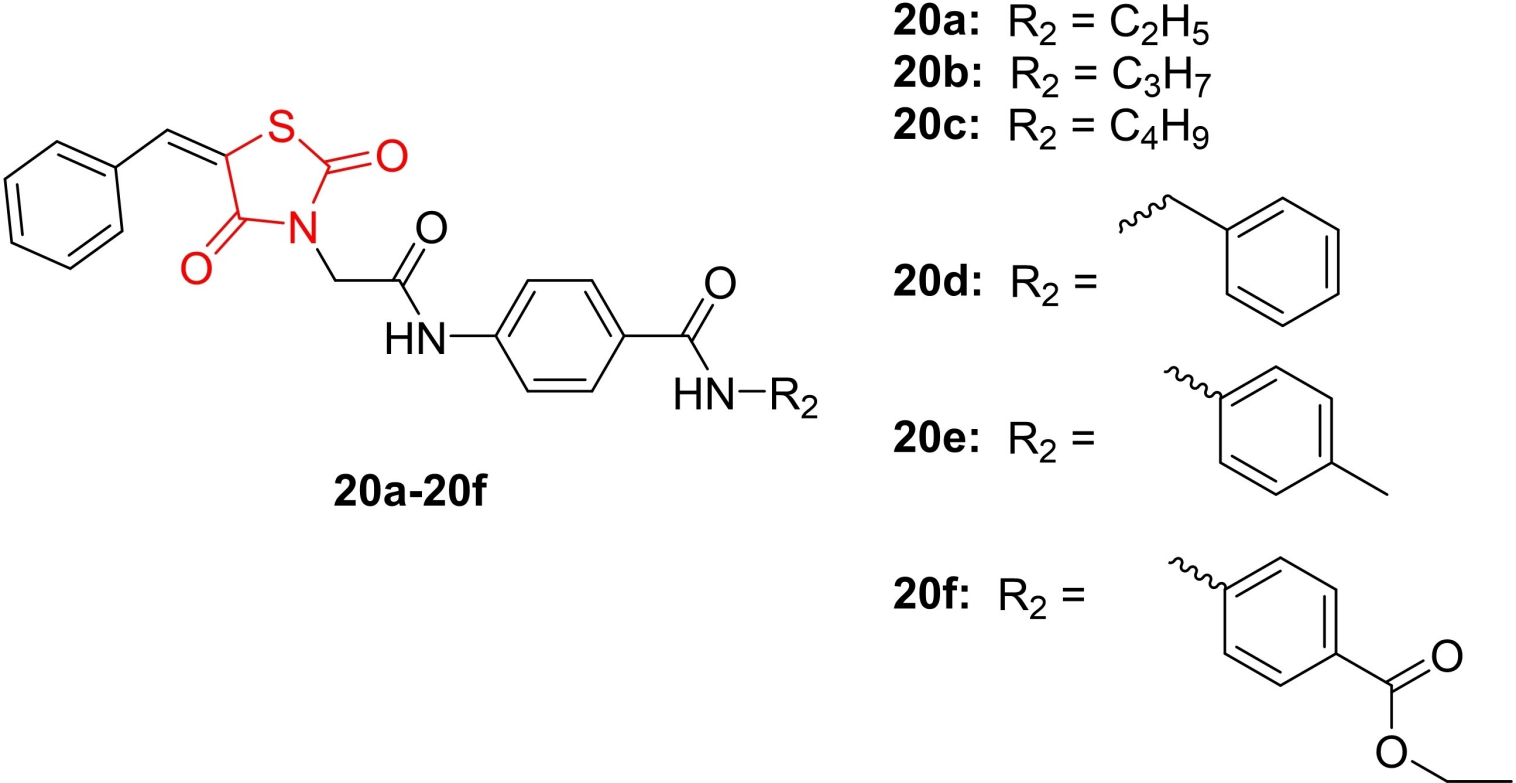
5‐benzylidenethiazolidine‐2,4‐dione based anticancer agents.

El‐Adl *et al*., reported on the design and synthesis of 5‐benzylidenethiazolidine‐2,4‐dione derivatives (**20 a**–**20 f**, Figure 19). The synthesized compounds underwent investigation for their anticancer activity against HepG2, HCT‐116, and MCF‐7 cancer cell lines using the MTT colorimetric assay. Among the tested cell lines, MCF‐7 was identified as the most sensitive to the influence of the newly synthesized derivatives. Compound **20 f** emerged as the most potent derivative against HepG2 (IC_50_=11.19±0.8 μM), HCT‐116 (IC_50_=8.99±0.7 μM), and MCF‐7 (IC_50_=7.10±0.4 μM) cancer cell lines, respectively. While compound **20 f** displayed lower activity than sorafenib against HepG2 (IC_50=_9.18±0.6 μM) and HCT‐116 (IC_50_=5.47±0.3 μM), it showed nearly the same activity against MCF‐7 (IC_50_=7.26±0.02 μM). Additionally, this compound exhibited lower activity than doxorubicin against HepG2 (IC_50=_7.94±0.6 μM) and HCT‐116 (IC_50_=8.07±0.8 μM), but nearly the same activity against MCF‐7 (IC_50_=6.75±0.4 μM). The active derivatives were further investigated for their inhibitory activity against VEGFR‐2. Compound **20 f** stood out as the most potent derivative, inhibiting VEGFR‐2 with an IC_50_ value of 0.22±0.02 μM, compared to sorafenib with an IC_50_ value of 0.10±0.02 μM. Molecular docking studies supported these findings, revealing that compound **20 f** (−97.60 kcal/mol) exhibited the highest binding affinity towards VEGFR‐2 compared to sorafenib (−95.66 kcal/mol). In conclusion, the researchers suggested that compound **20 f** could be a valuable candidate for future investigation as a selective VEGFR‐2 inhibitor with higher anticancer properties.^[82]^

**Figure 20:**
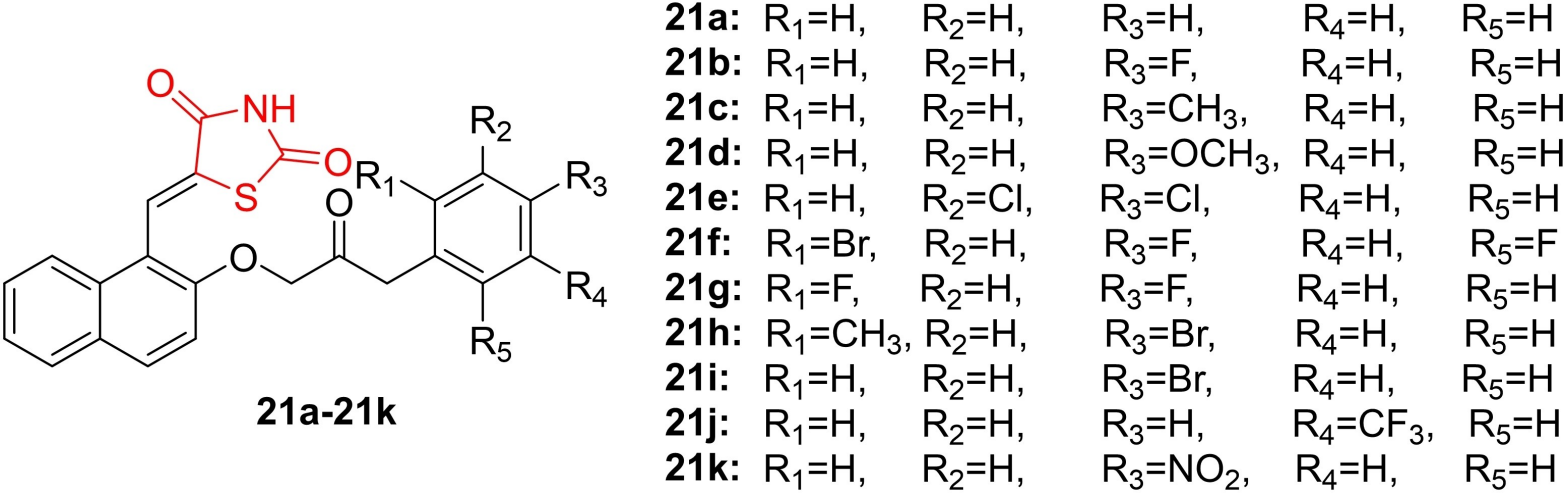
Naphthylidene clubbed 2,4‐thiazolidinedione potent anticancer agents.

Tilekar *et al*., reported on the design and synthesis of 5‐Naphthylidene‐2,4‐thiazolidinedione derivatives (**21 a**–**21 k**, Figure 20). The synthesized compounds underwent investigation for their selective HDAC8 inhibition activity using an HDAC enzyme inhibition assay. Notably, compound **21 k** and **21 h** were identified as the most potent selective inhibitors of HDAC8, with IC_50_ values of 2.7 μM and 6.3 μM, respectively. To assess the cytotoxic effects of the newly synthesized derivatives on leukemic cell lines (K562 and CEM), researchers conducted the MTT assay. Compound **21 a** demonstrated the most significant cytotoxic activity in leukemic cell lines, with IC_50_ values of 0.42 mM (K562) and 13.94 μM (CEM), respectively. Paclitaxel served as the reference drug with IC_50_ values of 0.29 and 15.5 μM, respectively. The apoptosis induction produced by compounds **21 a** and **21 h** was studied using flow cytometry, revealing that these compounds arrested the cell cycle in the G2/M phase. Additionally, researchers investigated the cytotoxic effect of compounds **21 a** and **21 h** on non‐transformed or normal cells, determining that these compounds are safer for normal cells. Molecular docking studies and the calculation of the ADME profile for the newly synthesized derivatives were also performed. In conclusion, researchers suggested that compound **21 k** holds promise as a future candidate for further investigation as a selective HDAC8 inhibitor.^[83]^

**Figure 21:**
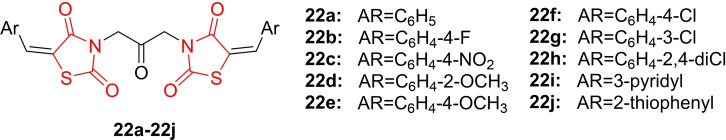
Arylidene‐appended thiazolidine‐2,4‐diones based anticancer compounds.

Crisan *et al*. reported on the design and synthesis of a new series of symmetric bis 5‐arylidene‐thiazolidine‐2,4‐diones (**22 a**–**22 j**, Figure 21). The synthesized derivatives underwent investigation for their inhibition activity against Ras and Raf oncoproteins, specifically assessing their antiproliferative activity. Molecular docking studies were conducted on K‐Ras, N‐Ras, and B‐Raf oncoproteins, revealing promising binding affinities for compound **22 b** (−10.3 kcal/mol) and **22 h** (−10.8 kcal/mol) to K‐Ras. The researchers highlighted that the newly synthesized derivatives exhibited the highest affinity for K‐Ras, slightly lower for B‐Raf, and the lowest for N‐Ras. The binding affinities for the most potent compounds, **22 b** and **22 h**, on different oncoproteins (K‐Ras, N‐Ras, and B‐Raf) were determined as −10.3, −6.9, and −9.8 kcal/mol for **22 b**, and −10.8, −6.8, and −10.2 kcal/mol for **22 h**, respectively. The researchers emphasized the complementarity between two in‐silico techniques to eliminate false positives in the results of molecular docking studies. They also stated that the research would be completed by testing these compounds on cancer cell lines with K‐Ras mutations to assess the correlation between in silico and *in vitro* results. In conclusion, the researchers suggested that compounds **22 b** and **22 h** should be further tested for their antiproliferative activity.^[84]^

**Figure 22:**
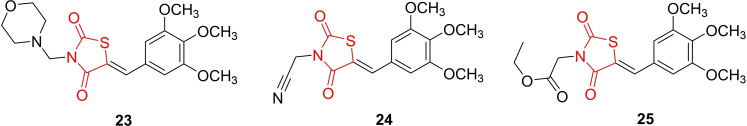
Disubstituted thiazolidine‐2,4‐diones based anticancer compounds.

El‐Kasef *et al*., reported the design and synthesis of (Z)‐3,5‐disubstituted thiazolidine‐2,4‐diones (**23**, **24**, **and 25**, Figure 22). The synthesized derivatives underwent investigation for their anti‐breast cancer activity against human breast cancer cell lines (MCF‐7 and MDA‐MB‐231) and human breast cancer cells using the MTT uptake method. The compounds were also evaluated against non‐cancerous breast cells to assess their potential for targeted therapy. Notably, Compound **23**, **24**, and **25** emerged as the most potent among all synthesized derivatives, inhibiting the proliferation of breast cancer cells in a dose‐dependent manner with IC_50_ values of 1.27, 1.50, and 1.31 μM, respectively. Researchers conducted flow cytometry and western blot analyses for Compound **23**, **24**, and **25** revealing their ability to induce apoptosis in human breast cancer cell lines (MCF‐7 and MDA‐MB‐231) without affecting normal non‐cancerous breast cells. The derivatives achieved this by decreasing the expression of anti‐apoptotic Bcl‐2 members (Bcl‐2, Bcl‐XL, and Mcl‐L) and increasing the expression of pro‐apoptotic Bcl‐2 members (Bak, Bax, and Bim). Furthermore, researchers found that Compound **23**, **24**, and **25** reduced the phosphorylation of AKT and mTOR, while also decreasing the expression levels of VEGF and HIF‐1α. In conclusion, the researchers suggested that Compound **23**, **24**, and **25** hold promise as potential candidates for future investigations as anti‐breast cancer agents.^[85]^

**Figure 23:**
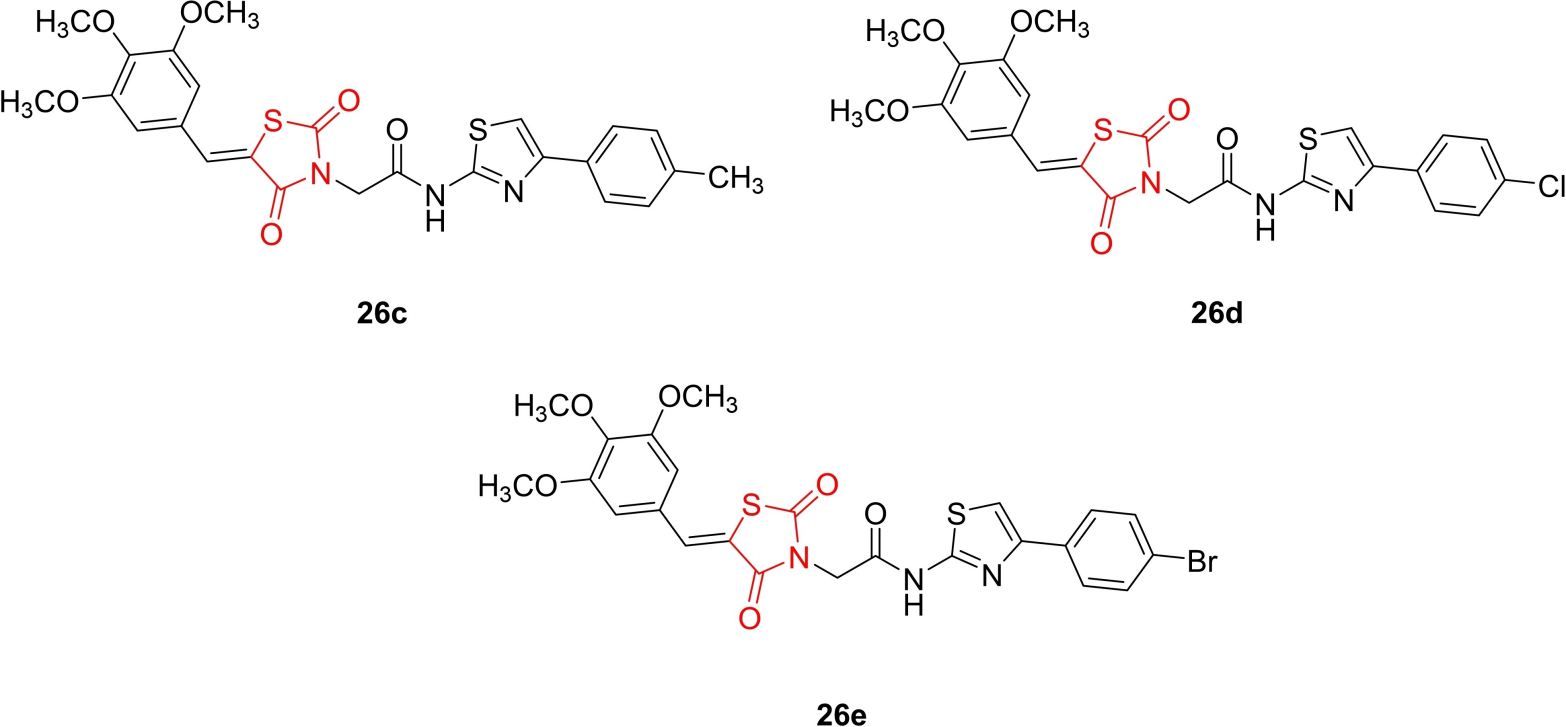
Trimethoxybenzene‐thiazole clubbed thiazolidine‐2,4‐dione as anticancer agents.

Sinicropi *et al*., reported the design and synthesis of novel thiazolidine‐2,4‐dione‐trimethoxybenzene‐thiazole (**26 c**, **26 d** and **26 e**, Figure 23) hybrids. The synthesized derivatives were investigated for their anticancer activity against breast cancer (MCF‐7 and MDA‐MB‐231) and melanoma (A2058) cancer cell lines. Researchers conducted the MTT assay and identified compound **26 e** as the most potent, with an IC_50_ value of 3.1 μM against MCF‐7 cancer cells, compared to ellipticine (IC_50_=1.15 μM), while showing no significant effect on normal MCF‐10 A cells. Molecular docking studies suggested human topoisomerases I and II (hTopos I and II) as potential targets, a finding further confirmed through enzymatic assays. Compound **26 e** exhibited excellent inhibition of both hTopo I and II, while compounds **26 c** and **26 d** selectively inhibited hTopo II. To investigate the mechanism of action, researchers performed the TUNEL assay, revealing that compound **26 e** induced apoptosis in MCF‐7 cancer cells by promoting the activation of caspases 3/7 and 9, mitochondrial destabilization, and cyt c migration into the cytoplasm. In conclusion, researchers recommended further evaluation of compound **26 e** for its anticancer potential by inhibiting topoisomerases I and II.^[86]^

**Figure 24:**
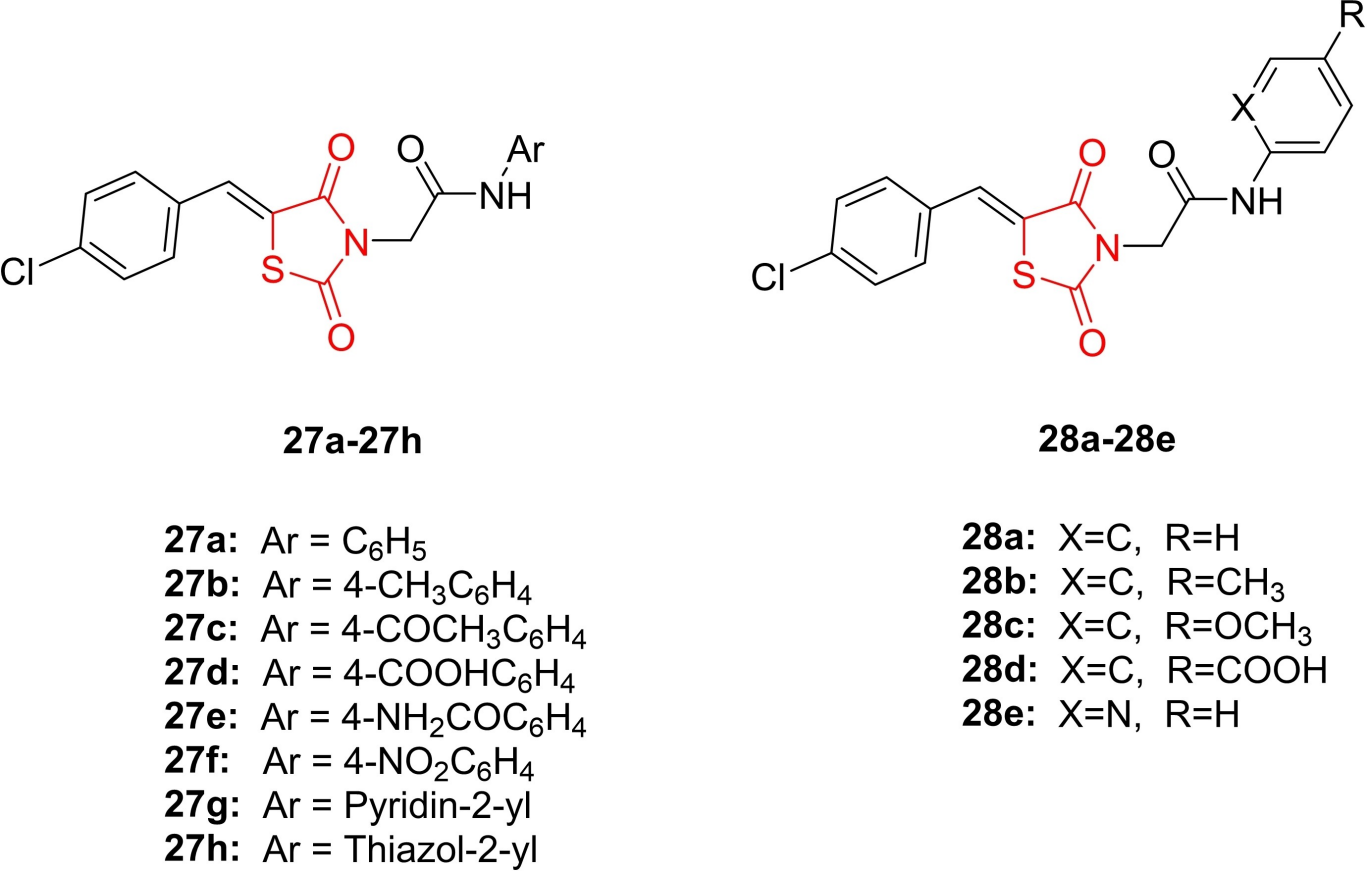
Novel thiazolidine‐2,4‐dione based anticancer compounds.

El‐Adl *et al*., reported the design and synthesis of novel thiazolidine‐2,4‐dione derivatives (**27 a**–**27 h** and **28 a**–**28 e**, Figure 24), investigating their anticancer activity against HepG2, HCT‐116, and MCF‐7 cancer cell lines. The HCT‐116 cancer cell line exhibited the highest sensitivity to the cytotoxic activity of the new derivatives. Compounds **28 d**, **27 e**, and **27 d** emerged as the most potent, with IC_50_ values ranging from 38.76 to 53.99 μM. Active antiproliferative derivatives (**27 a**–**27 h** and **28 a**–**28 e**) were further tested for their VEGFR‐2 inhibitory potential. These derivatives demonstrated substantial inhibitory activity, with IC_50_ values ranging from 0.26 to 0.72 μM. Specifically, compounds **28 d**, **27 e**, and **27 d** inhibited VEGFR‐2 with IC_50_ values ranging from 0.26‐0.29 μM, nearly three times that of sorafenib (0.10 μM). Researchers evaluated the ADMET profile, confirming that compounds **28 d**, **27 e**, and **27 d** adhered to Lipinski's rules and exhibited comparable intestinal absorptivity in humans. Importantly, these compounds did not inhibit cytochrome P3 A4. Compound **28 d**, **27 e**, and **27 d** were anticipated to have prolonged dosing intervals compared to sorafenib and doxorubicin. Ultimately, researchers found that compounds **27 d** and **28 d** displayed a wide therapeutic index and higher selectivity of cytotoxicity against cancer cells compared to normal cells. The results suggest that compounds **28 d**, **27 e**, and **27 d** hold promise for further investigation as potential anticancer agents.^[87]^

**Figure 25:**
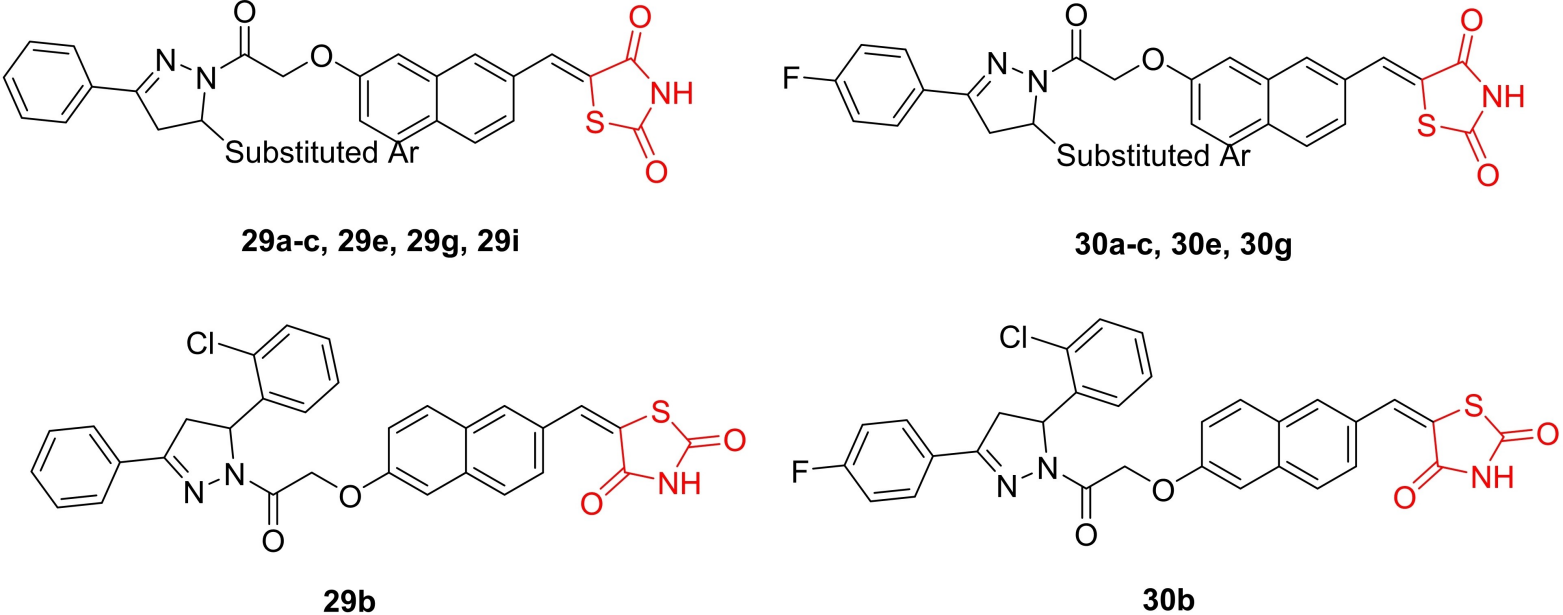
Pyrazoline containing thiazolidinediones based antiangiogenic agents.

Upadhyay *et al*., reported the design and synthesis of diaryl pyrazoline thiazolidinediones (**29 a**–**29 c**, **29 e**, **29 g**, **29 i** and **30 a**–**30 c**, **30 e**, **30 g**, Figure 25), featuring two series with naphthyl and pyridyl linkers. The synthesized derivatives were scrutinized for their anticancer activity and dual inhibition of VEGFR‐2 and HDAC4. In enzyme inhibition assays, compounds **29 b** and **30 b** emerged as the most potent against VEGFR‐2 (IC_50_=5 μM) and HDAC4 (IC_50_=0.34 and 0.36 μM). The researchers observed that compounds **29 b** and **30 b** stabilized HDAC4, confirmed by a shift in melting temperatures indicating interaction. In the MTT assay, compounds **29 b** and **30 b** exhibited significant anti‐angiogenic potential against MCF‐7, K562, A549, and HT‐29 with IC_50_ values ranging between 5.83–24.40 μM and 8.20‐16.92 μM, respectively. The anti‐angiogenic potential was further corroborated through various assays, with compound **30 b** showing superior inhibition of HUVEC proliferation and capillary tube formation compared to compound **29 b**. In *in vivo* assays on CAMs, compound **30 b** demonstrated potent attenuation of neovascularization compared to **29 b**. Western blot analysis revealed that compound **30 b** decreased phosphorylated VEGFR‐2 and HDAC4 expression levels and increased cleaved caspase‐3 expression. Additionally, compound **30 b** exhibited equivalent efficacy to doxorubicin in reducing tumor growth and volume in HT‐29 tumor xenograft models. The researchers concluded that compound **30 b** warrants further evaluation for its anticancer potential.^[88]^

**Figure 26:**
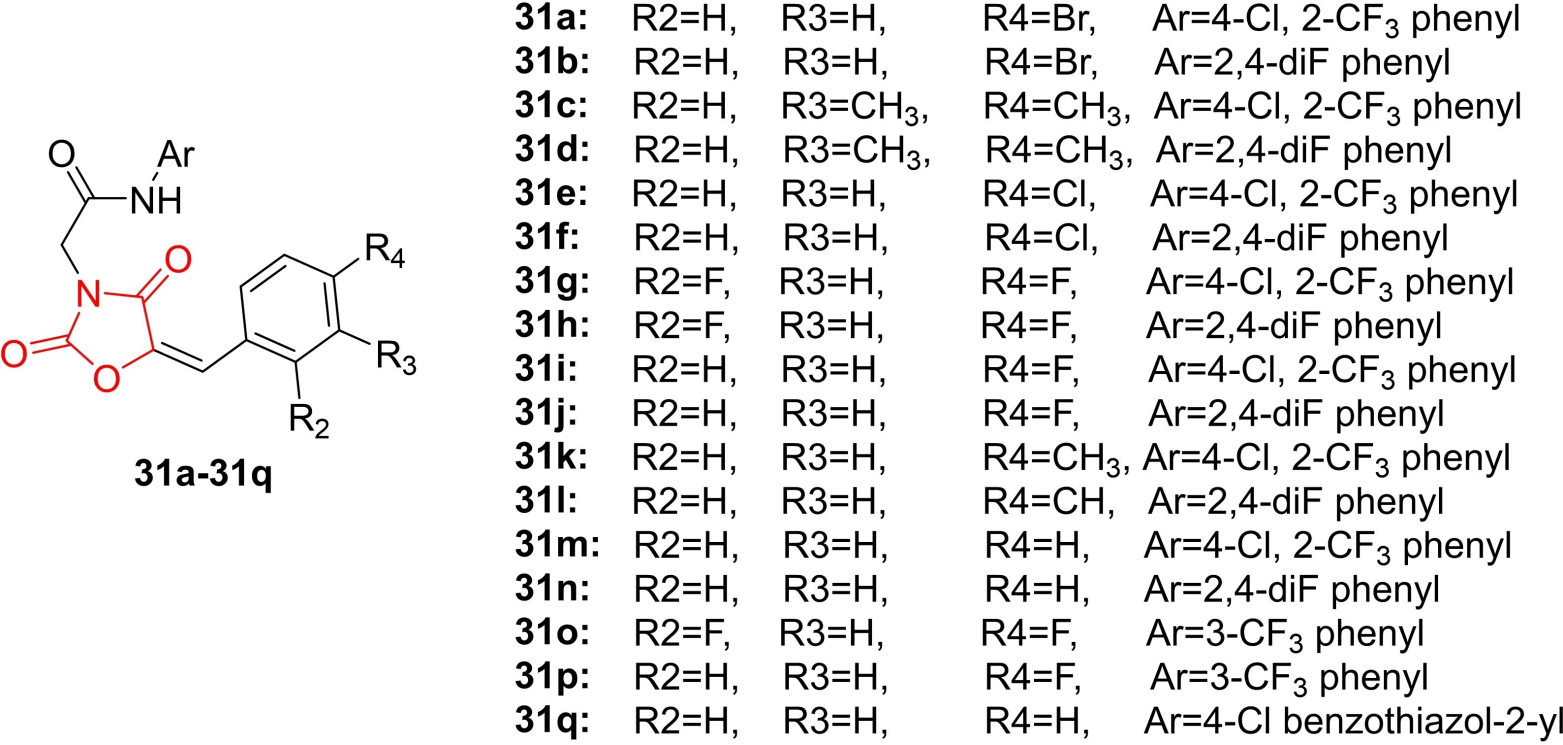
N‐substituted benzylidene containing thiazolidinedione based cytotoxic agents.

Tilekar *et al*., reported on the design and synthesis of N‐substituted benzylidene thiazolidinedione derivatives (**31 a**–**31 q**, Figure 26). The synthesized compounds underwent investigation for their GLUT inhibition property, followed by *in vitro* cytotoxicity determination in leukemic cell lines. Three compounds (**31 e**, **31 p**, and **31 q**) exhibited inhibition of GLUTs.

Compound **G5** emerged as the most active among the three derivatives, inhibiting all three GLUT types: GLUT1 (IC_50_=5.4±1.3 μM), GLUT4 (IC_50_=9.5±2.8 μM), and GLUT5 (IC_50_=34.5±2.4 μM), respectively. Molecular docking studies were performed by docking compounds **31 e**, **31 p**, and **31 q** to the inward‐ and outward‐facing structural models of GLUT1. The results indicated that these compounds block glucose access to the active site in both transporter conformations. Further investigations revealed that compound **31 e** inhibited the proliferation of leukemia CEM cells at a low micromolar range (IC_50_=13.4 μM) while exhibiting safety for normal blood cells. The progression of the CEM cell cycle after treatment with **31 e** was explored, indicating cell accumulation in the G2/M phase. Flow cytometric apoptosis studies demonstrated that compound **31 e** induced both early and late‐stage apoptosis in CEM cells. In conclusion, researchers suggested that compound **31 e** holds promise as a future candidate for further investigation as a GLUT inhibitor and anticancer agent.^[89]^

**Figure 27:**
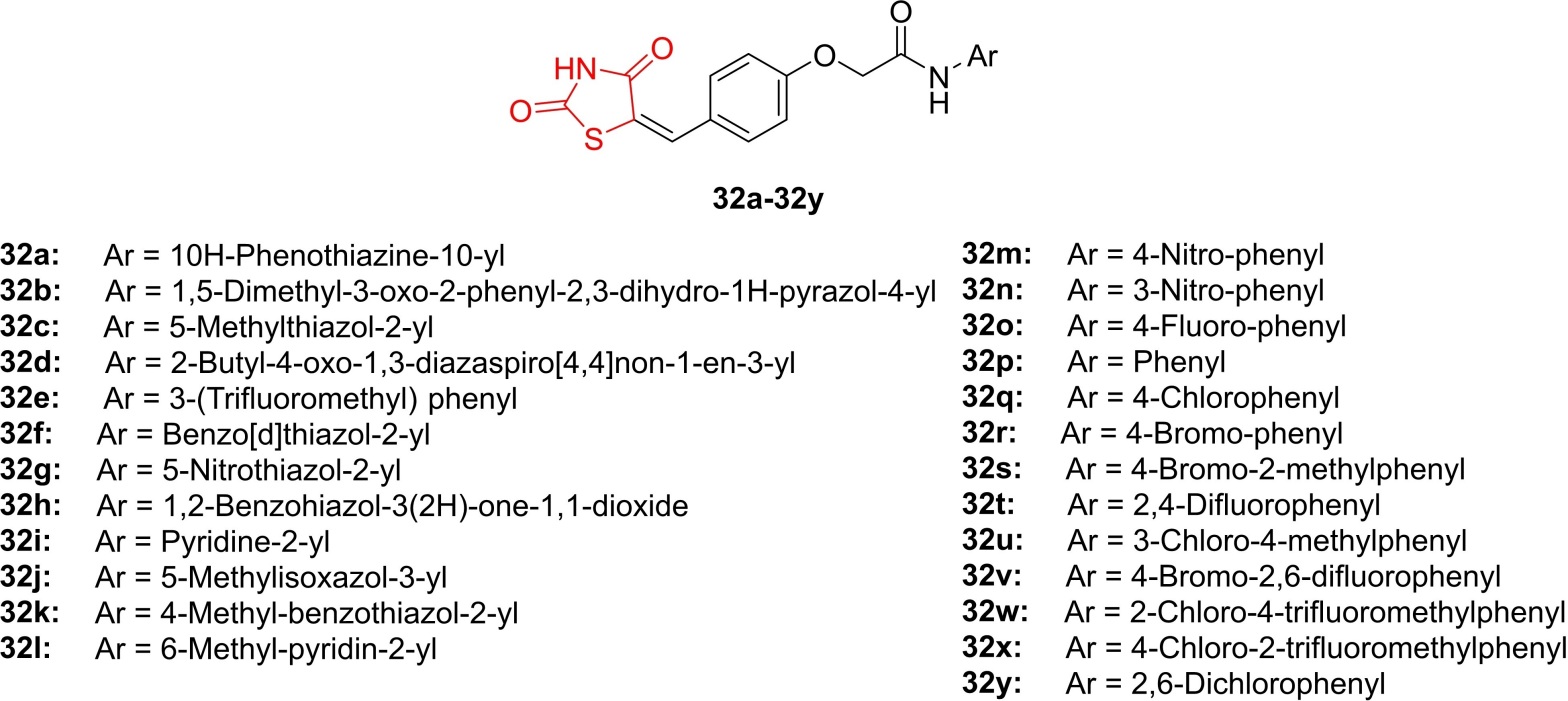
Benzylidene clubbed thiazolidinedione based anticancer compounds.

Joshi *et al*., reported the design and synthesis of benzylidene thiazolidinedione derivatives (**32 a**–**32 y**, Figure 27) and explored their anticancer activity across a panel of cancer cell lines (HOP62, K562, GURAV, KB2, Hep G2, MCF‐7, PC3, and their variants). In the *in vitro* cytotoxicity analysis, compounds **32 t** and **32 x** emerged as the most potent antiproliferative agents against myeloid leukemic cells (K562), displaying GI_50_ values of 0.9 and 0.23 μM, respectively, in comparison to doxorubicin (GI_50_<0.1 μM). The researchers noted that compounds **32 t** and **32 x** induced cell cycle arrest of K562 cancer cells in the G0/G1 phase in a time‐ and dose‐dependent manner.

Western blot analysis revealed that these compounds inhibited the expression of cell proliferation markers such as PCNA and cyclin D1. Additionally, compound **32 x** up‐regulated apoptosis markers, including cleaved PARP1 and activated caspase 3, suggesting a mechanism for its antiproliferative effects. *In vitro* combination studies of **32 t** and **32 x** with imatinib demonstrated increased antitumor activity. *In vivo* cytotoxicity analysis using a K562 xenograft model showed promising effects for compounds **32 t** and **32 x** alone, as well as in combination with imatinib. Combination treatment exhibited superior efficacy compared to imatinib or test compound treatment alone. In conclusion, the researchers suggested that compounds **32 t** and **32 x** warrant further investigation for their anticancer potential, particularly in enhancing the antitumor effect of imatinib.^[90]^

**Figure 28:**
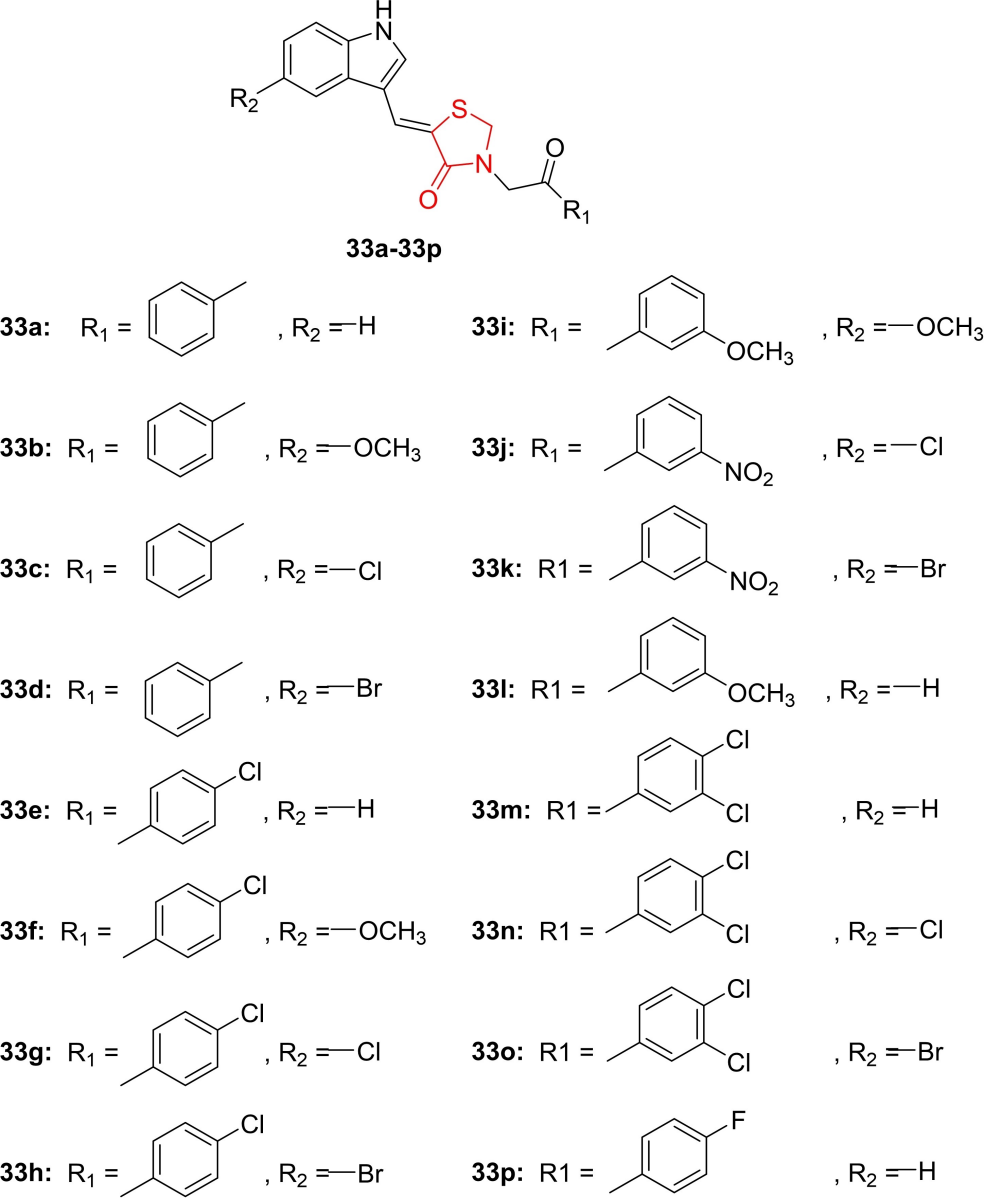
Indole‐appended thiazolidine‐2,4‐dione based antineoplastic compounds.

Alagoz *et al*., conducted a study on the design and synthesis of 5‐((5‐substituted‐1H‐indole‐3‐yl)methylene)‐3‐(2‐oxo‐2‐(3/4‐substituted‐phenylethyl)‐thiazolidine‐2,4‐dione derivatives (**33 a**–**33 p**, Figure 28). The synthesized compounds were investigated for their antineoplastic activity as CDK6 inhibitors. In cytotoxicity analysis on the MCF‐7 cancer cell line, compounds **33 g** and **33 j** emerged as the most potent, with IC_50_ values of 8.52 and 14.60 μM, respectively, in comparison to vincristine (IC_50_=1 μM). The researchers also explored the gene expression levels in MCF‐7 cells, focusing on 48 genes associated with various cellular functions. These derivatives significantly altered the expression levels of 21 genes, particularly impacting CDK6 expression. Molecular docking studies revealed that compounds **33 g** and **33 j** exhibited good binding affinity towards the targeted receptor, suppressing the CDK6 gene with binding scores of −8.8 and −8.6 kcal/mol. The compounds also demonstrated favorable drug‐likeness and pharmacokinetic properties. In conclusion, compounds **33 g** and **33 j** were identified as candidates for further evaluation due to their promising antineoplastic potential as CDK6 inhibitors.^[91]^

**Figure 29:**
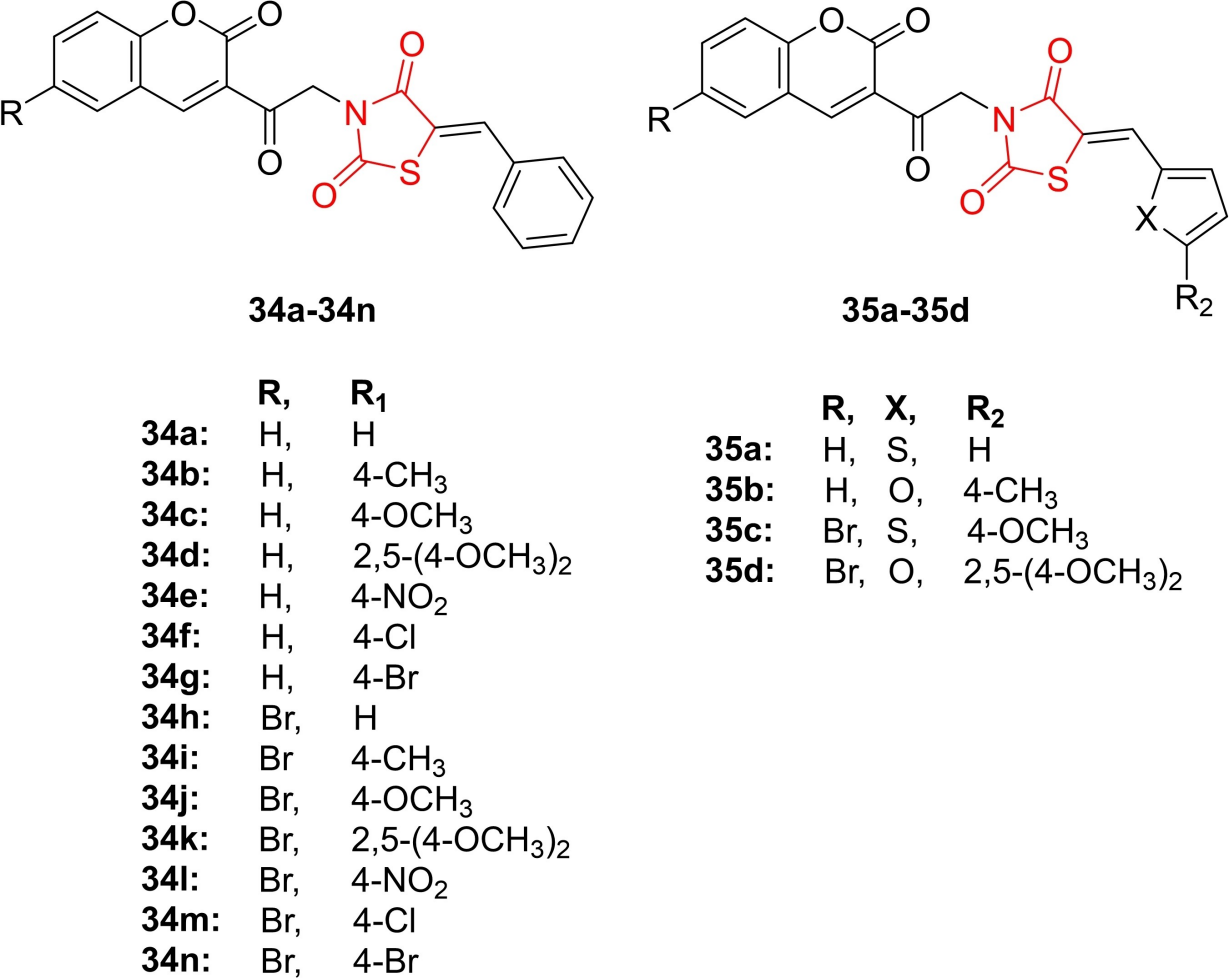
Coumarin containing 2,4‐thiazolidinedione based anticancer agents.

Eldehna *et al*., conducted a study on the design and synthesis of 2,4‐thiazolidinedione‐tethered coumarin derivatives (**34 a**–**34 n** and **35 a**–**35 d**, Figure 29). The chemical structures of the synthesized derivatives were characterized using H‐NMR, C‐NMR, and IR spectroscopic techniques. The primary focus of investigation was on their anticancer activity, particularly their inhibition of cancer‐associated carbonic anhydrase IX and XII. In the carbonic anhydrase inhibition assay, researchers identified compound **34 a**, **35 a**, **35 b**, and **35 c** as potent inhibitors of hCA IX (IC_50_=0.48–0.82 μM) and XII (IC_50_=0.4–1.1 μM) in comparison to acetazolamide (IC_50_=25 and 5.7 μM). Importantly, none of the synthesized derivatives exhibited inhibitory effects on off‐target hCA I and II isoforms. Further evaluation included *in vitro* antiproliferative activity against the MCF‐7 cancer cell line. Compound 11a emerged as the most potent, with an IC_50_ value of 0.48 μM compared to staurosporine (IC_50_=2.44 μM). Researchers noted that compound **35 a** induced apoptosis and arrested the cell cycle at S and G0‐G1 phases in MCF‐7 breast cancer cells. In conclusion, compound **35 a** was identified as a promising candidate for further exploration of its anticancer potential through the inhibition of hCA IX and XII isoforms.^[92]^

**Figure 30:**
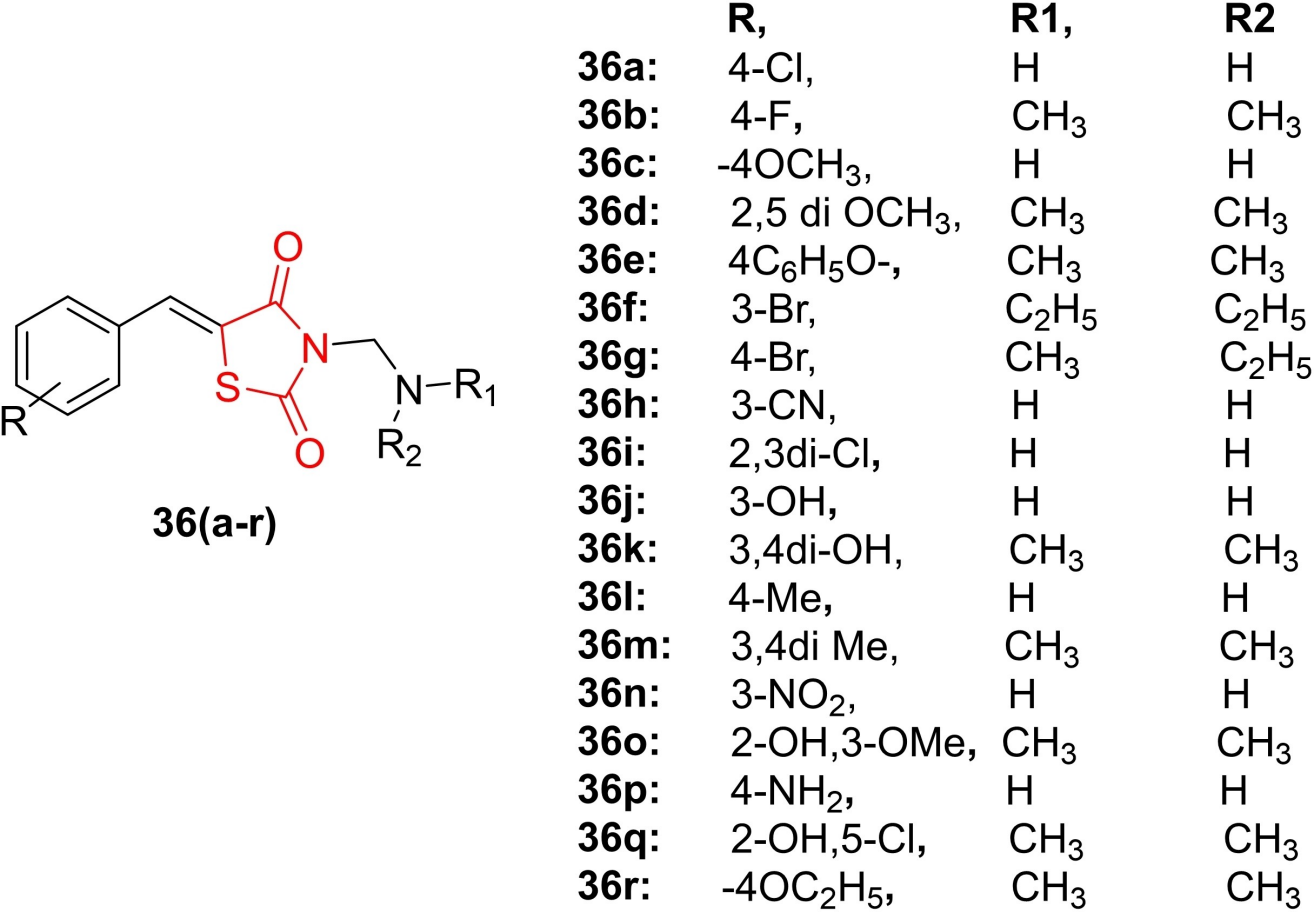
Benzylidene clubbed thiazolidine‐2,4‐dione based cytotoxic compounds.

Kumar *et al*., reported the design and synthesis of novel 3‐(aminomethyl)‐5‐benzylidenethiazolidine‐2,4‐dione derivatives (**36 a**–**36 r**, Figure 30) through Knoevenagel condensation with cyclization reaction. The chemical structure of the synthesized derivatives was characterized using H‐NMR, IR, and Mass spectroscopy. The study aimed to investigate their anticancer activity using the sulforhodamine B (SRB) method. *In vitro* antiproliferative activity was assessed against HeLa (cervical cancer cells) and HCT‐8 (colon carcinoma) cancer cell lines. Compound **36 i** demonstrated the highest potency against both cancer cell lines, with IC_50_ values of 0.007 and 0.011 μM, respectively, compared to Adriamycin (IC_50_=0.0001 and 0.0023 μM).Structure‐activity relationship (SAR) studies were conducted, comparing the activity of potent compounds (**36 a**, **36 b**, **36 f**, **36 i**, **36 n**, and **36 q**) with other derivatives. The analysis indicated that the presence of electronegative groups at the C‐2 and C‐3 positions of the phenyl ring contributed to the highest cytotoxic activity, particularly in compound 3i, against cervical cancer (HeLa) and colon carcinoma (HCT‐8) cell lines.

Conversely, substitution on the phenyl ring with electron‐donating groups decreased the anticancer activity. ADME (absorption, distribution, metabolism, and excretion) profiling of the synthesized derivatives, particularly compound **36 i**, revealed good physicochemical and drug‐likeness properties, along with favorable oral absorption characteristics. In conclusion, the researchers suggested that compound **36 i** merits further evaluation for its anticancer potential.^[93]^

### 4.3 Antimicrobial Activity

**Figure 31:**
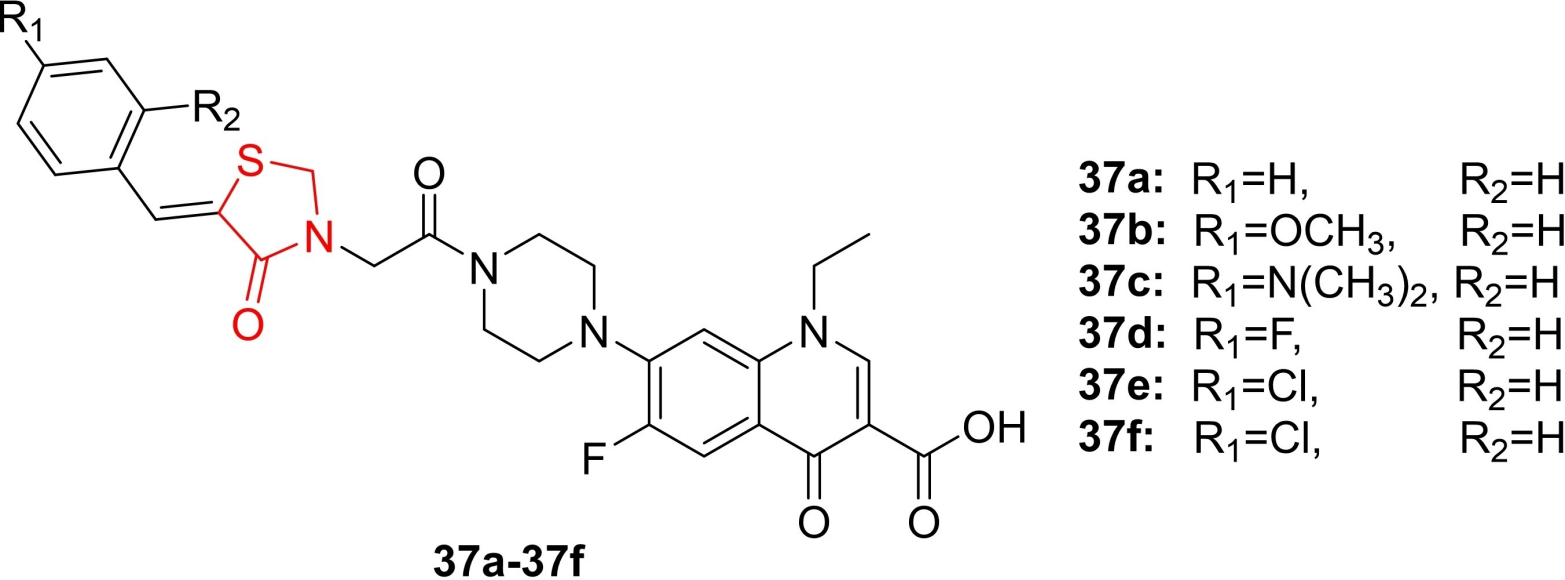
Piperazin containing thiazolidine‐2,4‐dione based antimicrobial compounds.

Marc and his colleagues conducted a study in which they designed and synthesized analogues of Piperazin‐4‐yl‐(acetyl‐thiazolidine‐2,4‐dione) Norfloxacin (**37 a**–**37 f**, Figure 31). These analogues were then docked into the active site of the DNA gyrase enzyme, which was isolated from E. coli. The researchers examined the antimicrobial activity and biofilm properties of the synthesized compounds. To assess antimicrobial activity, the researchers tested these compounds against a total of seven bacterial strains and two yeast strains (*C. albicans* and *C. parapsilosis*). Among these, four were gram‐positive bacteria (*S. aureus*, *L. monocytogenes*, *B. cereus* and *E. faecalis*) and three were gram‐negative bacteria (*E. coli* and *S. enteritidis*). The *in vitro* quantitative assay results indicated that compound **37 a** displayed the highest potency (IC_50_=0.125–2 μ/ml) compared to norfloxacin (IC_50_=0.0312–1 μ/ml). Additionally, it was observed that the p‐methoxy‐substituted compound (**37 b**) was well‐tolerated and maintained a similar antibacterial effect as the non‐substituted molecules. Regarding anti‐biofilm activity, the study revealed that the new derivatives exhibited varying degrees of anti‐biofilm activity, with a more pronounced effect against *S. aureus*. Molecular docking analysis demonstrated that all the compounds exhibited binding affinity within the range of −6.16 to −8.16 kcal/mol. A total of 200 conformations were explored for each compound, and the results indicated that the norfloxacin derivatives displayed a strong binding affinity for the DNA gyrase enzyme. Furthermore, an ADMET study showed that the synthesized compounds had favorable ADMET profiles. They were predicted to be orally bioavailable and had a low potential for toxicity.^[94]^

**Figure 32:**
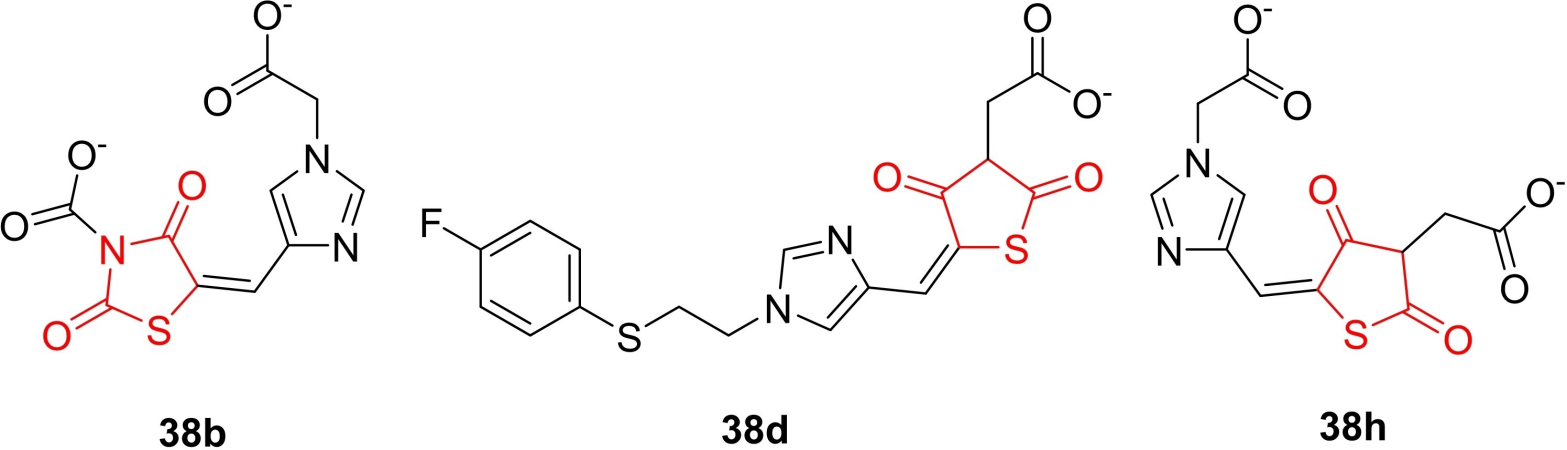
Imidazole clubbed 2,4‐thiazolidinedione based antimicrobial agents.

Moorthy et al. reported the design and synthesis of two series of 2,4‐thiazolidinedione derivatives (**38 a**–**38 d** and **38 e**–**38 h**,, Figure 32) containing substituted imidazoles, as well as one series of 5‐substituted 2,4‐thiazolidinedione derivatives. The synthesized compounds underwent investigation for their antibacterial activity against *Staphylococcus aureus* ATCC‐9144, *Staphylococcus epidermidis* ATCC‐155, *Escherichia coli* ATCC‐25922, and *Pseudomonas aeruginosa* ATCC‐2853, along with antifungal activity against *Aspergillus niger* ATCC‐9029 and Aspergillus fumigatus ATCC‐46645 using the paper disc diffusion technique. All synthesized compounds demonstrated activity against the tested microorganisms, with MIC values ranging as follows: *S. aureus* (1.9–23.7 μg/ml), *S. epidermidis* (1.4–22.2 μg/ml), E. coli (1.6–22.6 μg/ml), *P. aeruginosa* (0.56–22.4 μg/ml), *A. niger* (7.9–22.9 μg/ml), and *A. fumigatus* (2.3–24.6 μg/ml). Compound **T2** emerged as the most potent, displaying MIC values against S. aureus (1.9 μg/ml), *S. epidermidis* (1.4 μg/ml), E. coli (1.6 μg/ml), *P. aeruginosa* (0.56 μg/ml), *A. niger* (8.8 μg/ml), and *A. fumigatus* (2.3 μg/ml). Compounds **38 d** and **38 h** also exhibited significant antimicrobial activity, comparable to standard drugs Ciprofloxacin and Ketoconazole.

The enhanced antimicrobial properties of **38 b**, **38 d**, and **38 h** were attributed to acetyl substitution at the 3^rd^ position and the presence of an imidazole moiety at the 5^th^ position of 2,4‐thiazolidinedione. Additionally, **38 b's** biacetate group increased its lipophilicity, contributing to its efficacy. Compounds **38 b**, **38 d**, and **38 h** emerged as promising candidates for further development in the design of thiazolidinedione‐based antimicrobial agents.^[95]^

**Figure 33:**
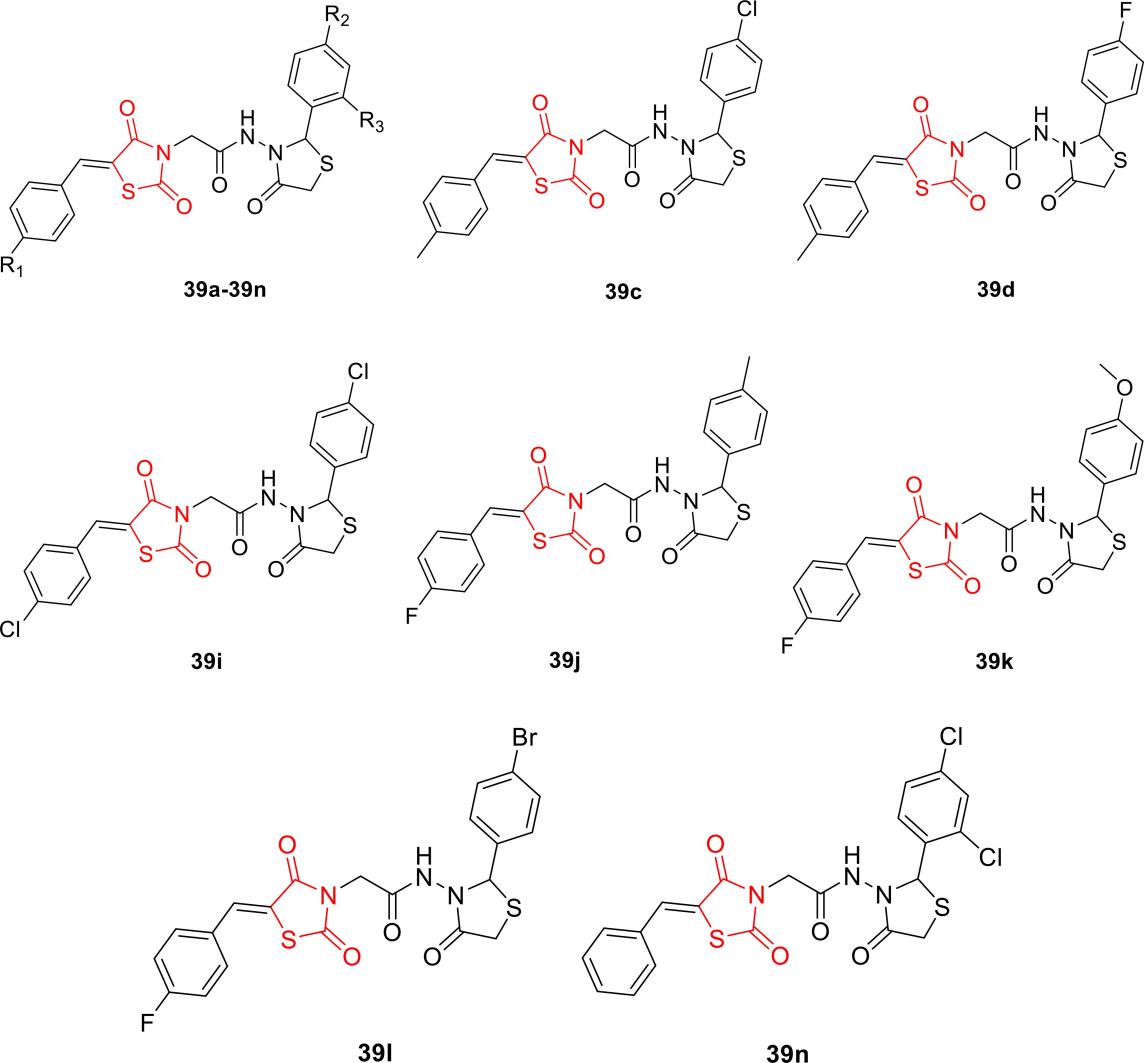
Arylidene‐incorporated 4‐thiazolidinedione based antimicrobial agents.

Kulkarni et al. reported the design and synthesis of arylidene‐incorporated 4‐thiazolidinedione derivatives (**39 a**–**39 n**, Figure 33). The synthesized compounds underwent investigation for their antimicrobial activity against three Gram‐positive bacteria (*Staphylococcus aureus*, *Bacillus cereus*, and *Micrococcus luteus*) and three Gram‐negative bacteria (*Pseudomonas fluorescens*, *Escherichia coli*, and *Flavobacterium devorans*). Among all the synthesized compounds, namely **39 c**, **39 d**, **39 i**, **39 j**, **39 k**, **39 l**, and **39 n**, were found to be the most potent, exhibiting MIC values in the range of 2–4 μM against all tested Gram‐positive and Gram‐negative strains. In comparison, standard antibacterial drugs such as Ampicillin (2‐16 μM), Kanamycin (2 μM), and Chloramphenicol (2 μM) were used for reference.

The researchers conducted cytotoxicity assessments and determined that all synthesized compounds showed non‐toxicity against HeLa and MCF‐7 cell lines. A structure‐activity relationship (SAR) study on all synthesized compounds (**39 a**–**39 n**) revealed that compounds containing methoxy, fluoro, chloro, and bromo groups at the para position of the phenyl ring displayed significant antimicrobial activity against both Gram‐positive and Gram‐negative bacteria. This observation suggests that the presence of these specific groups on the heterocyclic system enhances the pharmacological effectiveness. Consequently, compounds **39 c**, **39 d**, **39 i**, **39 j**, **39 k**, **39 l**, and **39 n** emerged as promising candidates for further development as antimicrobial compounds.^[96]^

**Figure 34:**
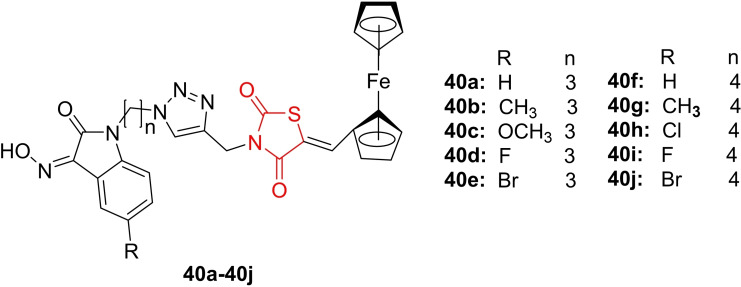
Isatin‐incorporated 4‐thiazolidinedione based antimicrobial compounds.

Yagnam *et al*., reported on the design and synthesis of bioactive isatin (oxime)‐triazole‐thiazolidinedione ferrocene molecular conjugates (**40 a**–**40 j**, Figure 34). The synthesized compounds underwent investigation for their antimicrobial activity against both gram‐positive bacteria (*Bacillus subtilis*, *Bacillus megaterium*, *Mycobacterium smegmatis*, *Klebsiella pneumonia*) and gram‐negative bacteria (*Escherichia coli*, *Salmonella typhi*, *Pseudomonas aeruginosa*, *Pseudomonas putida*). Streptomycin served as the reference for bacterial strains. Additionally, antifungal activity was assessed against Candida albicans and Aspergillus oryzae, with fluconazole used as the reference, employing the agar well diffusion method. Compounds **40 b**, **40 c**, **40 h**, and **40 i** emerged as the most potent against bacterial strains, exhibiting MIC values of 4 m/ml compared to streptomycin with a MIC value of 2 μ/ml. These compounds also demonstrated the highest antifungal activity, with MIC values of 4 and 32 mg/ml, in contrast to fluconazole with a MIC value of 16μg/ml. UV‐vis spectroscopy was employed by researchers to confirm the conjugation of ferrocene with the isatin moiety.

Additionally, electrochemical characterization studies revealed that the oxidation potentials for all isatin ferrocene hybrids **40**(**a**–**j**) were higher than those of the unsubstituted ferrocene. In conclusion, the researchers suggested that compounds **40 b**, **40 c**, **40 h**, and **40 i** hold promise as potent candidates for further development as antimicrobial agents.^[97]^

**Figure 35:**
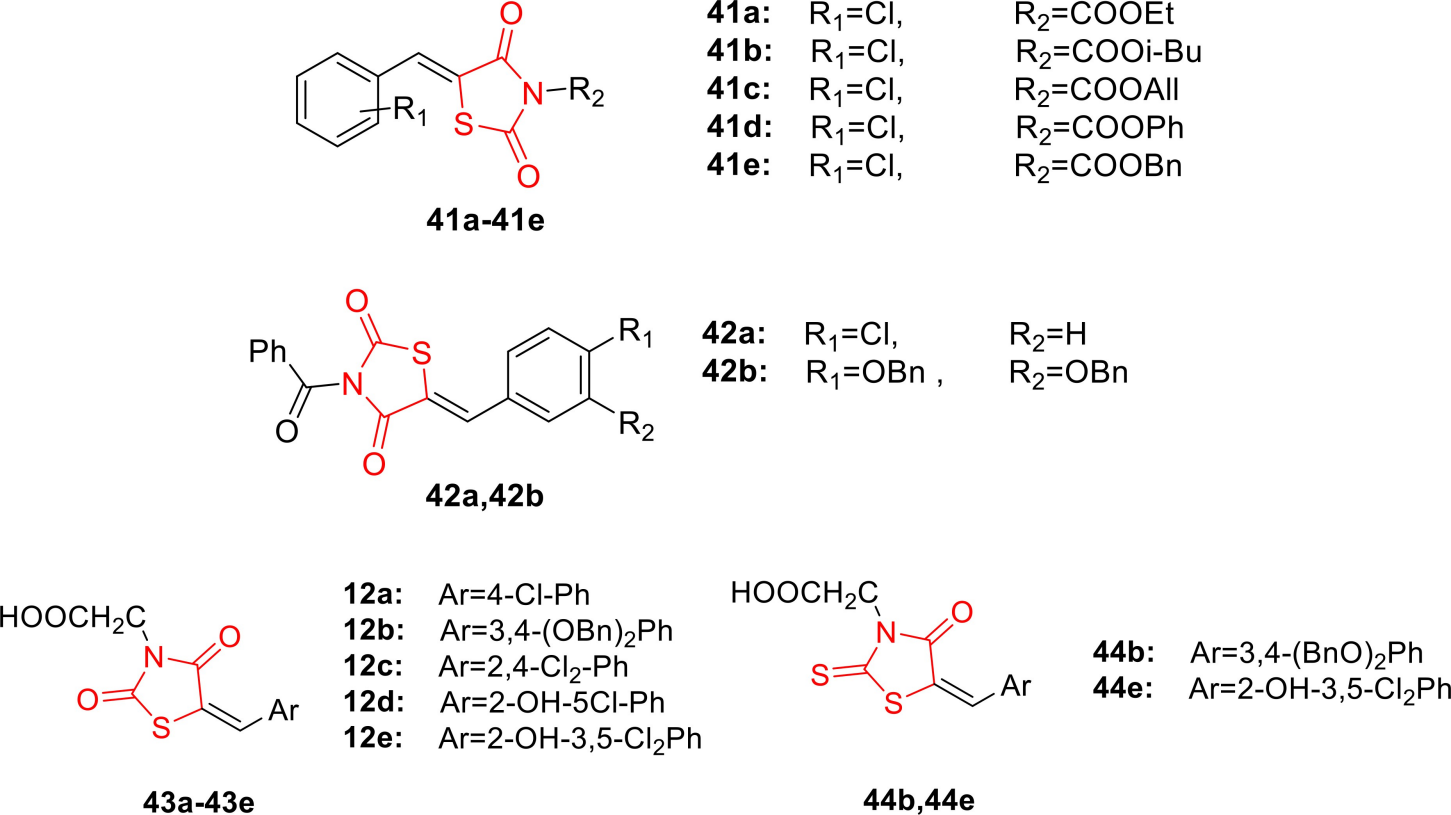
Mycosidine substituted thiazolidine‐2,4‐diones based antimicrobial compounds.

Levshin *et al*., reported on the design and synthesis of Mycosidine‐3,5‐substituted thiazolidine‐2,4‐diones (**41 a**–**41 e**, **42 a**–**42 b**, **43 a**–**43 e** and **44 b**, **44 e**, Figure 35) using Knoevenagel synthesis and the alkylation or acylation of the imide nitrogen. The synthesized compounds underwent testing for their antifungal activity in three rounds against filamentous fungi (*Trichophyton rubrum* and *Microsporum canis*), *Candida albicans*, *Aspergillus niger*, and *Aspergillus fumigatus*. Following the initial MIC determination test, researchers identified compounds **41 a**, **43 e**, and **44 e** as the most potent among all synthesized derivatives, with MIC ranges of 1–16 μg/L, 0.125‐16 μg/L, and 2–16 μg/L, respectively. In comparison, mycosidine exhibited a MIC range of 4–32 μg/L, and fluconazole had a range of 4–64 μg/L. Subsequently, researchers conducted a re‐evaluation assay for compounds **41 a**, **42 a**, and **43 e** using Candida species yeast. The results revealed that compound 12e (0.125‐64 μ/ml) was the most effective against Candida species compared to fluconazole (4‐32 μ/ml). Further investigations involved assessing the effect of compound 17b on living Candida cells using Scanning Ion Conductance Microscopy (SICM). The findings indicated that compound **44 b** caused the disruption of the Candida cell wall compared to mycosidine. In conclusion, researchers noted that the synthesized compounds exhibited both fungistatic and fungicidal effects, inducing morphological changes in Candida yeast cell walls. Additionally, they highlighted that mycosidine‘s antifungal action is dependent on glucose transport, suggesting a unique mechanism of action worthy of further investigation.^[98]^

**Figure 36:**
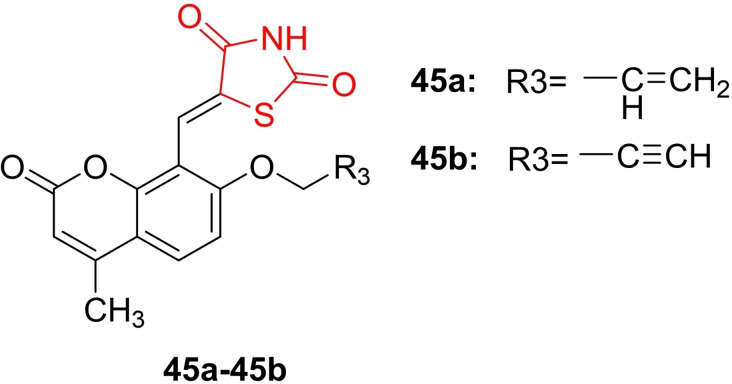
Coumarin clubbed thiazolidinedione based antimicrobial compounds.

Fang Hu *et al*., reported the design and synthesis of ethylenic conjugated coumarin thiazolidinedione derivatives (**45 a**–**45 b**, Figure 36), confirming their structures through H‐NMR, C‐NMR, and HRMS spectra. The synthesized derivatives were investigated for their antimicrobial activity. In the antimicrobial assay, compound **45 b** emerged as the most potent against methicillin‐resistant Staphylococcus aureus (MIC=0.006 mmol/ml) and drug‐resistant A. fumigates (MIC=0.012 mmol/ml), surpassing norfloxacin (MIC=0.025 mmol/ml) and fluconazole (MIC=1.672 mmol/ml). Researchers highlighted that compound **45 b** exhibited rapid bactericidal activity, prevented apparent drug resistance development in MRSA strains, and demonstrated low toxicity towards hepatocyte cells (LO2). Additionally, compound **45 b** was noted to inhibit MRSA growth by forming stable supramolecular complexes with bacterial DNA, hindering DNA replication. Molecular docking studies supported this mechanism, revealing that compound **45 b** interacted with bacterial DNA‐gyrase through hydrogen bonding. In conclusion, researchers recommended further investigation of compound **45 b** for its antibacterial activity.^[99]^

**Figure 37:**
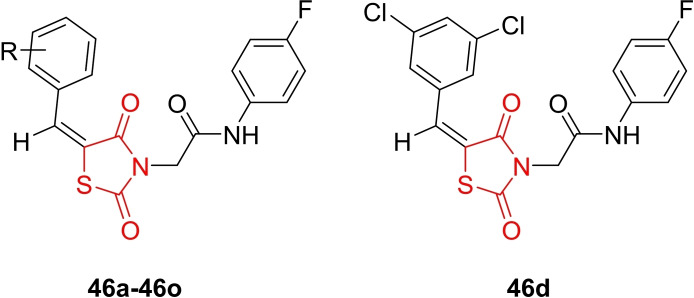
Arylidene‐appended thiazolidine‐2,4‐dione based antimicrobial compounds.

Joshi *et al*., reported the design and facile synthesis of a series of novel 5‐arylidene‐thiazolidine‐2,4‐dione derivatives (**46 a**–**46 o**, Figure 37), confirming their structures through H‐NMR, FT‐NMR, and IR spectroscopy. The derivatives were synthesized via a Knoevenagel condensation reaction using aromatic aldehydes, N‐substituted thiazolidine‐2,4‐diones, and alum as a catalyst. The antimicrobial activity of the synthesized derivatives against various strains, including gram‐positive bacteria (*Staphylococcus aureus* and *Streptococcus pyogenes*), gram‐negative bacteria (Escherichia coli and Pseudomonas aeruginosa), and the fungal strain Candida albicans, was investigated. In the antimicrobial assay, compound **46 d** exhibited the highest potency among all derivatives, displaying MIC values ranging between 4–16 mg/ml against *S. aureus*, *S. pyogenes*, *E. coli*, *P. aeruginosa*, and *C. albicans*, in comparison to ciprofloxacin (MIC=4 mg/ml) and ketoconazole (MIC=4 mg/ml). The researchers suggested that compound **46 d** holds potential for further modification and evaluation of its antimicrobial properties.^[100]^

**Figure 38:**
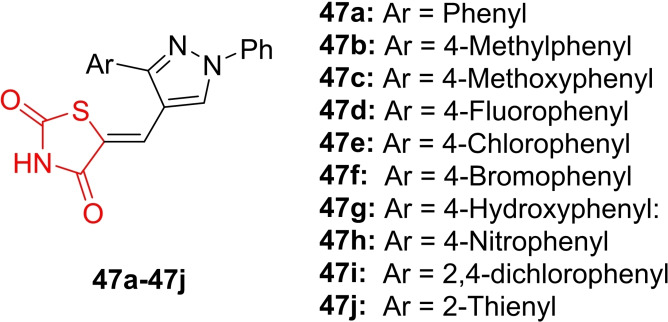
Pyrazolyl incorporated thiazolidinedione based antimicrobial agents.

Tummalacharla *et al*., reported the design and synthesis of pyrazolyl‐thiazolidinedione hybrids (**47 a**–**47 j**, Figure 38), characterizing their chemical structure using IR, H‐NMR, C‐NMR, MS, and elemental analysis. The synthesized derivatives were examined for antimicrobial activity against four bacterial strains (*Staphylococcus aureus*, *Bacillus subtilis*, *Pseudomonas aeruginosa*, and *Escherichia coli*) and two fungal strains (*Sclerotium rolfsii* and *Aspergillus niger*). In the antimicrobial assay, compounds **47 h** and **47 i** demonstrated significant antibacterial potential against all tested bacterial strains, yielding inhibition zones ranging between 11.0–15.0 mm, compared to norfloxacin.

Additionally, compound **47 i** exhibited notable antifungal activity, with inhibition zones ranging between 8.8‐11.3 mm, as compared to ketoconazole. Molecular docking studies revealed that compounds **47 h** and **47 i** displayed moderate docking scores and played a pivotal role in forming hydrogen bond interactions. In conclusion, the researchers suggested that compounds **47 h** and **47 i** could be further refined and assessed for their antimicrobial potential.^[101]^

**Figure 39:**
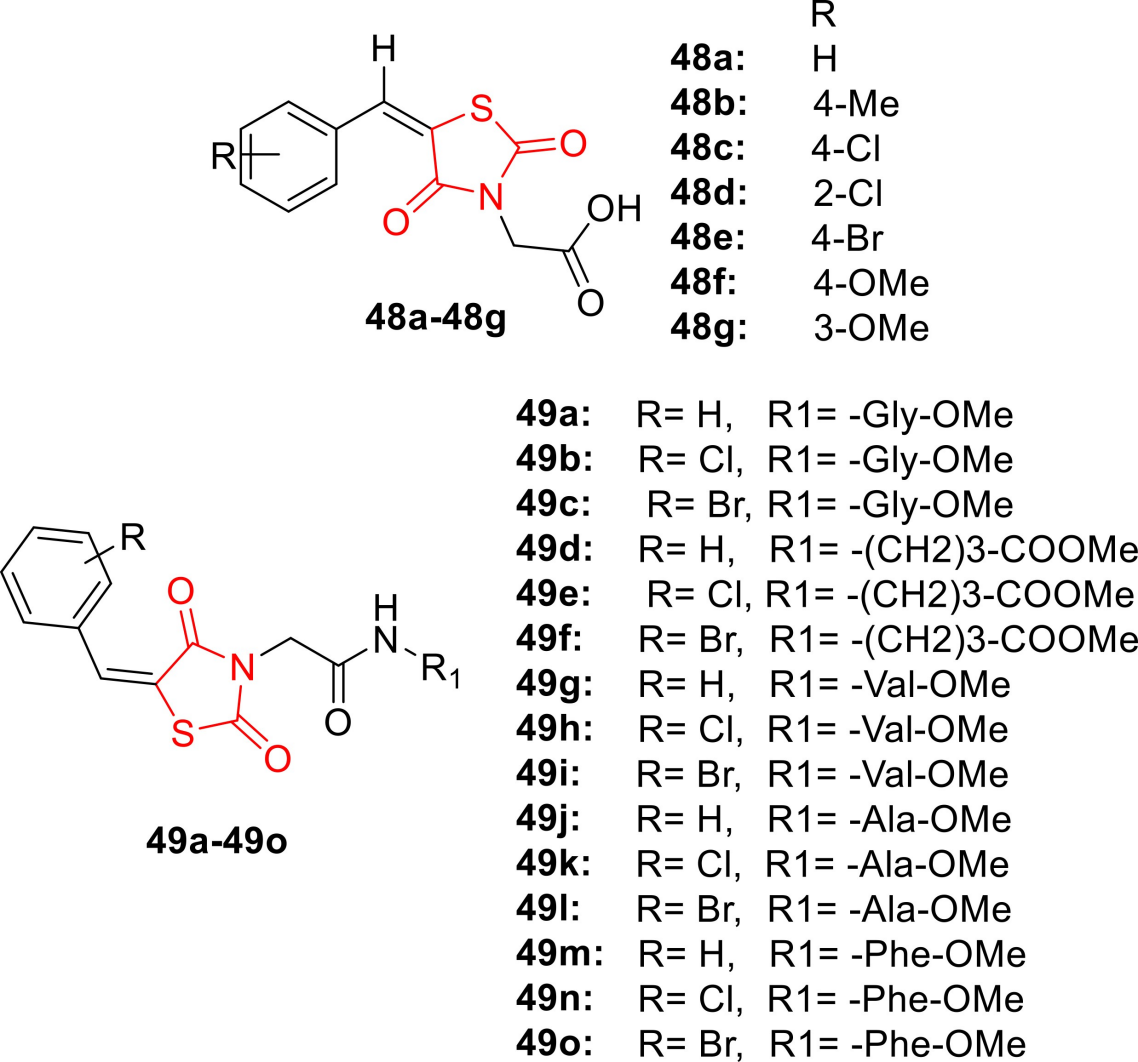
Carboxamide and amino acid containing thiazolidinedione based antimicrobial compounds.

Alhameed *et al*., reported the design and synthesis of a novel series of thiazolidine‐2,4‐dione carboxamide and amino acid derivatives (**48 a**–**48 g** and **49 a**–**49 o**, Figure 39) using the OxymePure/N,N’‐diisopropylcarbodiimide coupling method. The chemical structures of the synthesized compounds were characterized through various spectral techniques, including IR, H‐NMR, C‐NMR, elemental analysis, and LC–MS. The antimicrobial activity of the compounds was assessed against gram‐positive bacteria (*Staphylococcus aureus* and *Bacillus subtilis*), gram‐negative bacteria (*Escherichia coli* and *Pseudomonas aeruginosa*), and the fungal strain *Candida albicans*. The antimicrobial tests revealed that compound **48 g** exhibited the most potent antibacterial activity against *S. aureus* (inhibition zone=20 μM). Additionally, compound **48 a** demonstrated potency against *P. aeruginosa* (inhibition zone=16 mm), while compound **49 k** emerged as the most potent antifungal agent against C. albicans (inhibition zone=18 μM). Notably, none of the derivatives showed significant activity against *B. subtilis* and *E. coli*. In conclusion, the researchers suggested that these derivatives hold potential for further modification and evaluation to enhance their antimicrobial properties.^[102]^

### 4.4 Anti‐Inflammatory Activity

**Figure 40:**
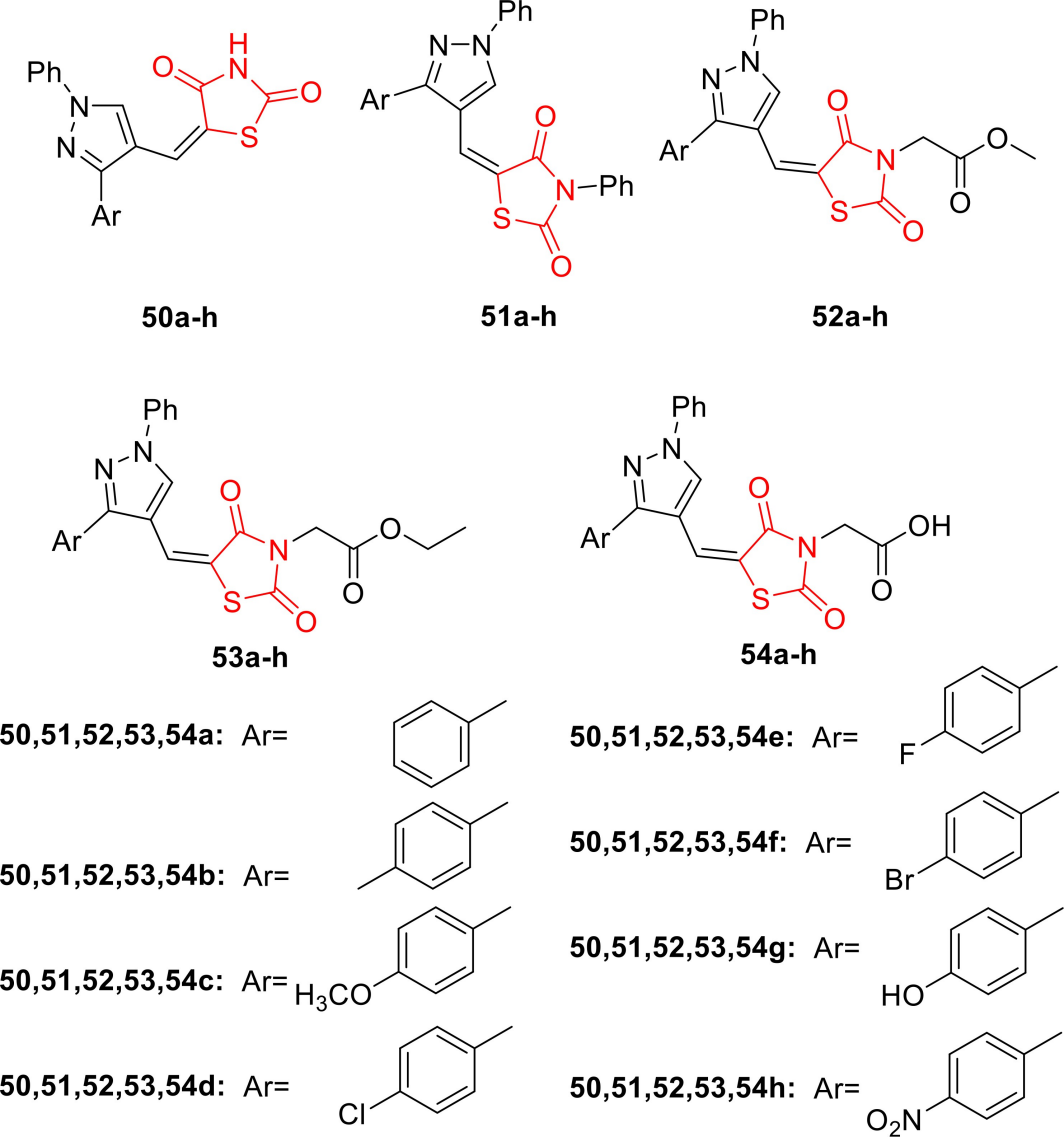
Pyrazolyl 2,4‐thiazolidinedione derivatives as anti‐inflammatory agents.

Aneja *et al*., reported on the design and synthesis of five series of pyrazolyl‐2,4‐thiazolidinediones derivatives (**50 a**–**50 h**, **51 a**–**51 h**, **52 a**–**52 h**, **53 a**–**53 h**, and **54 a**–**54 h**, Figure 40). The synthesized compounds underwent investigation for their *in vivo* anti‐inflammatory activity using the carrageenan‐induced rat paw edema method. Among the 40 compounds studied, 14 compounds (**50 b**, **50 c**, **52 a**, **52 c**, **52 d**, **52 g**, **52 h**, **53 c**, **53 d**, **9 e**, and **54 h**) were either found to be equipotent or more potent than the standard drug indomethacin. Compound **50 f**, **52 f**, **53 c**, and **54 e** exhibited greater potency than indomethacin when comparing percentage inhibition. Molecular docking studies revealed that the activity of one series (**51 a**–**h**) was notably poor compared to the standard, suggesting that the phenyl group at the three‐position of thiazolidine‐2,4‐dione played a crucial role in decreasing the activity of pyrazolyl‐2,4‐thiazolidinediones. The presence of hydrogen, methyl acetate, ethyl acetate, and acetic acid at the nitrogen of TZDS group was suggested to significantly enhance the activity of the synthesized compounds. The researchers further suggested that the potent synthesized compounds exhibited strong binding in the binding site, indicating their potential as COX‐2 inhibitors.

In conclusion, the researchers highlighted the close agreement between *in vivo* and in silico studies of the newly synthesized derivatives, emphasizing their potential for future development as anti‐inflammatory agents.^[103]^

**Figure 41:**
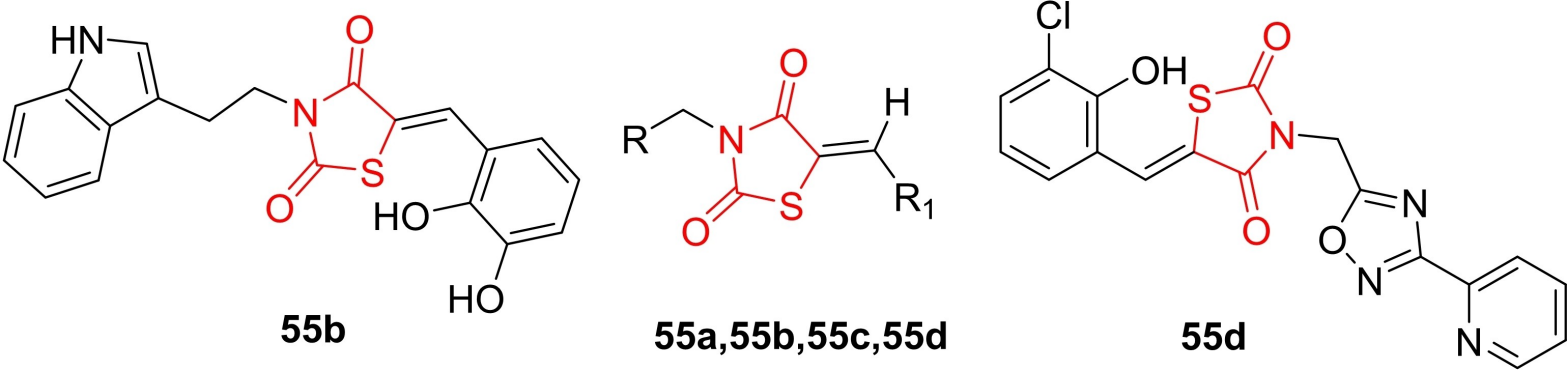
2,4‐thiazolidinedione derivatives based anti‐inflammatory compounds.

Lauro *et al*., conducted a study on the design and synthesis of derivatives based on 2,4‐thiazolidinedione (**55 a**–**55 d**, Figure 41). The synthesized compounds were explored for their anti‐inflammatory activity as dual inhibitors of mPGES‐1 and 5‐LO. To create a diverse library, the researchers employed a large combinatorial approach with a 2,4‐thiazolidinedione chemical core, introducing substitutions at the 3‐ and 5‐ positions. Subsequently, a virtual screening on mPGES‐1 was performed, yielding molecular docking scores. Nine compounds were selected based on this data for further synthesis and evaluation. In the cell‐free mPGES‐1 activity assay, compound **55 d** emerged as the most potent inhibitor, boasting an IC_50_ value of 3.5 μM. Notably, compound **55 d** exhibited highly promising mPGES‐1 inhibitory activity, achieving an 85 % inhibition rate.

Furthermore, the cell‐free 5‐LO activity assay highlighted compound **55 b** as the most potent, with an IC_50_ value of 0.2 μM compared to zileuton (IC_50_=0.6 μM). In conclusion, the integrated approach of virtual screening, straightforward chemical synthesis, and subsequent biological evaluation identified compounds **55 d** and **55 b** as promising candidates for further assessment of their anti‐inflammatory potential through the inhibition of mPGES‐1 and 5‐LO enzymes.^[104]^

**Figure 42:**
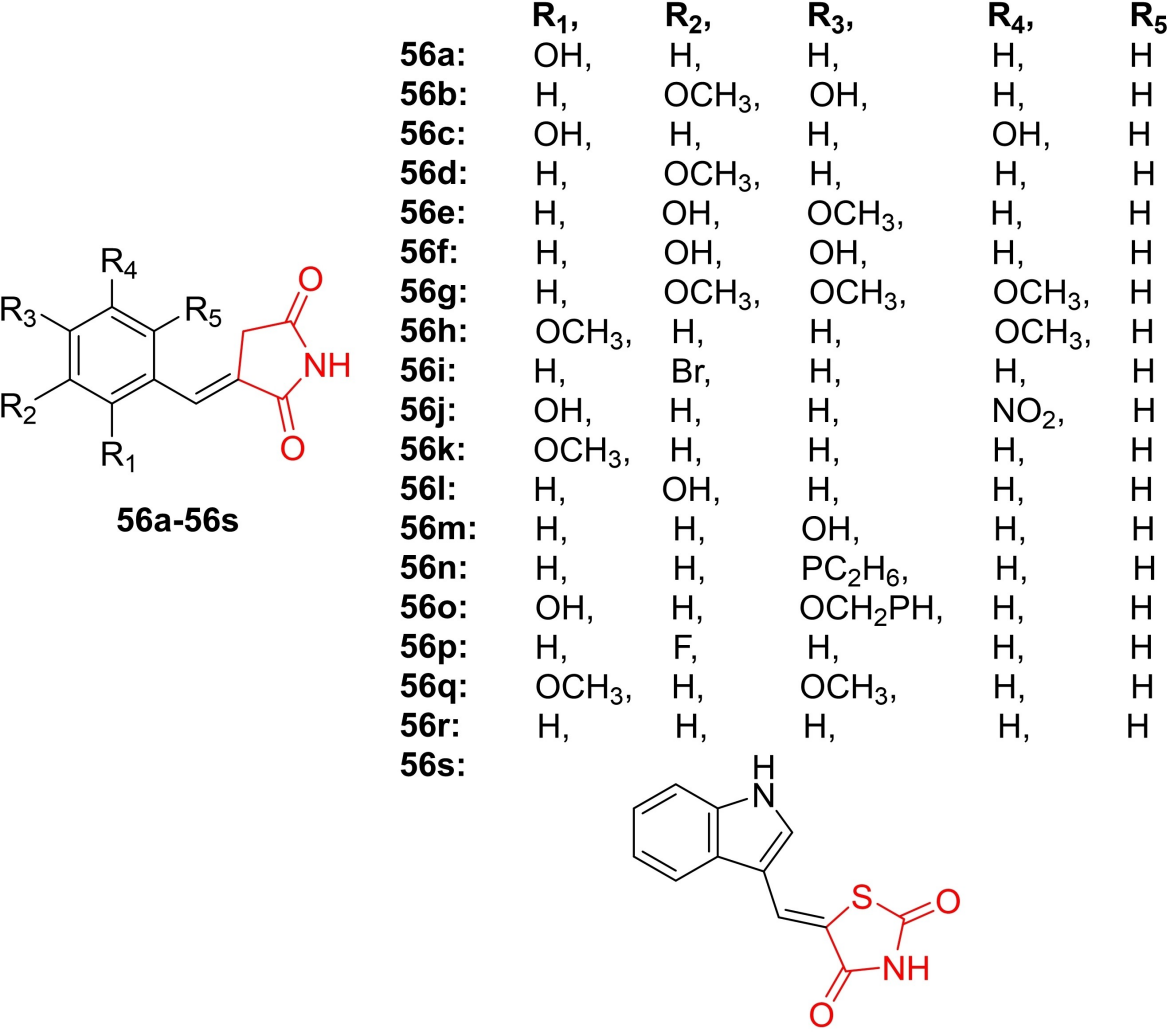
Compounds thiazolidine‐2,4‐dione derivatives against inflammation.

Loncaric *et al*., conducted a study on the design and green synthesis of thiazolidine‐2,4‐dione derivatives (**56 a**–**56 s**, Figure 42) in choline chloride‐based deep eutectic solvents through the Knoevenagel condensation reaction. The primary objective was to investigate the anti‐inflammatory activity of the synthesized derivatives, specifically their inhibition of lipoxygenase. In the lipoxygenase inhibition assay, researchers identified compound **56 c** as the most potent, exhibiting the highest inhibition activity at 76.3 % with an IC_50_ value of 3.52 μM. Additionally, compound **56 d** displayed potent lipid peroxidation inhibition activity, achieving an 84.2 % inhibition rate. The synthesized derivatives demonstrated superior inhibition of the ABTS radical compared to the DPPH radical, with compounds **56 f** and **56 o** exhibiting 100 % inhibition activity against the ABTS radical. Researchers conducted a QSAR study to elucidate the crucial structural features of the synthesized derivatives that contributed to enhanced soybean LOX‐3 inhibition. Molecular docking studies revealed that compound 1c displayed good binding affinity toward the hydrophobic binding pocket of LOX‐3. In conclusion, compound **56 c** emerged as a promising candidate for further evaluation of its anti‐inflammatory potential through the inhibition of the LOX‐3 enzyme.^[105]^

### 4.5 Antitubercular Activity

**Figure 43:**
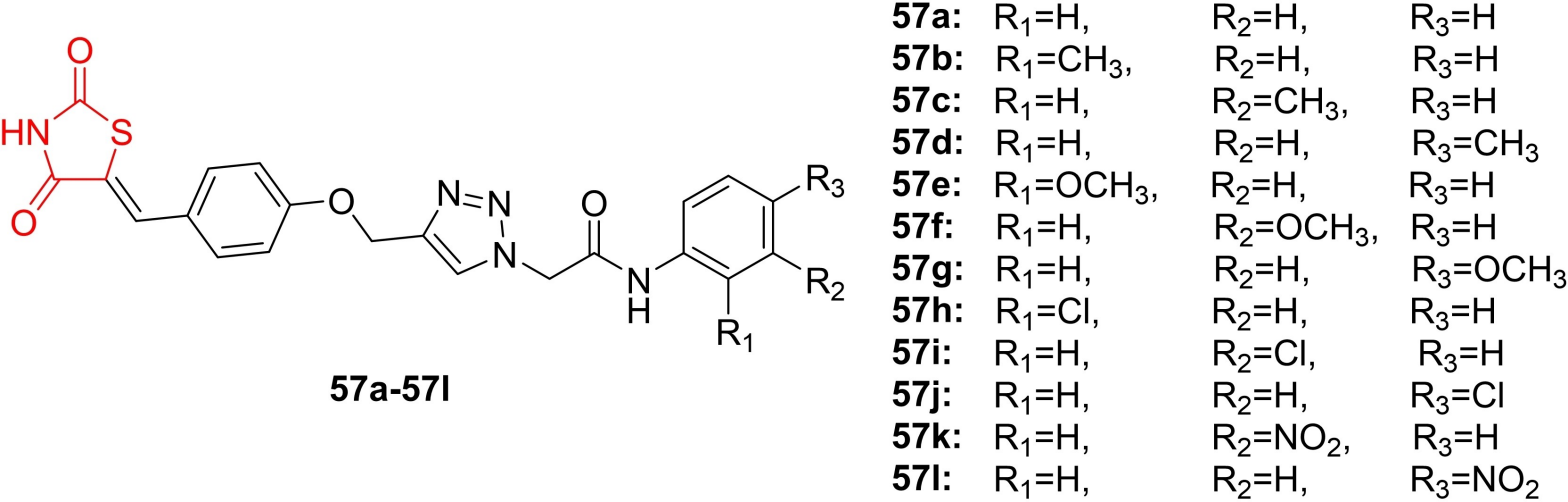
Triazoles‐incorporated 2,4‐thiazolidinedione based antitubercular agents.

Kulkarni *et al*., reported on the design and synthesis of 1,2,3‐triazoles‐incorporated 2,4‐thiazolidinedione conjugates (**57 a**–**57 l**, Figure 43). The synthesized compounds underwent investigation for their antitubercular activity against Mycobacterium bovis BCG (ATCC 35743) and Mycobacterium tuberculosis MTB (ATCC 25177) using the XTT Reduction Menadione Assay (XRMA). Among all the compounds, **57 g**, **57 h**, **57 j**, and **57 l** were identified as the most potent against M. bovis, with IC_90_ values ranging from 1.20 to 2.70, and MTB H37Ra, with IC90 values ranging from 1.24 to 2.65 mg/ml. These results were compared to rifampicin, which had IC_90_ values of 0.0173 (*M. bovis*) and 0.020 (MTB H37Ra). Additionally, these four compounds were screened against different human cancer cells (MCF‐7, HCT 116, and A549). The active compounds demonstrated non‐toxicity against the tested cancer cell lines, with GI_50_/GI_90_ values exceeding 100. A selectivity index study indicated that the potent compounds **57 g**, **57 h**, **57 j**, and **57 l** exhibited the highest selectivity index, exceeding 10 against MCF‐7, HCT 116, and A549, suggesting their potential as prominent antitubercular agents. The researchers also analyzed the antibacterial activity of the potent compounds (**57 g**, **57 h**, **57 j**, and **57 l**) against two Gram‐negative strains (*P. fluorescens* and *E. coli*) and two Gram‐positive strains (*B. subtilis* and *S. aureus*).

The compounds demonstrated inactivity against the bacterial strains, indicating high selectivity toward MTB and BCG strains. The researchers concluded that compounds **57 g**, **57 h**, **57 j**, and **57 l** have the potential for further development as antitubercular agents.^[106]^

**Figure 44:**
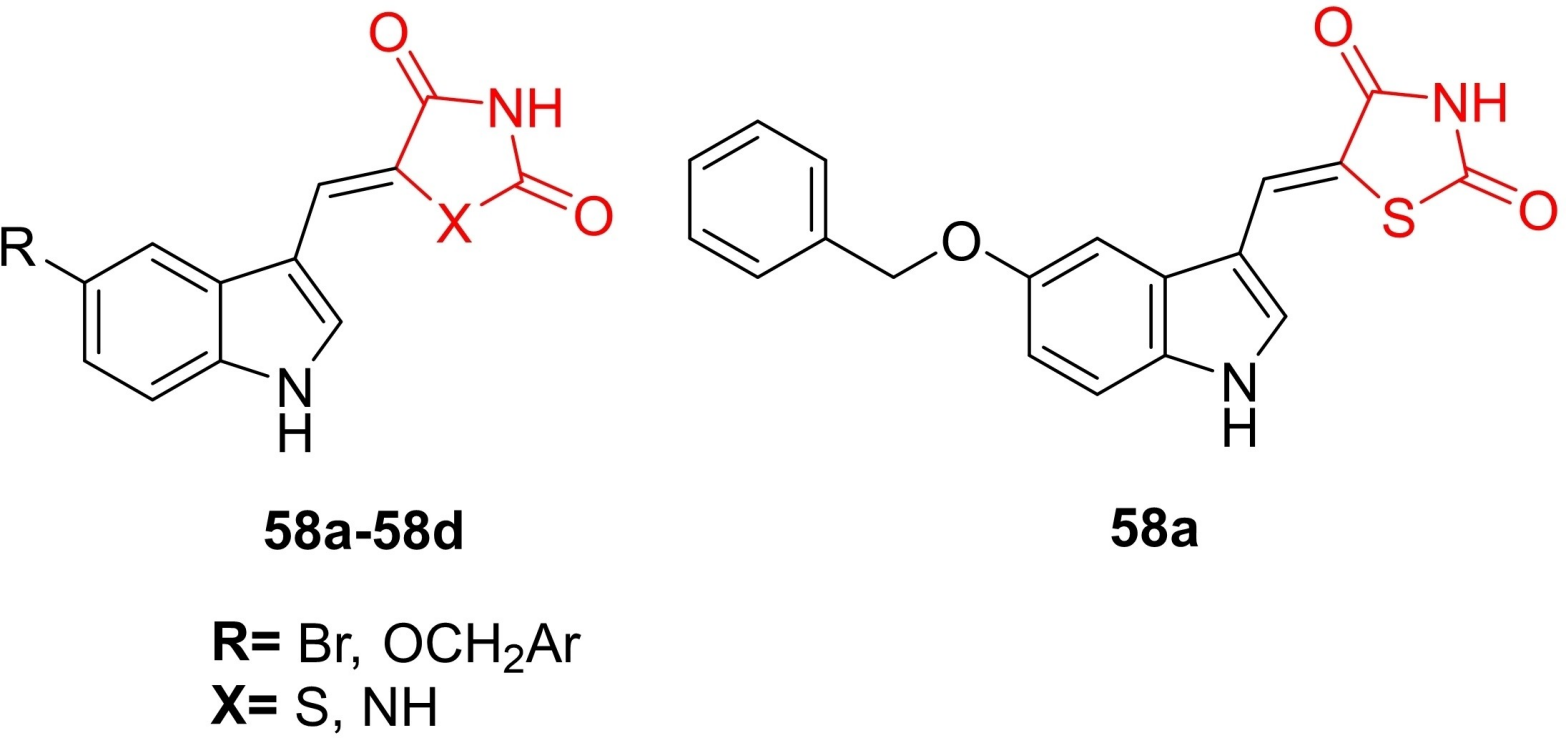
Hydantoin containing thiazolidine‐2,4‐dione based antimycobacterial agents.

Angelova *et al*., reported the design and synthesis of a series of new thiazolidine‐2,4‐dione and hydantoin derivatives (**58 a**–**58 d**, Figure 44) through Knoevenagel condensation, characterizing their chemical structures using H‐NMR, C‐NMR, and HR‐MS techniques. The synthesized derivatives were examined for their antimycobacterial activity against Mycobacterium tuberculosis H37Rv. In the resazurin microtiter assay (REMA), compound **IIIa** emerged as the most potent among all, with a MIC value of 0.7505 μM, surpassing ethambutol (MIC=2.0024 μM) and isoniazid (MIC=1.8234 μM). Additionally, researchers noted that compound IIIa exhibited low cytotoxicity in the human embryonic kidney cell line HEK‐293T (IC_50_>200). Molecular docking studies revealed that compound **58 a** displayed remarkable binding affinity (−7.67 kcal/mol) toward the active binding site of KasA.

The researchers also emphasized that compound **58 a** met desired physicochemical, pharmacokinetic, and drug‐likeness properties. In conclusion, the researchers suggested that compound **58 a** could be further refined and evaluated for its antimycobacterial potential.^[107]^

### 4.6 Miscellaneous

**Figure 45:**
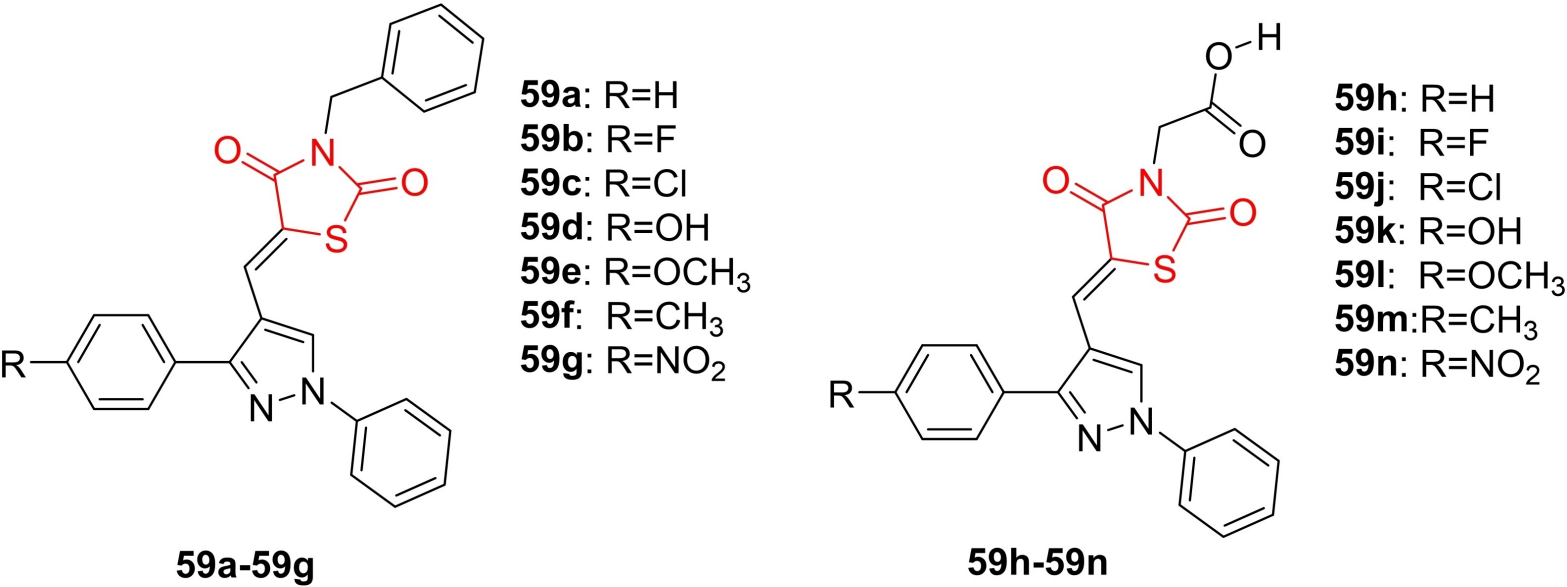
Pyrazole‐incorporated thiazolidine‐2,4‐dione based antioxidant compounds.

Bansal *et al*., reported the design and synthesis of a series of fourteen novel thiazolidine‐2,4‐dione‐pyrazole conjugates (**59 a**–**59 g and 59 h**–**59 n** Figure 45). The synthesis involved Knoevenagel condensation and N‐substitution using benzyl bromide and bromoacetic acid, respectively. The reaction progress was monitored by thin layer chromatography, and the compounds were characterized through physicochemical and spectrophotometric analysis. The synthesized compounds underwent investigation for their antidiabetic activity against STZ‐NA‐induced diabetes in mice, *in vitro* anti‐inflammatory and antioxidant activities using the DPPH method, and docking against peroxisome proliferator receptors (PPAR‐γ) and alpha‐amylase. Compound **59 n** emerged as the most potent, demonstrating a significant blood glucose‐lowering effect (134.46 μg/dl) compared to pioglitazone (136.56 μg/dl). Additionally, **59 n** exhibited active inhibition of alpha‐amylase (IC_50_=4.08 μ/ml) compared to acarbose (IC_50_=8.0 μ/ml). Among the compounds, **59 g** displayed the most potent anti‐inflammatory activity, reducing inflammatory markers (TNF‐α, IL‐β, MDA). Antioxidant activity assessed by the DPPH method revealed that compounds **59 d**, **59 e**, and **59 h** exhibited prominent results with IC_50_ values of 110.88, 127.18, and 128.55 μ/ml, respectively, compared to the standard drug acarbose (81.12 μ/ml). In conclusion, the researchers noted that these compounds, with their favorable activity profiles, could serve as novel leads for future investigations.^[108]^

**Figure 46:**
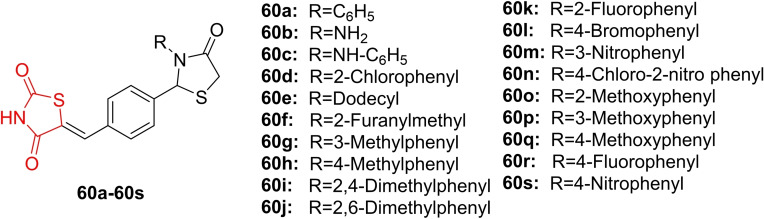
Novel series of thiazolidine‐2,4‐dione derivatives‐based antioxidant agents.

Kumar *et al*., reported the design and synthesis of a novel series of thiazolidine‐2,4‐dione derivatives (**60 a**–**60 s**, Figure 46), determining their chemical structures through physicochemical parameters and spectral techniques such as IR, MS, and H‐NMR. The synthesized compounds underwent evaluation for their antimicrobial, antioxidant, and anticancer activities. Antimicrobial activity was assessed using the serial tube dilution method against *Staphylococcus aureus*, *Bacillus subtilis*, *Escherichia coli*, *Candida albicans*, and *Aspergillus niger*, with fluconazole and cefadroxil as reference antifungal and antibacterial drugs. Compounds **60 e**, **60 m**, **60 o**, and **60 r** displayed promising activity, with MIC ranging from 7.3 to 26.3.

DPPH free radical scavenging activity was employed to assess antioxidant potential, and compound **60 e** (IC_50_=14.85) emerged as the most potent antioxidant. Additionally, the MTT assay against DU‐145 cancer cell lines was performed to evaluate the anticancer potential of compounds **60 b**, **60 j**, and **60 k**, revealing mild anticancer activity for all screened derivatives. Researchers concluded by conducting ADME studies, determining that all compounds exhibited drug‐like and orally active properties. The findings suggest that these compounds warrant further investigation for their potential in anticancer, antimicrobial, and antioxidant activities.^[109]^

**Figure 47:**
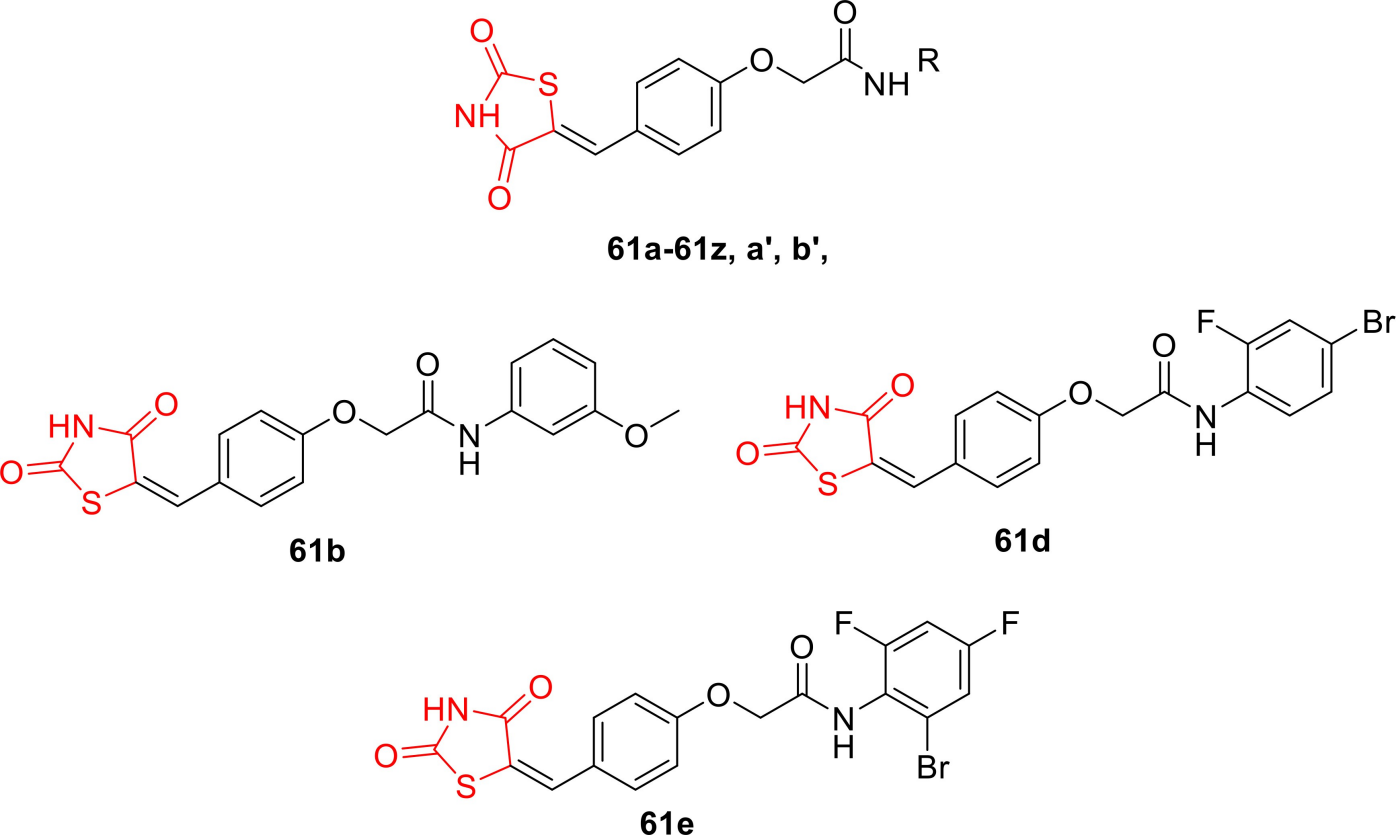
Benzylidene clubbed 2,4‐thiazolidinedione derivatives‐based antihyperlipidemic compounds.

Patil *et al*., reported the design and synthesis of derivatives (**61 a**–**61 z**, **a’**, **b’**, Figure 47) of 5‐benzylidene‐2,4‐thiazolidinedione, with confirmed structures using various spectroscopic techniques including FTIR, H NMR, C NMR, and mass spectrometry. The synthesized derivatives were assessed for their antihyperglycemic and antihyperlipidemic properties in diabetic rats induced by a high‐fat diet and low doses of streptozotocin. Seven biochemical parameters, including blood glucose, triglycerides, cholesterol, creatinine, blood urea nitrogen, HDL‐cholesterol, and glycosylated hemoglobin, were analyzed in serum by standard methods. Compound **61 b**, **61 d** and **61 e** exhibited potent antihyperglycemic and antihyperlipidemic effects, with activity exceeding 80 %, compared to the positive control pioglitazone. Cell viability assessment using MTT reduction assay demonstrated that the derivatives were non‐toxic to normal hepatocytes.

Additionally, histopathological examination of the liver and heart for compounds **61 b**, **61 d**, and **61 e** revealed no significant toxicity. Transactivation assays for **61 b** and **61 d** indicated a fold activation of 17 % and 25 %, respectively, of PPAR compared to pioglitazone. In conclusion, researchers identified compounds **61 b** and **61 d** as promising candidates for further development as antihyperglycemic and antihyperlipidemic agents.^[110]^

**Figure 48:**
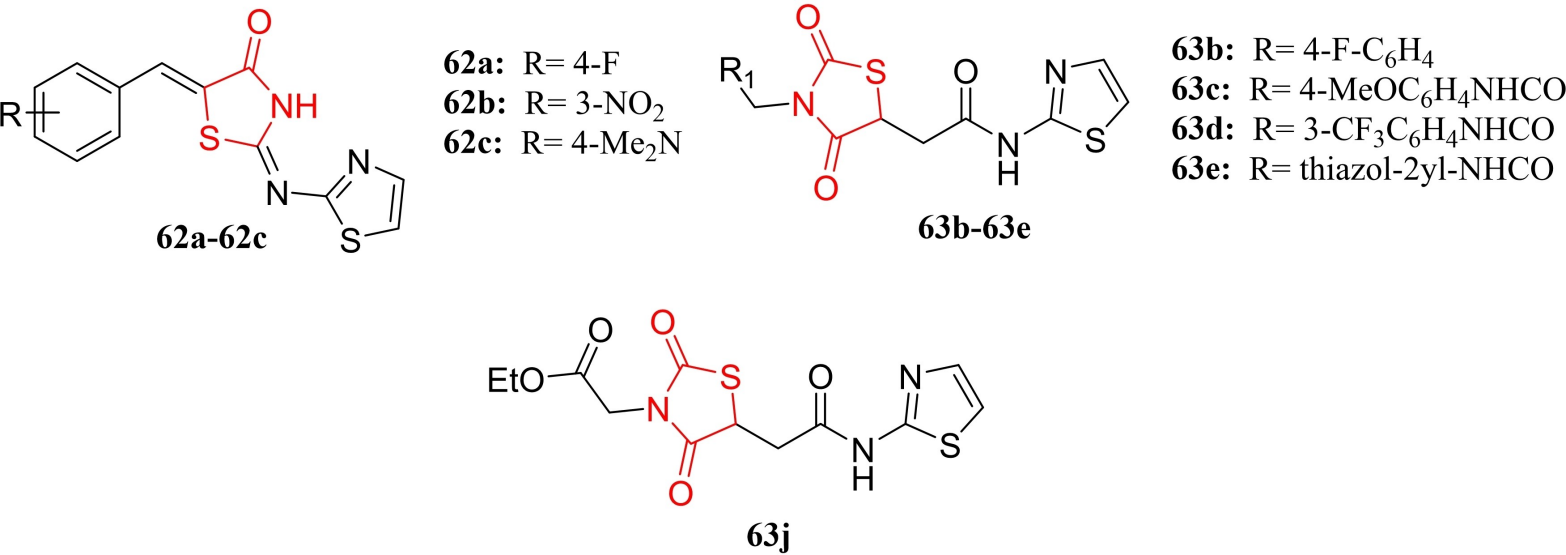
Imino and carboxylic acid‐appended 2,4‐thiazolidinone based anticonvulsant compounds.

Mishchenko *et al*., reported the design and synthesis of thiazole‐bearing hybrids based on 2‐imino‐4‐thiazolidinone and 2,4‐dioxothiazolidine‐5‐carboxylic acid cores (**62 a**–**62 c**, **63 b**–**63 e**, **63 j**, Figure 48). These compounds were synthesized through Knoevenagel reaction, alkylation reaction, and a one‐pot three‐component reaction. The synthesized compounds underwent evaluation for their anticonvulsant activity using two models: pentylenetetrazole‐induced seizures and maximal electroshock seizure tests. Compounds **62 b**, **63 d**, and **63 j** were identified as the most potent in the PTZ model. Researchers further assessed the three potent compounds for their anticonvulsant action in the MES model, using carbamazepine as a reference drug. They found that compounds **62 b**, **63 d**, and **63 j** decreased the duration of tonic seizures by 3, 1.8, and 2.4 times, and clonic seizures by 2.9, 3.5, and 2.5 times, respectively. The potent compounds were also evaluated for their LD50 value in mice, and researchers revealed that these compounds are non‐toxic and well‐tolerated in animals. Researchers discussed the structure‐activity relationship of **62 b**, **63 d**, and **63 j**, suggesting that these compounds have a mixed mechanism of action aimed at increasing inhibitory processes in the CNS by enhancing GABAergic activity and reducing excitatory processes by blocking sodium channels.

In conclusion, Mishchenko and team proposed that compounds **62 b**, **63 d**, and **63 j** warrant further investigation for their anticonvulsant activity.^[111]^

**Figure 49:**
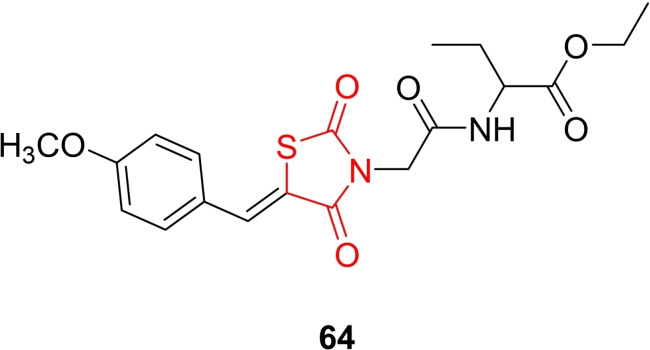
Arylidine‐incorporated 2,4‐thiazolidinedione based anticorrosion compounds.

Johnstone *et al*., reported the design and synthesis of ethyl‐[2‐(5‐arylidine‐2,4‐dioxothiazolidin‐3‐yl) acetyl] butanoate, a novel thiazolidinedione derivative (**64**, Figure 49). The synthesized compound B1 was investigated for its corrosion inhibition effect on mild steel in 1 M HCL using gravimetric analysis, electrochemical analysis, and Fourier‐transform infrared spectroscopy at five concentrations (5E‐5 M to 9E‐5 M). Researchers characterized the compound **64** using nuclear magnetic resonance spectroscopy.

Gravimetric analysis was performed at four different temperatures (303.15 K, 313.15 K, 323.15 K, 333.15 K), revealing a maximum percentage inhibition efficiency of 92 % at 303.15 K. Electrochemical analysis conducted at 303.15 K showed an inhibition efficiency of 83 %. Thermodynamic parameters such as delta G were calculated, indicating that compound **64** absorbed onto the mild steel surface via a mixed type of action at lower temperatures, transitioning to exclusively chemisorption at higher temperatures. In conclusion, researchers suggested that compound **64** warrants further investigation for its corrosion inhibitory effect.^[112]^

**Figure 50:**
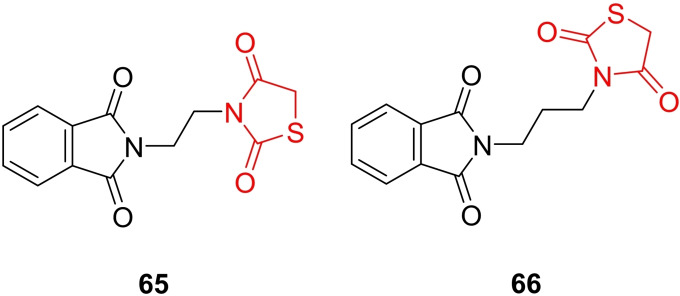
Thiazo‐isoindolinedione clubbed 2,4‐thiazolidinedione based antiviral agents.

Shaaban *et al*., reported the design and synthesis of novel thiazo‐isoindolinedione derivatives (**65 and 66**, Figure 50) through the reaction of thiazolidinedione and isoindoline‐dione, achieving good yields (up to 92 %). The researchers characterized the chemical structures of the synthesized derivatives using IR, H‐NMR, C‐NMR, and MS techniques. These derivatives were then scrutinized for their inhibitory potential against the SARS‐CoV‐2 main protease (Mpro). In molecular docking studies, compound **66**, featuring a propylene bridge between 1,3‐dioxoisoindoline and thiazolidine‐2,4‐dione, exhibited higher potency than compound **65**, which had an ethylene bridge, as a SARS‐CoV‐2 Mpro inhibitor. The researchers highlighted that compound **66** demonstrated a more profound fit within the SARS‐CoV‐2 Mpro target receptor. In conclusion, the researchers suggested that compound **66** holds promise for further modification and evaluation as an antiviral agent targeting SARS‐CoV‐2 Mpro.^[113]^

**Figure 51:**
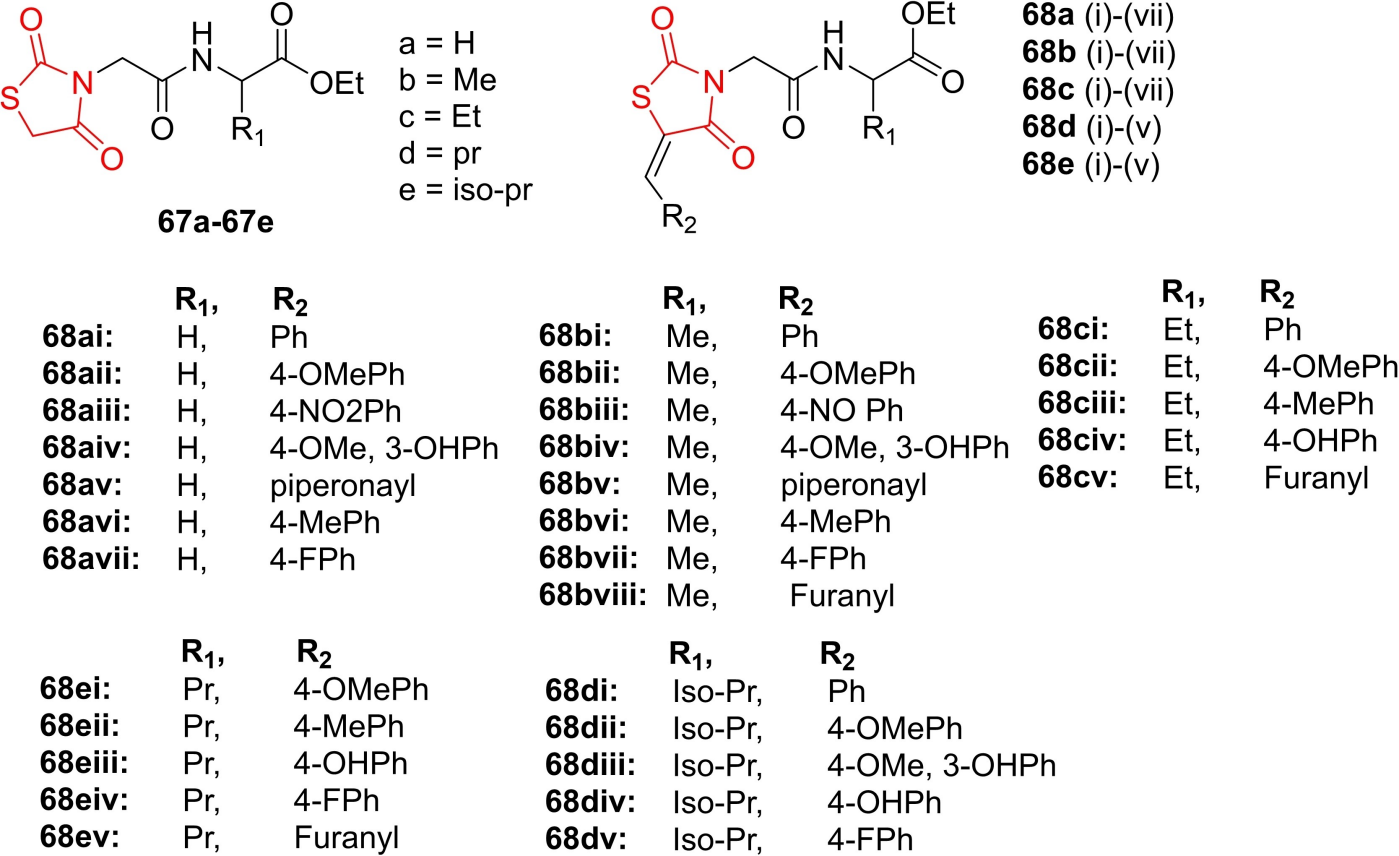
Arylidene containing 2,4‐thiazolidinedione based therapeutic agents.

Tshiluka *et al*., presented a study outlining the design and synthesis of novel 5‐arylidene‐2,4‐thiazolidinedione esters {**67 a**–**67 e**, **68a**(**i**–**vii**), **68b**(**i**–**vii**), **68c**(**i**–**vii**), **68d**(**i**–**v**), **68e**(**i**–**v**), Figure 51). The researchers employed a four‐step synthesis process to obtain ethyl‐(2‐(5‐arylidine‐2,4‐dioxothiazolidin‐3yl)acetyl)glycinates (**68 a**), alaninates (**68 b**), butanoates (**68 c**), valinates (**68 d**), and norvalinates (**68 e**) derivatives. The average yields for these derivatives were reported as 52 %, 54 %, 14 %, 14 %, and 28 %, respectively. The synthesis procedure commenced with the protection of five distinct amino acids (9) as esters (10) using thionyl chloride in the presence of ethanol at low temperatures (0–5 °C). Subsequently, compound 10 was treated with bromoacetyl chloride to yield ethyl (2‐chloroacetamido) esters (11).

The 1,3‐thiazolidine‐2,4‐dione was then converted into its potassium salts, and these salts were reacted with ethyl (2‐chloroacetamido) esters to produce compound **67** (**67 a**, **67 b**, **67 c**, **67 d**, and **67 e**) in favorable yields. In the final step, researchers subjected compound **67** to a Knoevenagel condensation reaction with various aldehydes to generate different compounds (**68 a**, **68 b**, **68 c**, **68 d**, and **68 e**). In conclusion, the authors suggested that the newly synthesized derivatives hold promise for further exploration of their diverse therapeutic potentials.^[114]^

## 5 Conclusions

This review primarily delves into the advancements in the medicinal chemistry of 2,4‐thiazolidinedione (TZD) derivatives, exploring their activities and therapeutic potential across various therapies. Initially utilized for Type 2 diabetes treatment, exemplified by rosiglitazone and pioglitazone, TZDs have exhibited adverse effects over time, including weight gain, oedema, fractures, and congestive heart failure. Consequently, this review aims to highlight the heightened antidiabetic efficacy and reduced side effects of TZDs. Furthermore, it discusses the synthesis and structural modifications of TZDs, leading to enhanced activities and improved drug‐likeness properties. A broad spectrum of therapeutic activities is attributed to TZD derivatives, encompassing anticancer, antimicrobial, anti‐inflammatory, antidiabetic, antioxidant, antihyperlipidemic, anticonvulsant, and antitubercular properties. The review incorporates outcomes from various in‐vivo, in‐vitro, and in‐silico studies to substantiate the aforementioned potentials and safety profiles of TZD derivatives. Significantly, computational studies have played a pivotal role in elucidating the unique and robust interactions of derivatives with receptors, as well as predicting their pharmacokinetic profiles and bioavailability. Synthesis approaches and steps to obtain novel TZD derivatives are also explored within this review, reflecting both the progress achieved thus far and the challenges yet to be overcome in realizing the full potential of TZD derivatives. Moving forward, it advocates for future research efforts to concentrate on minimizing side effects while enhancing the therapeutic potential of novel 2,4‐thiazolidinedione derivatives.

## Conflict of Interests

The authors declare no conflict of interest.

6

## Biographical Information

*Sneha Gupta is a Bachelor of Pharmacy graduate from the School of Pharmaceutical Sciences, Lovely Professional University. Her research interests include the development and evaluation of novel therapeutic agents. She has been actively involved in several research projects during her undergraduate studies*.

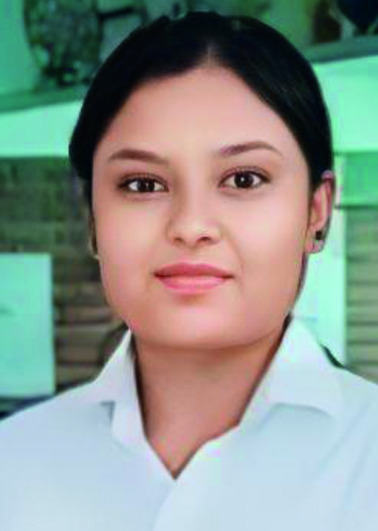

## Biographical Information

*Sumeet Jha holds a Bachelor of Pharmacy degree from the School of Pharmaceutical Sciences, Lovely Professional University. His primary research focus is on medicinal chemistry and drug design. Sumeet has contributed to various research articles and projects during his academic career*.

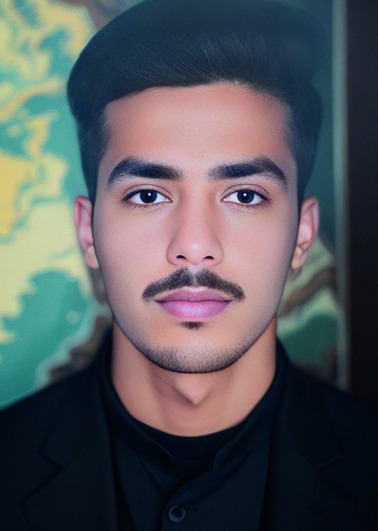

## Biographical Information

*Supriya Rani is a Bachelor of Pharmacy graduate from the School of Pharmaceutical Sciences, Lovely Professional University. She has a keen interest in pharmaceutical research and has participated in multiple research initiatives. Supriya aims to pursue further studies in pharmaceutical sciences*.

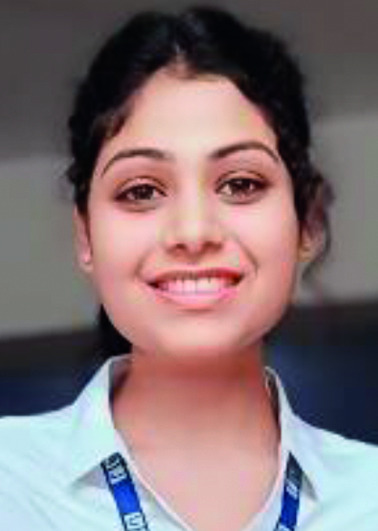

## Biographical Information

*Pinky Arora is currently pursuing a PhD in Biochemistry at the School of Bioengineering and Biosciences, Lovely Professional University. She has published several research articles in reputed journals, focusing on biochemistry and molecular biology. Pinky's research aims to understand complex biological processes and develop innovative solutions*.

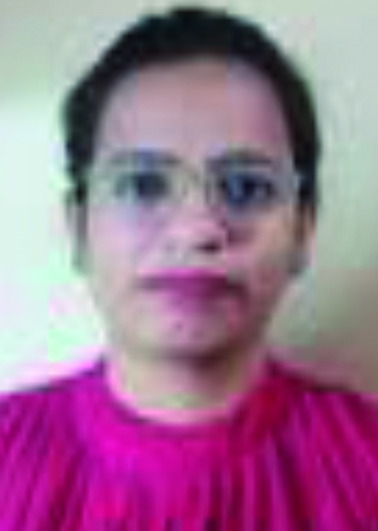

## Biographical Information

*Dr. Shubham Kumar holds a PhD in Pharmaceutical Chemistry from Lovely Professional University, where he currently serves as a faculty member in the School of Pharmaceutical Sciences. He has published various research articles in reputed journals and has a strong background in the synthesis and evaluation of therapeutic molecules. Shubham's research interests include drug design and development for cancer treatment*.

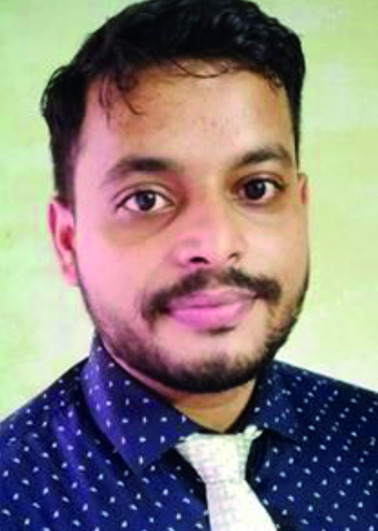

## Data Availability

Data sharing is not applicable to this article as no new data were created or analyzed in this study.
